# Gene expression profiling identifies distinct molecular subgroups of leiomyosarcoma with clinical relevance

**DOI:** 10.1038/bjc.2016.280

**Published:** 2016-09-08

**Authors:** Yin-Fai Lee, Toby Roe, D Chas Mangham, Cyril Fisher, Robert J Grimer, Ian Judson

**Affiliations:** 1 Institute of Cancer Research, 15 Cotswold Road, Belmont, Sutton, London SM2 5NG, UK; 2 Department of Pharmacology and Pharmacy, The University of Hong Kong, Hong Kong, China; 3 Department of Pathology, The Royal Orthopaedic Hospital NHS Trust, Birmingham B31 2AP, UK; 4 Sarcoma Unit, The Royal Marsden Hospital NHS Trust, Fulham Road, London SW3 6JJ, UK

**Keywords:** leiomyosarcoma, microarray, gene expression, molecular classification, clustering, soft tissue sarcoma

## Abstract

**Background:**

Soft tissue sarcomas are heterogeneous and a major complication in their management is that the existing classification scheme is not definitive and is still evolving. Leiomyosarcomas, a major histologic category of soft tissue sarcomas, are malignant tumours displaying smooth muscle differentiation. Although defined as a single group, they exhibit a wide range of clinical behaviour. We aimed to carry out molecular classification to identify new molecular subgroups with clinical relevance.

**Methods:**

We used gene expression profiling on 20 extra-uterine leiomyosarcomas and cross-study analyses for molecular classification of leiomyosarcomas. Clinical significance of the subgroupings was investigated.

**Results:**

We have identified two distinct molecular subgroups of leiomyosarcomas. One group was characterised by high expression of 26 genes that included many genes from the sub-classification gene cluster proposed by Nielsen *et al.* These sub-classification genes include genes that have importance structurally, as well as in cell signalling. Notably, we found a statistically significant association of the subgroupings with tumour grade. Further refinement led to a group of 15 genes that could recapitulate the tumour subgroupings in our data set and in a second independent sarcoma set. Remarkably, cross-study analyses suggested that these molecular subgroups could be found in four independent data sets, providing strong support for their existence.

**Conclusions:**

Our study strongly supported the existence of distinct leiomyosarcoma molecular subgroups, which have clinical association with tumour grade. Our findings will aid in advancing the classification of leiomyosarcomas and lead to more individualised and better management of the disease.

Adult soft tissue sarcomas are malignant tumours that occur in connective tissues throughout the body, other than the bone or cartilage (Goldblum *et al*, 2014). Major histologic categories of soft tissue sarcomas include leiomyosarcoma (smooth muscle differentiation), liposarcoma (fatty differentiation), rhabdomyosarcoma (striated muscle differentiation), synovial sarcoma and undifferentiated pleomorphic sarcoma. These tumours are heterogeneous and a major complication in the management of this disease is that the existing classification scheme is not definitive and is still evolving. Leiomyosarcomas are malignant tumours with smooth muscle differentiation that are defined as a single group based on the morphology and immunohistochemical examination, although exhibiting a wide range of clinical behaviour that correlates to some extent with histological grade (Goldblum *et al*, 2014). Gastrointestinal stromal tumour (GIST) was historically classified as gastrointestinal leiomyosarcoma before the identification of activating mutations in *KIT* by Hirota *et al* (1998) and the immunohistochemical association, with interstitial cells of Cajal by Kindblom and others (Kindblom *et al*, 1998) in the late 1990s. Advances in molecular understanding and techniques clearly distinguished GIST from leiomyosarcoma, and with the advent of imatinib therapy this became a paradigm for the molecularly targeted treatment of cancer (Verweij *et al*, 2004; Bauer and Joensuu, 2015; Nishida *et al*, 2015). This clearly illustrates the importance of improving the classification of sarcomas and the beneficial effect this can have on disease management and patient outcome. With further advances in modern molecular techniques, it is hoped that we would be able to better molecularly classify the tumours into subgroups, which may eventually lead to improved management of the disease. To this end, we have used microarray gene expression profiling to study the expression pattern of leiomyosarcomas, with the aim of improving its molecular classification and identifying new molecular subgroups that may be of clinical relevance. We have focused on leiomyosarcomas of extra-uterine origin in this study, as uterine leiomyosarcoma represents a relatively distinct clinical entity and can be easily identified from its site of origin (Amant *et al*, 2009; Linch *et al*, 2014). Our findings provide important support to the initial findings of Nielsen *et al* (2002) in an independent sarcoma set.

Nielsen *et al* (2002) used microarrays to molecularly characterise 41 soft tissue tumours that included some leiomyosarcomas and GISTs, as well as tumours of other histologic categories. They noted a distinct gene expression pattern of the GISTs that was distinct from the leiomyosarcomas. Eleven leiomyosarcomas were included in their study, and they found initial evidence of separation of leiomyosarcomas into two subgroups, one group characterised by the high expression of a group of 24 gene, representing 20 distinct genes that included the gene for Calponin and other genes implicated in muscle structure and function. Recently, coinciding with our investigations, other studies also suggested the existence of subtypes of leiomyosarcoma (Beck *et al*, 2010; Guo *et al*, 2015). In this study, we further investigated the molecular classification of leiomyosarcomas and the clinical relevance of the new classification.

## Materials and methods

### Microarray procedures

Leiomyosarcoma is a relatively rare form of cancer. Sarcoma tissue samples used in this study were collected from patients undergoing surgery at two hospitals (the Royal Marsden NHS Foundation Trust, London and the Royal Orthopaedic Hospital NHS Foundation Trust, Birmingham) that treat significant numbers of sarcoma patients in the UK over the period of 1987–2001. Diagnoses were performed by experienced sarcoma pathologists using conventional criteria, including immunohistochemistry and electron microscopy, if appropriate. This study was conducted with the approval of our local Research Ethics Committee. Clinicopathologic descriptions of the cases are shown in Supplementary Table S2. (Summary patient characteristics: median age=61 years; sex (M=58% and F=42%); location (extremity=58%, non-extremity (thoracic/abdominal/retroperitoneal sites)=42%); and tumour size (5 cm or less in greatest dimension=21%, >5 cm in greatest dimension=79%).) The tumour samples were snap-frozen in liquid nitrogen and stored at –80 °C until RNA extraction. Tumour and control RNA preparation were performed as in Lee *et al* (2003), except that HB4a was not included in the control RNA. Microarray slides were gridded with 11 622 spots, consisting of 1937 I.M.A.G.E cDNA clones (gridded six times each) acquired from the UK Human Genome Mapping Project Resource Centre and Research Genetics (http://www.resgen.com). The majority of the clones included were selected at random, except for the 23 gene probes that corresponded to 18 of the distinct genes reported by Nielsen *et al* (2002), as described below. We were unable to get hold of the clones that corresponded to the other two of the 20 distinct genes that distinguished their leiomyosarcoma samples into the two subgroups. Information on the gene set can be found at the Supplementary Table S1 (array data to be found on Gene Expression Omnibus (GEO) GSE76216). The preparation of the microarray slides, including gridding and blocking were as described in Clark *et al* (2002), except that the BioRobotics Microgrid II (BioRobotics, Cambridge, UK) was used for gridding in this study. RNA labelling was performed as in Lee *et al* (2003), except that after the final wash with 400 *μ*l 0.5 × SSPE, the sample was reduced to a volume of 4 *μ*l.

Microarray hybridisation was performed as in Lee *et al* (2003) except the following: after the coverslip fell off, the slide was then washed with 4 × SSPE, 10 mM EDTA for 1 min at 42 °C; then 50% formamide, 6 × SSPE at 42 °C for 15 s with gentle rocking; then 2 × SSPE, 10 mM EDTA for 30 s at room temperature; and 0.1 × SSPE for 30 s at room temperature. The slide was then rinsed briefly with HPLC grade water and dried with canned air.

Hybridised microarray slides were scanned in a GenePix 4000B scanner (Axon Instruments, Foster City, CA, USA) as described before (Lee *et al*, 2004) and slides were scanned at photomultiplier tube voltage levels that provided a Cy5 : Cy3 hybridisation ratio across the slide of roughly 1.

### Analysis of microarray data

Data processing and analyses were performed with the GenePix Pro software (Axon Instruments, Foster City, CA, USA) and the GeneSpring software (Silicon Genetics, Redwood City, CA, USA). Background determination and flagging were performed as described before (Lee *et al*, 2004). Further quality filtering was performed by excluding spots with fluorescent spot intensity in the control channel being <1.4 times the local background of that channel. Ratios of fluorescent intensities (Cy5 : Cy3) for individual cDNA were then determined after subtraction of background as described before (Lee *et al*, 2004), except that the average fluorescent intensity ratios of replicate spots were used. These ratios were then normalised by making the median of all measurements in each sample to be 1. The resulting ratios were further normalised so that the median of all measurements taken for a particular gene is 1. To obtain genes that showed variation in expression among the tumours, a subset of genes that had normalised expression ratios of >2 in at least five of the samples or <0.5 in at least five of the samples were selected. Hierarchical clustering was then applied to the log-transformed data for these genes and the tumours, using average-linkage clustering with Pearson correlation around zero as the similarity metric. Only genes with expression data present in half or more of the tumour samples, following quality filtering were included in the gene clustering. Clustering analysis was also performed with the ‘leio-subclass' genes from Nielsen *et al* (2002) present in our array, as well as gene clusters identified from our data set. Class comparison analysis (BRB array tools) using two sample *t*-test and multivariate permutations test computed based on 1000 random permutations (confidence level of false discovery rate assessment: 80% maximum allowed proportion of false-positive genes: 0.1) was carried out to identify statistically significant genes associated with the group I and II tumours.

Gene expression data of the 17 leiomyosarcomas reported in the study by Baird *et al* (2005) were downloaded from GEO data repository. Array data normalisation and clustering analysis were performed as described above.

Other statistical analyses including Kendall's tau_b_ for assessing the significance of association between tumour subgroupings and tumour grade were performed using SPSS (SPSS Inc, Chicago, IL, USA).

## Results

**Figure 1:**
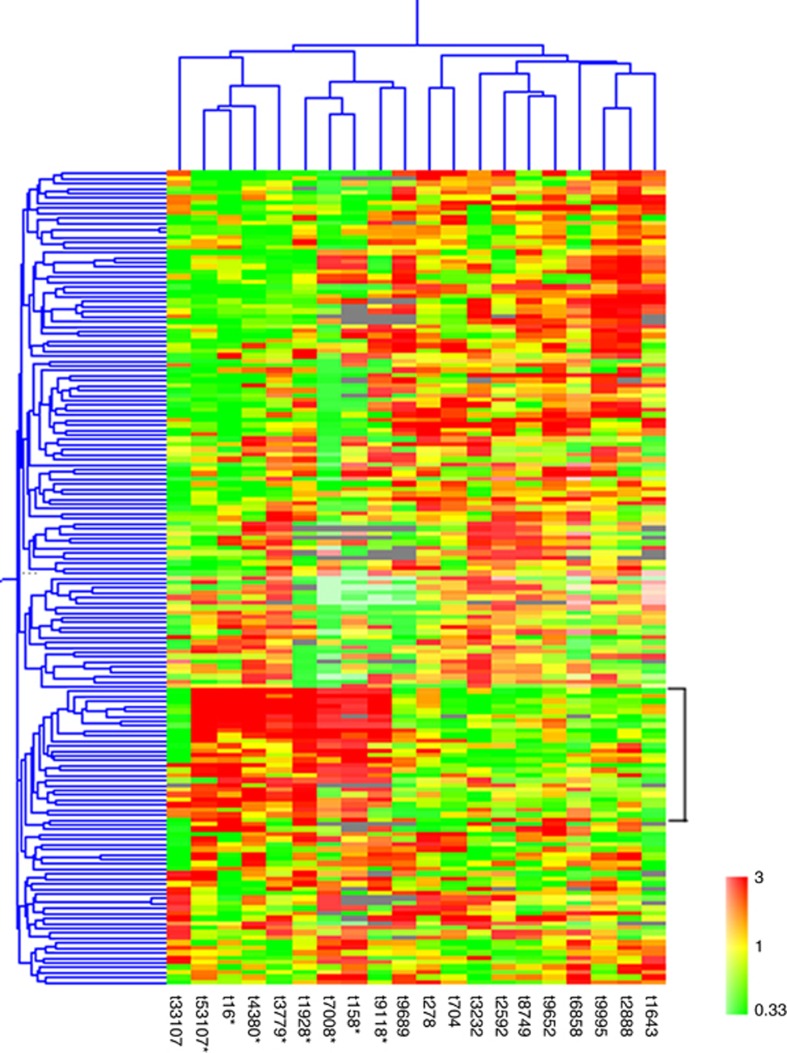
**Two-dimensional cluster analysis of leiomyosarcomas (horizontal) and most varied genes (vertical).** Each column corresponds to a tumour and each row corresponds to a gene. Red indicates overexpression, whereas green indicates underexpression. Grey indicates missing or excluded data. Asterisk indicates the tumour belongs to group I in clustering analysis of Figure 2.

**Figure 2:**
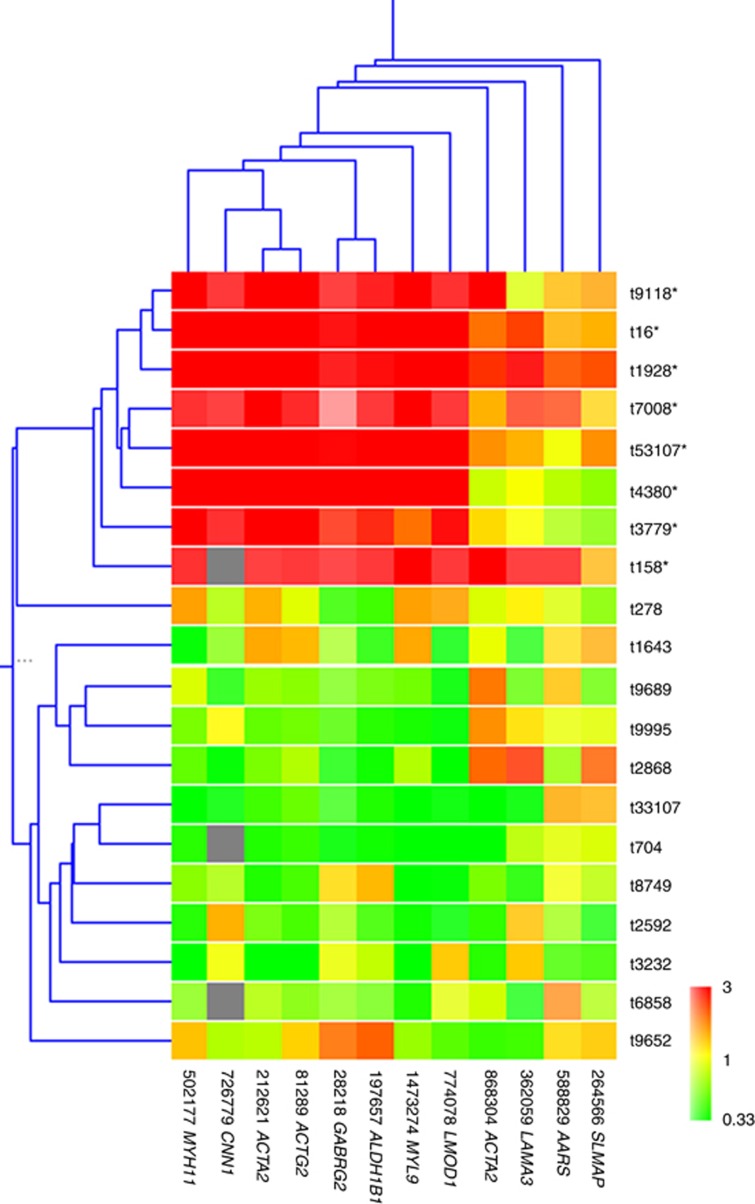
**Hierarchical clustering analyses of leiomyosarcomas (vertical) and the ‘leio-subclass' genes in our array (horizontal).** Each row corresponds to a tumour and each column corresponds to a gene. Red indicates overexpression, whereas green indicates underexpression. Grey indicates missing or excluded data. Asterisk indicates the tumour belongs to group I in this clustering analysis.

The expression profiles for 20 leiomyosarcomas were investigated using cDNA microarrays. To better explore the expression differences that may exist between the different tumours, a subset of 169 genes that showed the most variation in expression among the tumours was used in the cluster analysis. Hierarchical clustering using these most varied genes in this set of tumours gave rise to two clusters of tumours (Figure 1), one group (group I) was characterised by higher expression of a cluster of 26 genes. Ten of these genes were also found in the 20 distinct genes reported by Nielsen *et al* (2002). In making this array, we have mined the expression data from Nielsen *et al* (2002) and have specifically included clones that corresponded to all except 2 of the 20 distinct genes from Nielsen *et al* (2002) (‘leio-subclass' genes) that distinguished their leiomyosarcoma samples into the two subgroups that they called the ‘Calponin' subgroups (Supplementary Table S1). This allowed us to specifically carry out clustering analysis using the expression of these ‘leio-subclass' genes (23 gene probes corresponding to 18 of the distinct genes reported by Nielsen *et al* (2002)). Hierarchical clustering analysis on the tumours, using these 23 ‘leio-subclass' genes representing 18 distinct genes gave rise to two groups of tumours, with one group of tumours exhibiting high level of expression of these genes (called ‘Calponin-positive' group by Nielsen *et al* (2002); Figure 2). Gene clustering was also carried out for the 12 of these genes that satisfied the criteria for use in gene clustering (Figure 2).

**Figure 3:**
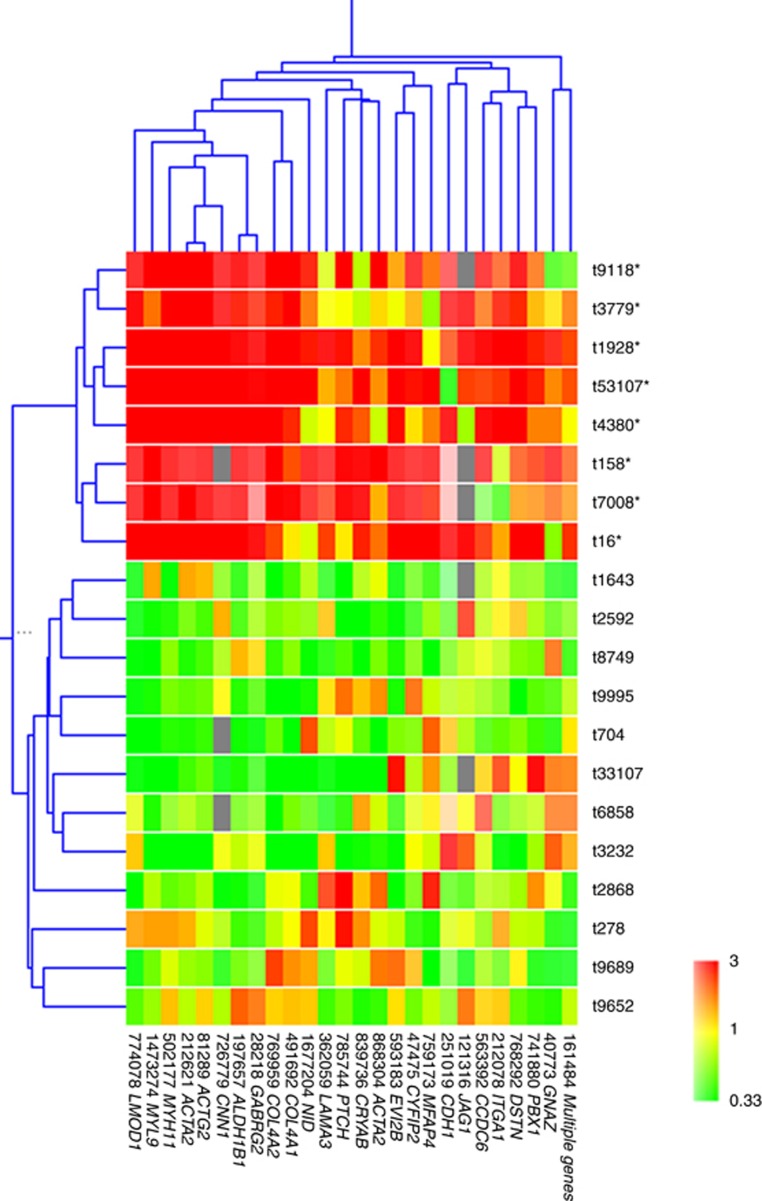
**Hierarchical clustering analyses of leiomyosarcomas (vertical) and the 26 genes (horizontal).** Each row corresponds to a tumour and each column corresponds to a gene. Red indicates overexpression, whereas green indicates underexpression. Grey indicates missing or excluded data. Asterisk indicates the tumour belongs to group I in clustering analysis of Figure 2.

We repeated tumour clustering analysis, using only the cluster of 26 genes that defined the groupings in the ‘most varied genes' analysis, and the result showed two distinct clusters of tumours (Figure 3), with a well-defined cluster of eight tumours (group I) that matched exactly with the tumours identified to be ‘Calponin positive' using the ‘leio-subclass' genes.

The group I tumours were defined by the high expression of this cluster of 26 genes (Table 1). We noted that 10 of the ‘leio-subclass' genes were present in the most varied genes (169 genes), and remarkably, all 10 of these 10 ‘leio-subclass' genes were found in this cluster of 26 genes. The 16 additional genes that were new to the ‘leio-subclass' gene cluster were also shown in Table 1, and included a number of genes that have structural importance for plasma membrane (*EVI2B*), basement membrane (*COL4A2*; *COL4A1* and *NID1*) and in extracellular matrix (*MFAP4*); cell adhesion (*CDH1; ITGA1*); as well as genes that have important functions like cell signalling (*JAG1* involved in Notch signalling; *GNAZ* in transmembrane signalling; and *PTCH1* in hedgehog and other signalling).

We then looked into the clinical relevance of these subgroups of tumours. Analysis of relevant clinical data showed a statistically significant association of the subgroupings with tumour grade (Kendall's tau_b_ 0.650, *P*<0.01; Table 2). Group I was found to be associated with the lower tumour grade. We have not found any statistically significant association of the cluster grouping with other clinical parameters (tumour size (⩽5 cm or >5 cm), site (superficial or deep), location (extremity or non-extremity), stage (I–IV) or tumour status (primary, local recurrence or metastasis); Supplementary Table S2).

We noted that a cluster of 15 genes were best associated with the subgroupings of tumours and may allow us to further refine the gene list, so these were tested to see if they could recapitulate the subgroupings of tumours (Table 1). We performed clustering analysis with this group of 15 genes and found that the tumours were clustered in the same two groups as with the use of the larger gene clusters; one group was defined by the high expression of these genes (Supplementary Figure S1). We further validated the use of this group of 15 genes in an independent sarcoma set that has not been used for identifying subgroups. We downloaded and analysed the gene expression data of the 17 leiomyosarcomas in the sarcoma study by Baird *et al* (2005). Thirteen of the genes in their data set were mapped to genes found in our group of 15 genes and clustering analysis was performed accordingly. We found that these genes also defined a subgroup of tumours (10) that had high expression of these genes.

There was also interest in identifying potential targets for therapy associated with the two subgroups, particularly as they were found to be clinically relevant. Class comparison analysis using two sample *t*-test and multivariate permutations test was performed to identify statistically significant genes associated with our two subgroups. The statistically significant genes were compared with the genes from the TARGET V3 database (Allen *et al*, 2014) that contains over 130 genes at present, which are believed to have therapeutic, prognostic and diagnostic implications for cancer patients, and are of particular interest in translational oncology. Our comparison showed that two of the genes (CDH1 and PDGFRA) in our gene list are found in these TARGET genes and may be potential subgroup-specific targets. The high expression of CDH1 is associated with group I tumours. This gene has been reported to be of diagnostic value and is frequently mutated in lobular breast carcinoma and in certain hereditary cancer syndromes, including a type of gastric cancer (Allen *et al*, 2014). The high expression of PDGFRA is associated with group II tumours, and is associated with the sensitivity to tyrosine kinase inhibitors (Allen *et al*, 2014).

## Discussion

Gene expression profiling has become an increasingly important aid in the molecular diagnosis and classification of human malignancies, including soft tissue sarcomas (Golub *et al*, 1999; Alizadeh *et al*, 2000; Bittner *et al*, 2000; Perou *et al*, 2000; Nielsen *et al*, 2002; Lee *et al*, 2003; Francis *et al*, 2007; Tschoep *et al*, 2007; Elias *et al*, 2015). Although the existing classification permits soft tissue tumours to be distinguished from one another, there is still much room for improvement in the classification scheme. Identification of new molecular subtypes will aid in the management of the disease, which was well illustrated by the introduction of the diagnostic category GIST and the development of new targeted therapies specific to this class of tumours that have revolutionised its treatment (Nishida *et al*, 2015). In this study, we have attempted to identify molecular subtypes of leiomyosarcoma, using an array designed to allow one to further investigate the subgroupings proposed by Nielsen *et al* (2002). We have focused on leiomyosarcomas of extra-uterine origins, as uterine leiomyosarcoma represents a relatively distinct clinical entity and can be easily identified from its site of origin (Amant *et al*, 2009; Linch *et al*, 2014). When clustering analysis was performed in our tumour set using the ‘leio-subclass' genes reported by Nielsen *et al* (2002), we similarly found two subgroups of leiomyosarcomas. In addition, tumour clustering using a set of most varied genes (169 genes) in our data set also gave rise to two groups of tumours that very much matched the tumour groupings using the 18 ‘leio-subclass' gene set. These groups of tumours were defined by the high expression of a cluster of 26 genes (Table 1). Sixteen additional genes were new to the ‘leio-subclass' gene cluster and it is interesting to note that a number of these genes have structural importance just like genes reported in Nielsen *et al* (2002) being implicated in muscle structure and function that included Calponin, laminin, actin and leiomodin. Also included in these 16 additional genes was the gene for D10S170 DNA fragment (clone 563392, now called CCDC6 coiled-coil domain containing 6), which was present immediately next to the ‘leio-subclass' genes in the gene dendrogram from Nielsen *et al* (2002) and also showed similar expression pattern to these genes. Though its gene function is still largely unknown, it has been suggested to have tumour suppressor function. It is worth noting that COL4A1 and COL4A2 are two closely related genes located on the same chromosome. COL4A2 gene is organised in a head-to-head conformation with the other type IV collagen gene COL4A1, so that the gene pair shares a common promoter. This supported the reliability of the microarray data, as both genes were picked up and were clustered next to each other in clustering analysis.

The tumour groupings using the most varied genes largely matched with the groupings using the ‘leio-subclass' genes, but there was a small difference in the groupings that could have arisen from the noise in the data. We might have included many genes in the clustering using the most varied genes that are not relevant to the ‘Calponin' groupings. So clustering analysis was performed using the cluster of 26 genes that defined the tumour subgroups, and we obtained two groups that matched exactly with the two groups from clustering using the ‘leio-subclass' genes.

Nielsen *et al* (2002) reported that too few cases were available to allow for meaningful comparison based on the histological findings between the two groups. And within the limit of their study, clinical features, such as tumour location, did not account for the separation of the leiomyosarcomas into the two subgroups. They did not comment directly on other clinical associations apart from the tumour location. We have investigated possible association with various clinical parameters, and we have found a statistically significant association of the subgrouping of leiomyosarcomas with the tumour grade. The histological grading of soft tissue sarcomas is determined by an assessment of various factors, including differentiation (pleomorphism of tumour cells), mitotic activity, degree of cellularity, matrix formation and amount of necrosis (Trojani *et al*, 1984; Oliveira and Nascimento, 2001). It is considered an important prognostic factor for adult soft tissue sarcomas and has been used in guiding patient management (Oliveira and Nascimento, 2001). However, a consistent reproducible grading system can at times be difficult to achieve (Oliveira and Nascimento, 2001). Genes identified from these gene expression studies may aid in achieving better tumour grading, and may be particularly useful in difficult cases.

We have further refined the gene list to a group of 15 genes that could recapitulate the tumour subgroupings in our data set, and have validated this gene list in an independent sarcoma set from Baird *et al* (2005), where we also found a subgroup of tumours defined by this group of genes. Unfortunately, associated clinical data were not available and one could not assess the association of tumour subgrouping with the tumour grade in this data set.

Guo *et al* (2015) have recently used gene expression profiling to identify three molecular subtypes of leiomyosarcoma using formalin-fixed, paraffin-embedded tumour samples () following their earlier study (Beck *et al*, 2010): subtype I was the subgroup that was relatively well-defined molecularly and had higher expression of genes enriched for processes that included muscle contraction, muscle system processes and cytoskeleton organisation. Subtype II had less muscle-specific gene expression than subtype I. Guo *et al* have proposed the use of LMOD1 (replacing CASQ2 proposed in their earlier study) together with the proteins ACTG2, SLMAP, MYLK and CFL2 to be the panel of subtype I immunohistochemical biomarkers. They have also proposed the use of ARL4C to be the subtype II biomarker. They have not proposed any immunohistochemical markers for subtype III. Subtype III appeared to be less well-defined molecularly and when they attempted to investigate if they could find these three subtypes in another publicly available gene expression data set, they noted that their subtypes I and II could be significantly reproduced in this data set, but not subtype III. However, they noted from related clinical information that clinically subtype III tumours were mostly of uterine origin (92% in their own tumour set) and uterine leiomysarcoma was significantly associated with subtype III. In our study, we have found two distinct molecular subgroups of leiomyosarcomas. As we did not include tumours of uterine origin in our study, it is expected that we would not find a subgroup that corresponded to their subtype III. Our group I was defined by a cluster of 26 genes (Table 1) that included many genes implicated in muscle structure and function. We noted that *LMOD1* and *ACTG2* from the panel of five genes whose encoded proteins they proposed to be used as immunohistochemical markers for subtype I were also found in our 26 group I defining gene list. In fact, further investigation showed that the majority of the genes in our group I defining gene list were ranked highly in their list of genes that had a higher expression in subtype I *vs* other subtypes, including *LMOD1, MYL9, MYH11, ACTA2, ACTG2, CNN1, ALDH1B1, COL4A2, COL4A1, CRYAB, CYFIP2, MFAP4, CDH1, JAG1, DSTN* and *GNAZ.* They have proposed the use of ARL4C as immunohistochemical marker for subtype II, but this gene was not included in our array. On the other hand, a cluster of four genes that correlated with and had higher expression in our group II (*GJA1, COL5A2, THBS2* and *MFAP2*) were also found to be highly ranked in their list of significant genes that had a higher expression in subtype II *vs* other subtypes. In fact, the first three of these genes were ranked higher (more significantly associated with subtype II) than the *ARL4C*, whose protein they had chosen to be the immunohistochemical marker for subtype II in their study. It is perhaps worth noting that the use of a single-protein ARL4C to be subtype II marker and its use in classifying tumours as subtype II or not, using immunohistochemistry had quite a significant error rate (31% of their tumour samples were misclassified into a different subtype than the original classification based on gene expression profiles), so perhaps a combination of markers would help to improve the accuracy of an immunohistochemical classification. Class comparison analysis was also performed to identify statistically significant genes associated with our two subgroups, we got similar genes to the clusters of group-defining genes mentioned above that also corresponded to their significant genes for subtype I and II, respectively. On the whole, our findings suggested that our group I corresponded to their subtype I; and our group II corresponded to their subtype II; providing further support to the existence of these two molecular subgroups (in a UK data set). Though they have found that low-grade tumours were more frequent in their subtype I, this did not reach statistical significance in their data set; whereas in our data set, we have found a statistically significant association of the subgroups with tumour grade, with group I being associated with lower grade.

Chibon *et al* (2010) has reported on identifying a gene expression signature, which they have called CINSARC—Complexity INdex in SARComa, that was found to be associated with metastatic outcome in a mixture of different soft tissue sarcomas. The CINSARC gene set is composed of 67 genes related to mitosis and chromosomal stability. We have compared the CINSARC genes with the statistically significant genes associated with our two subgroups and found no overlap in these genes. It is perhaps not too surprising, as although the authors found that the CINSARC genes could predict metastatic behaviour in soft tissue sarcomas in general, the authors had also done a survival analysis in their validation set of leiomysarcomas and reported that they could not find a statistically significant difference in metastasis-free survival in their two subgroups (Chibon *et al*, 2010).

To conclude, we have identified two molecular subgroups of leiomyosarcomas, one group being characterised by the high level of expression of a cluster of 26 genes that included many genes in the ‘leio-subclass' genes, and provided confirmation of the findings of Nielsen *et al* (2002) in an independent sarcoma set. We have also identified additional genes to the original ‘leio-subclass' gene cluster that contributed to sub-classification of leiomyosarcoma. We have also investigated the clinical significance of the groupings and found an association with tumour grade. We have further refined our gene list to a group of 15 genes that could recapitulate the tumour subgroupings in our data set, and have validated this in an independent sarcoma set from Baird *et al* (2005). Further comparison with a recently published data set suggested that our findings also matched with two molecular subgroups in their data set (Guo *et al*, 2015). Cross-study comparisons and analyses of expression data sets were renowned for often giving rise to discrepant or completely different results. Quite remarkably, our findings suggested that the two distinct molecular subgroups identified here could be found across four independent data sets, providing strong support for their existence. Our findings will aid in achieving a better classification of leiomyosarcomas and eventually lead to a better management of the disease and more individualised therapies for sarcoma patients.

## Figures and Tables

**Table 1:** List of genes in the cluster of 26 genes that defined the leiomyosarcoma subgrouping

**Spot ID**	**I.M.A.G.E. clone ID**	**UniGene Cluster**	**Symbol**	**Name**
774078	774078	Hs.5*19075*	*LMOD1*	Leiomodin 1 (smooth muscle)
1473274	1473274	Hs.504687	*MYL9*	Elongation factor Tu family protein
502177	502177	Hs.460109	*MYH11*	Myosin, heavy polypeptide 11, smooth muscle
212621	212621	Hs.500483	*ACTA2*	Actin, alpha 2, smooth muscle, aorta
81289	81289	Hs.516105	*ACTG2*	Actin, gamma 2, smooth muscle, enteric
726779	726779	Hs.465929	*CNN1*	Calponin 1, basic, smooth muscle
197657	197657	Hs.436219	*ALDH1B1*	Aldehyde dehydrogenase 1 family, member B1
28218	28218	Hs.7195	*GABRG2*	Gamma-aminobutyric acid A receptor, gamma 2
769959	769959^a^	Hs.508716	*COL4A2*	Collagen, type IV, alpha 2
491692	491692^a^	Hs.17441	*COL4A1*	Collagen, type IV, alpha 1
1677204	1677204^a^	Hs.356624	*NID*	Nidogen 1
362059	362059	Hs.436367	*LAMA3*	Laminin, alpha 3
785744^b^	785744^a^	Hs.494538	*PTCH*	Patched homologue (Drosophila)
839736	839736^a^	Hs.53454	*CRYAB*	Crystallin, alpha B
868304	868304	Hs.500483	*ACTA2*	Actin, alpha 2, smooth muscle, aorta
593183	593183^a^	Hs.5509	*EVI2B*	Ecotropic viral integration site 2B
47475	47475^a^	Hs.519702	*CYFIP2*	Cytoplasmic FMR1 interacting protein 2
759173^b^	759173^a^	Hs.296049	*MFAP4*	Microfibrillar-associated protein 4
251019	251019^a^	Hs.461086	*CDH1*	Cadherin 1, type 1, E-cadherin (epithelial)
121316^b^	121316^a^	Hs.224012	*JAG1*	Jagged 1 (Alagille syndrome)
563392	563392^a^	Hs.591360	*CCDC6*	Coiled-coil domain containing 6
212078^b^	212078^a^	Hs.644352	*ITGA1*	Integrin, alpha 1
768292^b^	768292^a^	Hs.304192	*DSTN*	Destrin (actin depolymerising factor)
741880	741880^a^	Hs.557097	*PBX1*	Pre-B-cell leukaemia transcription factor 1
40773	40773^a^	Hs.584760	*GNAZ*	Guanine nucleotide binding protein (G protein), alpha z polypeptide
161484^b^	161484^a^	—	—	Multiple genes

NB: the refined group of 15 genes were shown as the top 15 genes in this table.

a Genes that were new to the ‘leio-subclass' genes.

b Clones that were found by DNA sequencing to have a sequence different from the one originally associated with the I.M.A.G.E. clone ID during sequence confirmation of the genes.

**Table 2:** Distribution of tumour grade *vs* tumour subgroup

	**Tumour subgroup**		
	**Group I**	**Group II**	**Total**
**Tumour grade**			
1	1	0	1
2	5	1	6
3	2	9	11
Total	8	10	18
