# Supplementary material for: Gene expression profiling identifies distinct molecular subgroups of leiomyosarcoma with clinical relevance
Source: Br J Cancer. 2016 Sep 8;115(8):1000–7. doi: 10.1038/bjc.2016.280 (PMC5061910; doi:10.1038/bjc.2016.280)
Supplement: Supplementary Figure 1 [file bjc2016280x1.pdf]

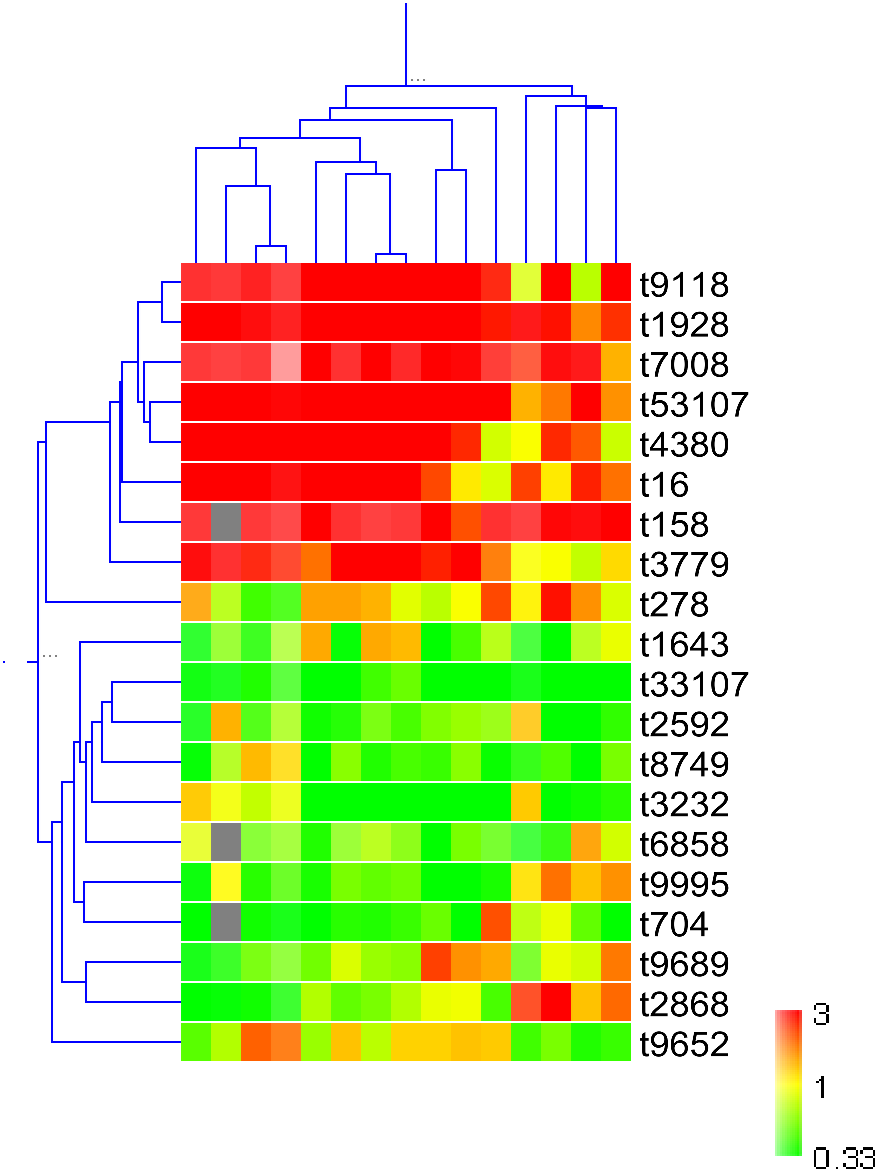

Supplementary Fig. S1 Hierarchical clustering analyses of leiomyosarcomas (vertical) and the refined group of 15 genes (horizontal). Each row corresponds to a tumour, and each column corresponds to a gene. Red indicates overexpression, while green indicates underexpression. Grey indicates missing or excluded data.
