# Supplementary material for: Gene expression profiling identifies distinct molecular subgroups of leiomyosarcoma with clinical relevance
Source: Br J Cancer. 2016 Sep 8;115(8):1000–7. doi: 10.1038/bjc.2016.280 (PMC5061910; doi:10.1038/bjc.2016.280)
Supplement: Supplementary Material [file bjc2016280x2.pdf]

Description of supplementary data files

Supplementary Fig. S1 Hierarchical clustering analyses of leiomyosarcomas (vertical) and the refined group of 15 genes (horizontal).

Supplementary Table S1 Array geneset information.

Supplementary Table S2 Clinical data table.
