# Supplementary material for: Gene expression profiling identifies distinct molecular subgroups of leiomyosarcoma with clinical relevance
Source: Br J Cancer. 2016 Sep 8;115(8):1000–7. doi: 10.1038/bjc.2016.280 (PMC5061910; doi:10.1038/bjc.2016.280)
Supplement: Supplementary Table 1 [file bjc2016280x3.pdf]

Supplementary Table S1

Array geneset information

| CloneID | UniGene Cluster | Symbol | Name |
|---|---|---|---|
| 207618 | Hs.446641 | ARAF | V-raf murine sarcoma 3611 viral oncogene homolog |
| 782513 | Hs.523847 | IFI6 | Interferon, alpha-inducible protein 6 |
| 233581 | Hs.50308 | UBE2K | Ubiquitin-conjugating enzyme E2K |
| 62277 | Data not found | | |
| 785845 | Hs.594250 | TFAM | Transcription factor A, mitochondrial |
| 758222 | Hs.477 | HSD17B3 | Hydroxysteroid (17-beta) dehydrogenase 3 |
| 239877 | Hs.519632 | HDAC3 | Histone deacetylase 3 |
| 294487 | In multiple ClusterIDs | | |
| 712641 | Hs.279640 | TPR | Translocated promoter region, nuclear basket protein |
| 156386 | Hs.514038 | FLOT2 | Flotillin 2 |
| 544639 | Hs.95612 | DSC2 | Desmocollin 2 |
| 52629 | Hs.434875 | CAMK1 | Calcium/calmodulin-dependent protein kinase I |
| 796258 | Hs.463412 | SGCA | Sarcoglycan, alpha (50kDa dystrophin-associated glycoprotein) |
| 145681* | Hs.519075 | LMOD1 | Leiomodin 1 (smooth muscle) |
| 563392 | Hs.591360 | CCDC6 | Coiled-coil domain containing 6 |
| 376206* | Hs.655519 | SYNPO2 | Synaptopodin 2 |
| 122394 | Hs.73797 | GNA15 | Guanine nucleotide binding protein (G protein), alpha 15 (Gq class) |
| 868400 | Hs.79322 | QARS | Glutaminyl-tRNA synthetase |
| 288695 | Hs.129261 | SS18 | Synovial sarcoma translocation, chromosome 18 |
| 490819 | Hs.151641 | LRRC32 | Leucine rich repeat containing 32 |
| 487071 | Hs.82963 | GNRH1 | Gonadotropin-releasing hormone 1 (luteinizing-releasing hormone) |
| 856447 | Hs.14623 | IFI30 | Interferon, gamma-inducible protein 30 |
| 840486 | Hs.440848 | VWF | Von Willebrand factor |
| 773305 | Hs.504352 | NTM | Neurotrimin |
| 155575 | Hs.437422 | EPB41L1 | Erythrocyte membrane protein band 4.1-like 1 |
| 740914 | Hs.208597 | CTBP1 | C-terminal binding protein 1 |
| 343646 | In multiple ClusterIDs | | |
| 297392 | Hs.374950 | MT1X | Metallothionein 1X |
| 772111 | Hs.481819 | PDZD2 | PDZ domain containing 2 |

| | | | |
|---|---|---|---|
| 789049 | In multiple ClusterIDs | | |
| 322617 | Hs.469820 | RALB | V-ral simian leukemia viral oncogene homolog B (ras related; GTP binding protein) |
| 789069 | In multiple ClusterIDs | | |
| 785963 | Hs.655407 | ZCCHC11 | Zinc finger, CCHC domain containing 11 |
| 207358 | Hs.473721 | SLC2A1 | Solute carrier family 2 (facilitated glucose transporter), member 1 |
| 796278 | Hs.355348 | GTF2H3 | General transcription factor IIH, polypeptide 3, 34kDa |
| 971382 | Hs.292265 | ZMYND11 | Zinc finger, MYND-type containing 11 |
| 725176 | Hs.520037 | NEU1 | Sialidase 1 (lysosomal sialidase) |
| 855586 | Hs.703520 | | Transcribed locus |
| 435036 | Hs.3416 | PLIN2 | Perilipin 2 |
| 502622 | Hs.387567 | ACLY | ATP citrate lyase |
| 364934 | Hs.380277 | DAPK1 | Death-associated protein kinase 1 |
| 469306 | Hs.153444 | GRP | Gastrin-releasing peptide |
| 725501 | Hs.654596 | ZBTB6 | Zinc finger and BTB domain containing 6 |
| 878449 | Hs.335079 | MAP1B | Microtubule-associated protein 1B |
| 490805 | Hs.643658 | NCOA4 | Nuclear receptor coactivator 4 |
| 323238 | Hs.789 | CXCL1 | Chemokine (C-X-C motif) ligand 1 (melanoma growth stimulating activity, alpha) |
| 200018 | Hs.2484 | TCL1A | T-cell leukemia/lymphoma 1A |
| 845663 | Hs.471818 | CAPRIN1 | Cell cycle associated protein 1 |
| 361807 | Hs.517978 | GNAT1 | Guanine nucleotide binding protein (G protein), alpha transducing activity polypeptide 1 |
| 454908 | Hs.119689 | CGA | Glycoprotein hormones, alpha polypeptide |
| 852913 | Hs.105465 | SNRPF | Small nuclear ribonucleoprotein polypeptide F |
| 429368 | Hs.89583 | TLX1 | T-cell leukemia homeobox 1 |
| 811740 | Hs.482077 | ITGA2 | Integrin, alpha 2 (CD49B, alpha 2 subunit of VLA-2 receptor) |
| 1089539 | Hs.654380 | KRT14 | Keratin 14 |
| 594502 | Hs.244139 | FAS | Fas (TNF receptor superfamily, member 6) |
| 759142 | Hs.470399 | ITGB6 | Integrin, beta 6 |
| 842884 | Hs.115474 | RFC3 | Replication factor C (activator 1) 3, 38kDa |
| 1269337 | Hs.109731 | GLG1 | Golgi glycoprotein 1 |
| 625584 | Hs.517972 | TRAIP | TRAF interacting protein |
| 767055 | Hs.252451 | SEMA3A | Sema domain, immunoglobulin domain (Ig), short basic |

| | | | |
|---|---|---|---|
| | | | domain, secreted, (semaphorin) 3A |
| 843170 | Hs.73853 | BMP2 | Bone morphogenetic protein 2 |
| 1472775 | Hs.654548 | COL8A1 | Collagen, type VIII, alpha 1 |
| 646654 | Hs.191346 | Sep-07 | Septin 7 |
| 769716 | Hs.187898 | NF2 | Neurofibromin 2 (merlin) |
| 845363 | Hs.463456 | NME2 | NME/NM23 nucleoside diphosphate kinase 2 |
| 1520872 | Hs.657729 | LRP2 | Low density lipoprotein receptor-related protein 2 |
| 665726 | In multiple ClusterIDs | | |
| 772057 | In multiple ClusterIDs | | |
| 868212 | Hs.369397 | TGFBI | Transforming growth factor, beta-induced, 68kDa |
| 1523210 | Hs.487360 | NOTCH2 | Notch 2 |
| 725308 | Hs.1030 | RIN1 | Ras and Rab interactor 1 |
| 415388 | Hs.18573 | ACYP1 | Acylphosphatase 1, erythrocyte (common) type |
| 743230 | Hs.523789 | NEAT1 | Nuclear paraspeckle assembly transcript 1 (non-protein coding) |
| 345833 | Hs.591731 | HNRNPAB | Heterogeneous nuclear ribonucleoprotein A/B |
| 41406 | Hs.533336 | BAMBI | BMP and activin membrane-bound inhibitor homolog (Xenopus laevis) |
| 82225 | Hs.213424 | SFRP1 | Secreted frizzled-related protein 1 |
| 68605 | Hs.479808 | IGFBP7 | Insulin-like growth factor binding protein 7 |
| 625584 | Hs.517972 | TRAIP | TRAF interacting protein |
| 141731 | Hs.592095 | SLC16A5 | Solute carrier family 16, member 5 (monocarboxylic acid transporter 6) |
| 81475 | Hs.8546 | NOTCH3 | Notch 3 |
| 624360 | Hs.180062 | PSMB8 | Proteasome (prosome, macropain) subunit, beta type, 8 (large multifunctional peptidase 7) |
| 838434 | Hs.72910 | CRYGC | Crystallin, gamma C |
| 590544 | In multiple ClusterIDs | | |
| 755402 | Hs.265829 | ITGA3 | Integrin, alpha 3 (antigen CD49C, alpha 3 subunit of VLA-3 receptor) |
| 811015 | Hs.25647 | FOS | FBJ murine osteosarcoma viral oncogene homolog |
| 1057458 | Hs.485233 | MAPK14 | Mitogen-activated protein kinase 14 |
| 592125 | Hs.519842 | RIPK1 | Receptor (TNFRSF)-interacting serine-threonine kinase 1 |
| 756465 | Hs.591033 | MMP19 | Matrix metallopeptidase 19 |
| 207794 | Hs.75643 | NFE2 | Nuclear factor (erythroid-derived 2), 45kDa |
| 293916 | Hs.597209 | | Transcribed locus, strongly similar to XP_003892248.1 |

| | | | |
|---|---|---|---|
| | | | PREDICTED: glomulin-like [Papio anubis] |
| 299679 | Hs.536913 | | PA9=candidate tumor suppressor gene {3' region} [human, HEN endocervical cell line, mRNA Partial, 292 nt] |
| 144932 | Hs.433201 | CDK2AP1 | Cyclin-dependent kinase 2 associated protein 1 |
| 245136 | Hs.16349 | ATMIN | ATM interactor |
| 138991 | Hs.233240 | COL6A3 | Collagen, type VI, alpha 3 |
| 21899 | Hs.285197 | SRPK2 | SRSF protein kinase 2 |
| 491544 | In multiple ClusterIDs | | |
| 40608 | Hs.388226 | HCRTR1 | Hypocretin (orexin) receptor 1 |
| 741831 | Hs.439312 | PLTP | Phospholipid transfer protein |
| 435858 | Hs.23262 | RNASE6 | Ribonuclease, RNase A family, k6 |
| 531957 | In multiple ClusterIDs | | |
| 85320 | Hs.295923 | | Transcribed locus |
| 77244 | In multiple ClusterIDs | | |
| 744800 | Hs.19718 | PTPRU | Protein tyrosine phosphatase, receptor type, U |
| 79592 | Hs.571886 | AKR7A2 | Aldo-keto reductase family 7, member A2 (aflatoxin aldehyde reductase) |
| 49303 | Hs.655213 | PPP2R2B | Protein phosphatase 2, regulatory subunit B, beta |
| 609087 | Hs.349656 | SCARB2 | Scavenger receptor class B, member 2 |
| 244703 | Hs.696554 | ITGBL1 | Integrin, beta-like 1 (with EGF-like repeat domains) |
| 207288 | Hs.520819 | INSIG1 | Insulin induced gene 1 |
| 283301 | Hs.97270 | FAM13A | Family with sequence similarity 13, member A |
| 139573 | In multiple ClusterIDs | | |
| 166004 | Hs.466766 | LTBP4 | Latent transforming growth factor beta binding protein 4 |
| 47475 | Hs.519702 | CYFIP2 | Cytoplasmic FMR1 interacting protein 2 |
| 43207 | Hs.441202 | | GFRA2 |
| 243580 | Hs.220864 | CHD2 | Chromodomain helicase DNA binding protein 2 |
| 770192 | Hs.81337 | LGALS9 | Lectin, galactoside-binding, soluble, 9 |
| 243358 | Hs.46700 | ING1 | Inhibitor of growth family, member 1 |
| 755416 | Hs.167700 | SMAD5 | SMAD family member 5 |
| 130820 | Hs.443881 | PAXIP1 | PAX interacting (with transcription-activation domain) protein 1 |
| 129865 | Hs.250822 | AURKA | Aurora kinase A |
| 33478 | Hs.335084 | FPGS | Folylpolyglutamate synthase |

| | | | |
|---|---|---|---|
| 347373 | In multiple ClusterIDs | | |
| 340558 | Hs.518609 | ARPC5 | Actin related protein 2/3 complex, subunit 5, 16kDa |
| 206994 | Hs.478125 | INADL | InaD-like (Drosophila) |
| 770910 | Hs.603657 | ELF3 | E74-like factor 3 (ets domain transcription factor, epithelial-specific ) |
| 824081 | Hs.490765 | KCNN3 | Potassium intermediate/small conductance calcium-activated channel, subfamily N, member 3 |
| 725076 | Hs.97439 | NT5C2 | 5'-nucleotidase, cytosolic II |
| 511303 | Hs.410477 | PLA2R1 | Phospholipase A2 receptor 1, 180kDa |
| 222107 | Hs.479756 | KDR | Kinase insert domain receptor (a type III receptor tyrosine kinase) |
| 825648 | Hs.81221 | IGHV5-78 | Immunoglobulin heavy variable 5-78 (pseudogene) |
| 1472643 | Hs.370504 | RPS15A | Ribosomal protein S15a |
| 681997 | Hs.591167 | DLX4 | Distal-less homeobox 4 |
| 754736 | Hs.436873 | ITGAV | Integrin, alpha V |
| 430614 | Hs.198365 | BPGM | 2,3-bisphosphoglycerate mutase |
| 896949 | Hs.643495 | | Transcribed locus |
| 1358393 | Hs.514012 | MAP2K3 | Mitogen-activated protein kinase kinase 3 |
| 2465945 | Hs.446376 | APC2 | Adenomatosis polyposis coli 2 |
| 502177* | Hs.460109 | MYH11 | Myosin, heavy chain 11, smooth muscle |
| 810272* | Hs.444403 | PPP1R12B | Protein phosphatase 1, regulatory subunit 12B |
| 588829* | Hs.315137 | AARS | Alanyl-tRNA synthetase |
| 28218* | Hs.7195 | GABRG2 | Gamma-aminobutyric acid (GABA) A receptor, gamma 2 |
| 241489 | In multiple ClusterIDs | | |
| 856650 | Hs.418668 | ATP5D | ATP synthase, H+ transporting, mitochondrial F1 complex, delta subunit |
| 856535 | Hs.652308 | MTHFD1 | Methylenetetrahydrofolate dehydrogenase (NADP+ dependent) 1, methenyltetrahydrofolate cyclohydrolase, formyltetrahydrofolate synthetase |
| 432210 | Hs.2246 | CPN1 | Carboxypeptidase N, polypeptide 1 |
| 377252 | Hs.167046 | ADORA2B | Adenosine A2b receptor |
| 809876 | Hs.524464 | ATP5G2 | ATP synthase, H+ transporting, mitochondrial Fo complex, subunit C2 (subunit 9) |
| 814780 | Hs.591269 | CAMK4 | Calcium/calmodulin-dependent protein kinase IV |
| 505881 | Hs.654536 | ADA | Adenosine deaminase |

| | | | |
|---|---|---|---|
| 502055 | Hs.149103 | ARSB | Arylsulfatase B |
| 300367 | Hs.41565 | MSTN | Myostatin |
| 586650 | Hs.25450 | SLC29A1 | Solute carrier family 29 (nucleoside transporters), member 1 |
| 857681 | Hs.547172 | MAMDC2 | MAM domain containing 2 |
| 588911 | Hs.524760 | OAS1 | 2'-5'-oligoadenylate synthetase 1, 40/46kDa |
| 2066371 | Hs.260074 | CYP19A1 | Cytochrome P450, family 19, subfamily A, polypeptide 1 |
| 704690 | Hs.201918 | HIPK3 | Homeodomain interacting protein kinase 3 |
| 858293 | Hs.74405 | YWHAQ | Tyrosine 3-monooxygenase/tryptophan 5-monooxygenase activation protein, theta polypeptide |
| 246748 | Hs.159494 | BTK | Bruton agammaglobulinemia tyrosine kinase |
| 2147728 | Hs.150749 | BCL2 | B-cell CLL/lymphoma 2 |
| 789314 | Hs.1799 | CD1D | CD1d molecule |
| 741977 | Hs.69771 | CFB | Complement factor B |
| 307069 | Hs.523841 | ALDH3B1 | Aldehyde dehydrogenase 3 family, member B1 |
| 28510 | Hs.519220 | CNTN2 | Contactin 2 (axonal) |
| 229901 | Hs.75262 | CTSO | Cathepsin O |
| 755923 | Hs.31210 | BCL3 | B-cell CLL/lymphoma 3 |
| 856519 | Hs.499886 | ALDH3A2 | Aldehyde dehydrogenase 3 family, member A2 |
| 841179 | Hs.632368 | EXOSC10 | Exosome component 10 |
| 949971 | Hs.496487 | ATF4 | Activating transcription factor 4 (tax-responsive enhancer element B67) |
| 342181 | Hs.150749 | BCL2 | B-cell CLL/lymphoma 2 |
| 208542 | Hs.1219 | ADH4 | Alcohol dehydrogenase 4 (class II), pi polypeptide |
| 825170 | Hs.477155 | ATP6V1A | ATPase, H+ transporting, lysosomal 70kDa, V1 subunit A |
| 789383 | Hs.200250 | CREM | CAMP responsive element modulator |
| 206795 | In multiple ClusterIDs | | |
| 266312 | Hs.492280 | ATP7B | ATPase, Cu++ transporting, beta polypeptide |
| 486113 | In multiple ClusterIDs | | |
| 448193 | Hs.632446 | ARNT | Aryl hydrocarbon receptor nuclear translocator |
| 1011317 | Hs.194669 | EZH1 | Enhancer of zeste homolog 1 (Drosophila) |
| 344489 | Hs.203952 | TRRAP | Transformation/transcription domain-associated protein |
| 1711262 | Hs.9333 | PDE8A | Phosphodiesterase 8A |
| 800137 | Hs.155644 | PAX2 | Paired box 2 |
| 2090446 | Hs.655467 | POLH | Polymerase (DNA directed), eta |

| | | | |
|---|---|---|---|
| 2535214 | Hs.518513 | TNK2 | Tyrosine kinase, non-receptor, 2 |
| 1467706 | Hs.643130 | PAWR | PRKC, apoptosis, WT1, regulator |
| 2578771 | Hs.713764 | ACTG1 | Actin, gamma 1 |
| 712162 | Hs.146806 | CUL1 | Cullin 1 |
| 953543 | Hs.654616 | CASP6 | Caspase 6, apoptosis-related cysteine peptidase |
| 1873594 | Hs.267659 | VAV3 | Vav 3 guanine nucleotide exchange factor |
| 179667 | Data not found | | |
| 1113071 | Hs.235935 | NOV | Nephroblastoma overexpressed |
| 2357094 | Hs.291363 | CHEK2 | Checkpoint kinase 2 |
| 298625 | Hs.380271 | OGG1 | 8-oxoguanine DNA glycosylase |
| 2518510 | Hs.517517 | EP300 | E1A binding protein p300 |
| 2089793 | Hs.709201 | FGD1 | FYVE, RhoGEF and PH domain containing 1 |
| 2005569 | Hs.481022 | SFRP2 | Secreted frizzled-related protein 2 |
| 345103 | Hs.523329 | EPHB2 | EPH receptor B2 |
| 129988 | Hs.89538 | CETP | Cholesteryl ester transfer protein, plasma |
| 796388 | Hs.444329 | GPC6 | Glypican 6 |
| 1456160 | Hs.546239 | AZGP1 | Alpha-2-glycoprotein 1, zinc-binding |
| 328745 | Hs.368626 | RTN1 | Reticulon 1 |
| 809998 | Hs.655232 | | AMY1C |
| 1410444 | Hs.270833 | AREG | Amphiregulin |
| 1493527 | Hs.489207 | ASNS | Asparagine synthetase (glutamine-hydrolyzing) |
| 47481 | Hs.85201 | CLEC2B | C-type lectin domain family 2, member B |
| 815575 | Hs.153961 | ACTR1A | ARP1 actin-related protein 1 homolog A, centractin alpha (yeast) |
| 1469230 | Hs.743989 | COX8A | Cytochrome c oxidase subunit VIIIA (ubiquitous) |
| 1493383 | Hs.618145 | CIRBP | Cold inducible RNA binding protein |
| 1950962 | Hs.82071 | CITED2 | Cbp/p300-interacting transactivator, with Glu/Asp-rich carboxy-terminal domain, 2 |
| 1406602 | Hs.655801 | TNFRSF10C | Tumor necrosis factor receptor superfamily, member 10c, decoy without an intracellular domain |
| 842884 | Hs.115474 | RFC3 | Replication factor C (activator 1) 3, 38kDa |
| 2089619 | Hs.19192 | CDK2 | Cyclin-dependent kinase 2 |
| 1582627 | Hs.459211 | AKAP13 | A kinase (PRKA) anchor protein 13 |
| 325224 | Hs.653163 | TAF9 | TAF9 RNA polymerase II, TATA box binding protein (TBP)-associated factor, 32kDa |
| 230218 | Hs.94672 | BLOC1S1 | Biogenesis of lysosomal organelles complex-1, subunit 1 |

| | | | |
|---|---|---|---|
| 236355 | In multiple ClusterIDs | | |
| 300237 | In multiple ClusterIDs | | |
| 276449 | Hs.255230 | GUSB | Glucuronidase, beta |
| 214068 | Hs.524134 | GATA3 | GATA binding protein 3 |
| 306901 | In multiple ClusterIDs | | |
| 564756 | Hs.587054 | G3BP1 | GTPase activating protein (SH3 domain) binding protein 1 |
| 42864 | Hs.369089 | COL4A5 | Collagen, type IV, alpha 5 |
| 812033 | Hs.328232 | GPC1 | Glypican 1 |
| 1323157 | Hs.362324 | ZNF345 | Zinc finger protein 345 |
| 488207 | Hs.468675 | PDPN | Podoplanin |
| 41074 | Hs.10734 | CHRNA4 | Cholinergic receptor, nicotinic, alpha 4 (neuronal) |
| 767495 | Hs.21509 | GLI3 | GLI family zinc finger 3 |
| 1476181 | Hs.122511 | CETN1 | Centrin, EF-hand protein, 1 |
| 769926 | Hs.93659 | PDIA4 | Protein disulfide isomerase family A, member 4 |
| 782406 | Hs.498313 | ADSS | Adenylosuccinate synthase |
| 823574 | In multiple ClusterIDs | | |
| 1323539 | Hs.327179 | SLC17A3 | Solute carrier family 17 (sodium phosphate), member 3 |
| 160656 | Hs.625725 | BCAM | Basal cell adhesion molecule (Lutheran blood group) |
| 245920 | Hs.82614 | GYS2 | Glycogen synthase 2 (liver) |
| 756480 | Hs.532790 | NMT1 | N-myristoyltransferase 1 |
| 139009 | Hs.203717 | FN1 | Fibronectin 1 |
| 755037 | In multiple ClusterIDs | | |
| 248261 | Hs.584238 | GLDC | Glycine dehydrogenase (decarboxylating) |
| 768443 | Hs.389700 | MGST1 | Microsomal glutathione S-transferase 1 |
| 752631 | Hs.732357 | | Transcribed locus |
| 33690 | Hs.369606 | CPSF6 | Cleavage and polyadenylation specific factor 6, 68kDa |
| 811792 | Hs.82327 | GSS | Glutathione synthetase |
| 52228 | Hs.519693 | | GRIA1 |
| 360478 | Hs.483635 | FGF1 | Fibroblast growth factor 1 (acidic) |
| 509760 | In multiple ClusterIDs | | |
| 713922 | Hs.279837 | GSTM2 | Glutathione S-transferase mu 2 (muscle) |
| 173228 | Hs.151413 | GMFB | Glia maturation factor, beta |

| | | | |
|---|---|---|---|
| 767851 | Hs.591133 | FBN1 | Fibrillin 1 |
| 768299 | Hs.712948 | ZNF652 | Zinc finger protein 652 |
| 361639 | Hs.315562 | GCLM | Glutamate-cysteine ligase, modifier subunit |
| 950445 | Hs.105818 | PPP2CA | Protein phosphatase 2, catalytic subunit, alpha isozyme |
| 951233 | Hs.82793 | PSMB3 | Proteasome (prosome, macropain) subunit, beta type, 3 |
| 627542 | Hs.488143 | BLVRA | Biliverdin reductase A |
| 236305 | In multiple ClusterIDs | | |
| 128302 | Hs.504613 | PTMS | Parathymosin |
| 781050 | Hs.655327 | PFDN5 | Prefoldin subunit 5 |
| 203132 | Hs.478275 | TNFSF10 | Tumor necrosis factor (ligand) superfamily, member 10 |
| 208413 | Hs.182385 | HPN | Hepsin |
| 135083 | Hs.591095 | PDIA3 | Protein disulfide isomerase family A, member 3 |
| 83363 | Hs.279257 | PCMT1 | Protein-L-isoaspartate (D-aspartate) O-methyltransferase |
| 235155 | Hs.433300 | FCER1G | Fc fragment of IgE, high affinity I, receptor for; gamma polypeptide |
| 785293 | Hs.502775 | PLA2G16 | Phospholipase A2, group XVI |
| 212621* | Hs.500483 | ACTA2 | Actin, alpha 2, smooth muscle, aorta |
| 68828 | Hs.414795 | SERPINE1 | Serpin peptidase inhibitor, clade E (nexin, plasminogen activator inhibitor type 1), member 1 |
| 264566* | Hs.476432 | | SLMAP |
| 166245 | Hs.558334 | GRIN1 | Glutamate receptor, ionotropic, N-methyl D-aspartate 1 |
| 307660 | Hs.391561 | FABP4 | Fatty acid binding protein 4, adipocyte |
| 450307 | Hs.75841 | ERP29 | Endoplasmic reticulum protein 29 |
| 290724 | Hs.130838 | MICA | MHC class I polypeptide-related sequence A |
| 33643 | Data not found | | |
| 530359 | Hs.370312 | FNTA | Farnesyltransferase, CAAX box, alpha |
| 278808 | Hs.502511 | SPI1 | Spleen focus forming virus (SFFV) proviral integration oncogene spi1 |
| 154093 | Hs.632531 | CIR1 | Corepressor interacting with RBPJ, 1 |
| 595009 | Hs.643553 | LINC00667 | Long intergenic non-protein coding RNA 667 |
| 502369 | Hs.443831 | PDCD5 | Programmed cell death 5 |
| 712840 | Hs.595276 | STAT5B | Signal transducer and activator of transcription 5B |
| 66718 | Hs.226307 | | APOBEC3B |
| 46916 | Hs.546267 | MMP16 | Matrix metallopeptidase 16 (membrane-inserted) |
| 859359 | Hs.50649 | TP53I3 | Tumor protein p53 inducible protein 3 |
| 897596 | Hs.532359 | | RPL5 |

| | | | |
|---|---|---|---|
| 85840 | Hs.503911 | NNMT | Nicotinamide N-methyltransferase |
| 358531 | In multiple ClusterIDs | | |
| 275180 | In multiple ClusterIDs | | |
| 49351 | Hs.632839 | PLXNA3 | Plexin A3 |
| 756687 | Hs.731377 | SCARB1 | Scavenger receptor class B, member 1 |
| 743077 | Hs.98008 | GK2 | Glycerol kinase 2 |
| 724932 | Hs.235116 | GRK6 | G protein-coupled receptor kinase 6 |
| 291965 | Hs.128067 | WIPF1 | WAS/WASL interacting protein family, member 1 |
| 741885 | In multiple ClusterIDs | | |
| 263014 | Hs.268573 | GSTT1 | Glutathione S-transferase theta 1 |
| 433253 | Hs.494496 | FBP1 | Fructose-1,6-bisphosphatase 1 |
| 51826 | Hs.482043 | NNT | Nicotinamide nucleotide transhydrogenase |
| 179776 | Hs.89864 | | SKIV2L |
| 256907 | Hs.102484 | GSTA3 | Glutathione S-transferase alpha 3 |
| 502367 | Hs.24601 | FBLN1 | Fibulin 1 |
| 855890 | Hs.350265 | LONP1 | Lon peptidase 1, mitochondrial |
| 291478 | Hs.170019 | RUNX3 | Runt-related transcription factor 3 |
| 489839 | Hs.435765 | ENPEP | Glutamyl aminopeptidase (aminopeptidase A) |
| 855755 | Hs.299002 | FBL | Fibrillarin |
| 365883 | Hs.413482 | C21orf33 | Chromosome 21 open reading frame 33 |
| 773290 | Hs.662503 | | Transcribed locus |
| 899976 | Hs.405958 | CDC6 | Cell division cycle 6 |
| 1579639 | Hs.856 | IFNG | Interferon, gamma |
| 712899 | Hs.473163 | BMP7 | Bone morphogenetic protein 7 |
| 785802 | Hs.481022 | SFRP2 | Secreted frizzled-related protein 2 |
| 978715 | Hs.524390 | TUBA1B | Tubulin, alpha 1b |
| 1589786 | Hs.111 | FGF9 | Fibroblast growth factor 9 (glia-activating factor) |
| 740302 | Hs.490415 | ZYX | Zyxin |
| 796096 | Hs.461925 | RPA1 | Replication protein A1, 70kDa |
| 1011586 | Hs.440955 | ITGA4 | Integrin, alpha 4 (antigen CD49D, alpha 4 subunit of VLA-4 receptor) |
| 1616363 | Hs.503878 | NCAM1 | Neural cell adhesion molecule 1 |
| 742936 | In multiple ClusterIDs | | |

| | | | |
|---|---|---|---|
| 796989 | Hs.733399 | | Transcribed locus, strongly similar to NP_000957.1 RARG gene product [Homo sapiens] |
| 1031744 | Hs.492407 | YWHAZ | Tyrosine 3-monooxygenase/tryptophan 5-monooxygenase activation protein, zeta polypeptide |
| 1627984 | Hs.269408 | E2F3 | E2F transcription factor 3 |
| 754034 | Hs.1166 | THPO | Thrombopoietin |
| 809526 | Hs.32981 | SEMA3F | Sema domain, immunoglobulin domain (Ig), short basic domain, secreted, (semaphorin) 3F |
| 1034293 | Hs.1274 | BMP1 | Bone morphogenetic protein 1 |
| 1631867 | Hs.388739 | XRCC5 | X-ray repair complementing defective repair in Chinese hamster cells 5 (double-strand-break rejoining) |
| 285460 | Hs.390171 | COL11A2 | Collagen, type XI, alpha 2 |
| 796134 | Hs.594838 | MAP3K7 | Mitogen-activated protein kinase kinase kinase 7 |
| 594517 | In multiple ClusterIDs | | |
| 755581 | In multiple ClusterIDs | | |
| 204686 | Hs.442498 | FXYD1 | FXYD domain containing ion transport regulator 1 |
| 67759 | Hs.507658 | ALOX5AP | Arachidonate 5-lipoxygenase-activating protein |
| 877835 | Hs.182825 | RPL35 | Ribosomal protein L35 |
| 611075 | Hs.67397 | HOXA1 | Homeobox A1 |
| 769537 | Hs.196176 | ECH1 | Enoyl CoA hydratase 1, peroxisomal |
| 86160 | In multiple ClusterIDs | | |
| 511091 | Hs.646318 | | ANKRD36C |
| 302632 | Hs.155586 | LRRC23 | Leucine rich repeat containing 23 |
| 666121 | Hs.517228 | TIAM1 | T-cell lymphoma invasion and metastasis 1 |
| 774100 | Hs.522818 | L1CAM | L1 cell adhesion molecule |
| 877827 | Hs.311640 | RPS27A | Ribosomal protein S27a |
| 1555706 | Hs.514174 | JUP | Junction plakoglobin |
| 685926 | Data not found | | |
| 784589 | Hs.80343 | MMP15 | Matrix metallopeptidase 15 (membrane-inserted) |
| 127193 | Hs.407482 | MFSD2B | Major facilitator superfamily domain containing 2B |
| 150623 | Hs.137510 | | NCOR2 |
| 357344 | Hs.95162 | KCNQ1 | Potassium voltage-gated channel, KQT-like subfamily, member 1 |
| 137836 | Hs.478150 | PDCD10 | Programmed cell death 10 |
| 359982 | Data not found | | |

| | | | |
|---|---|---|---|
| 767828 | Hs.404568 | HPS1 | Hermansky-Pudlak syndrome 1 |
| 46938 | In multiple ClusterIDs | | |
| 23514 | Hs.731575 | | TAS2R14 |
| 770704 | In multiple ClusterIDs | | |
| 298303 | Hs.310511 | CCL25 | Chemokine (C-C motif) ligand 25 |
| 855487 | Hs.527412 | ASAH1 | N-acylsphingosine amidohydrolase (acid ceramidase) 1 |
| 45645 | Hs.155090 | GNB5 | Guanine nucleotide binding protein (G protein), beta 5 |
| 725188 | Hs.526521 | MDH1 | Malate dehydrogenase 1, NAD (soluble) |
| 869538 | Hs.50098 | NDUFA4 | NADH dehydrogenase (ubiquinone) 1 alpha subcomplex, 4, 9kDa |
| 866702 | Hs.436142 | PTPN13 | Protein tyrosine phosphatase, non-receptor type 13 (APO-1/CD95 (Fas)-associated phosphatase) |
| 796759 | Hs.699301 | VDAC3 | Voltage-dependent anion channel 3 |
| 859422 | Hs.466148 | NR2F6 | Nuclear receptor subfamily 2, group F, member 6 |
| 869187 | Hs.468410 | EPAS1 | Endothelial PAS domain protein 1 |
| 236333 | Hs.699822 | ISLR | Immunoglobulin superfamily containing leucine-rich repeat |
| 823691 | In multiple ClusterIDs | | |
| 813818 | Hs.652255 | RRP8 | Ribosomal RNA processing 8, methyltransferase, homolog (yeast) |
| 897497 | Hs.659350 | HOXA10 | Homeobox A10 |
| 179534 | Hs.161851 | KCNQ2 | Potassium voltage-gated channel, KQT-like subfamily, member 2 |
| 813171 | Hs.372541 | KBTBD2 | Kelch repeat and BTB (POZ) domain containing 2 |
| 241985 | Hs.731866 | | SNRPN |
| 212649 | Hs.1498 | HRG | Histidine-rich glycoprotein |
| 129146 | In multiple ClusterIDs | | |
| 187266 | Hs.409137 | | EIF2B2 |
| 813591 | Hs.444776 | | ACOT8 |
| 363103 | Hs.597910 | | Transcribed locus |
| 768497 | Hs.143961 | CCL18 | Chemokine (C-C motif) ligand 18 (pulmonary and activation-regulated) |
| 246524 | Hs.24529 | CHEK1 | Checkpoint kinase 1 |
| 771084 | In multiple ClusterIDs | | |

| | | | |
|---|---|---|---|
| 211780 | Hs.69293 | HEXB | Hexosaminidase B (beta polypeptide) |
| 363575 | In multiple ClusterIDs | | |
| 770462 | Hs.61995 | CPZ | Carboxypeptidase Z |
| 511850 | In multiple ClusterIDs | | |
| 684655 | Hs.437366 | PSMC2 | Proteasome (prosome, macropain) 26S subunit, ATPase, 2 |
| 1912749 | Hs.505654 | ITGA5 | Integrin, alpha 5 (fibronectin receptor, alpha polypeptide) |
| 669485 | Hs.488293 | EGFR | Epidermal growth factor receptor |
| 435597 | Hs.413899 | HTR3A | 5-hydroxytryptamine (serotonin) receptor 3A, ionotropic |
| 501939 | Hs.461453 | WWOX | WW domain containing oxidoreductase |
| 729316 | In multiple ClusterIDs | | |
| 2114698 | Hs.201805 | LAMC3 | Laminin, gamma 3 |
| 49553 | Hs.183153 | ARL4D | ADP-ribosylation factor-like 4D |
| 25988 | Hs.433750 | EIF4G1 | Eukaryotic translation initiation factor 4 gamma, 1 |
| 154359 | Hs.422585 | TWIST2 | Twist basic helix-loop-helix transcription factor 2 |
| 1185969 | Hs.592082 | AXIN1 | Axin 1 |
| 470122* | Hs.484099 | KCNMB1 | Potassium large conductance calcium-activated channel, subfamily M, beta member 1 |
| 841308* | Hs.477375 | MYLK | Myosin light chain kinase |
| 362059* | Hs.436367 | LAMA3 | Laminin, alpha 3 |
| 813828* | Hs.262857 | PRUNE2 | Prune homolog 2 (Drosophila) |
| 70332 | Hs.532492 | ACP2 | Acid phosphatase 2, lysosomal |
| 85643 | Hs.75599 | SERPINC1 | Serpin peptidase inhibitor, clade C (antithrombin), member 1 |
| 869466 | Hs.529019 | BPI | Bactericidal/permeability-increasing protein |
| 795321 | Hs.116459 | MAN2A2 | Mannosidase, alpha, class 2A, member 2 |
| 823982 | In multiple ClusterIDs | | |
| 769911 | Hs.432760 | CAPZB | Capping protein (actin filament) muscle Z-line, beta |
| 42070 | Hs.153952 | NT5E | 5'-nucleotidase, ecto (CD73) |
| 868838 | Hs.596913 | HPGD | Hydroxyprostaglandin dehydrogenase 15-(NAD) |
| 825604 | Hs.262886 | INPP5D | Inositol polyphosphate-5-phosphatase, 145kDa |
| 460487 | Hs.529517 | LTF | Lactotransferrin |
| 838366 | Hs.533444 | HMGCL | 3-hydroxymethyl-3-methylglutaryl-CoA lyase |
| 506658 | Hs.503692 | YAP1 | Yes-associated protein 1 |
| 25807 | Hs.517044 | PHF20 | PHD finger protein 20 |
| 2223790 | Hs.162807 | TFF1 | Trefoil factor 1 |
| 284799 | Hs.659607 | | Transcribed locus |

| | | | |
|---|---|---|---|
| 430314 | Hs.2899 | HPD | 4-hydroxyphenylpyruvate dioxygenase |
| 1854658 | Hs.744928 | PTPRS | Protein tyrosine phosphatase, receptor type, S |
| 2108253 | Hs.471119 | BMPR2 | Bone morphogenetic protein receptor, type II (serine/threonine kinase) |
| 193736 | Hs.34012 | BRCA2 | Breast cancer 2, early onset |
| 121454 | Hs.654431 | ALOX12 | Arachidonate 12-lipoxygenase |
| 796323 | Hs.501012 | ADD3 | Adducin 3 (gamma) |
| 845519 | Hs.271135 | ATP5C1 | ATP synthase, H+ transporting, mitochondrial F1 complex, gamma polypeptide 1 |
| 740801 | Hs.433307 | BCKDHA | Branched chain keto acid dehydrogenase E1, alpha polypeptide |
| 714213 | Hs.244139 | FAS | Fas (TNF receptor superfamily, member 6) |
| 192569 | Hs.368794 | AP1B1 | Adaptor-related protein complex 1, beta 1 subunit |
| 815303 | Hs.503787 | DARS | Aspartyl-tRNA synthetase |
| 797048 | Hs.68879 | BMP4 | Bone morphogenetic protein 4 |
| 856454 | Hs.502769 | SLC3A2 | Solute carrier family 3 (activators of dibasic and neutral amino acid transport), member 2 |
| 896962 | Hs.507076 | ACADS | Acyl-CoA dehydrogenase, C-2 to C-3 short chain |
| 51950 | Hs.76152 | AQP1 | Aquaporin 1 (Colton blood group) |
| 25517 | Hs.517830 | BTD | Biotinidase |
| 454672 | Hs.499725 | ANK3 | Ankyrin 3, node of Ranvier (ankyrin G) |
| 868304* | Hs.500483 | ACTA2 | Actin, alpha 2, smooth muscle, aorta |
| 588822 | Hs.171458 | ENOX2 | Ecto-NOX disulfide-thiol exchanger 2 |
| 756533 | Hs.501293 | BSG | Basigin (Ok blood group) |
| 74537 | Hs.518808 | AFP | Alpha-fetoprotein |
| 1736335 | Hs.395482 | PTK2 | Protein tyrosine kinase 2 |
| 1337007 | Hs.518451 | PIK3CD | Phosphatidylinositol-4,5-bisphosphate 3-kinase, catalytic subunit delta |
| 2513307 | Hs.530402 | FAF1 | Fas (TNFRSF6) associated factor 1 |
| 2383518 | Hs.744057 | THRAP3 | Thyroid hormone receptor associated protein 3 |
| 2164312 | Hs.119594 | CIT | Citron (rho-interacting, serine/threonine kinase 21) |
| 156 | In multiple ClusterIDs | | |
| 725368 | Hs.521912 | RHPN1 | Rhophilin, Rho GTPase binding protein 1 |
| 686909 | Hs.127897 | RAPGEF1 | Rap guanine nucleotide exchange factor (GEF) 1 |
| 1743966 | Hs.180919 | ID2 | Inhibitor of DNA binding 2, dominant negative helix-loop-helix protein |

| | | | |
|---|---|---|---|
| 1 | In multiple ClusterIDs | | |
| 2329149 | Hs.16184 | RAD17 | RAD17 homolog (S. pombe) |
| 1028344 | Hs.368410 | CBX2 | Chromobox homolog 2 |
| 221197 | Hs.435845 | ESRRB | Estrogen-related receptor beta |
| 648794 | Hs.660607 | ESR2 | Estrogen receptor 2 (ER beta) |
| 84148 | Hs.57732 | MAPK11 | Mitogen-activated protein kinase 11 |
| 2298363 | Data not found | | |
| 1018386 | Hs.681743 | ROCK2 | Rho-associated, coiled-coil containing protein kinase 2 |
| 767236 | In multiple ClusterIDs | | |
| 783998 | Hs.591085 | MLLT3 | Myeloid/lymphoid or mixed-lineage leukemia (trithorax homolog, Drosophila); translocated to, 3 |
| 1475746 | Hs.655205 | ZNF124 | Zinc finger protein 124 |
| 1471829 | Hs.529631 | RPL35A | Ribosomal protein L35a |
| 767475 | In multiple ClusterIDs | | |
| 897768 | Hs.476218 | COL7A1 | Collagen, type VII, alpha 1 |
| 1476065 | Hs.209983 | STMN1 | Stathmin 1 |
| 1472689 | Hs.110675 | APOC1 | Apolipoprotein C-I |
| 786537 | Hs.518155 | PPP2R3A | Protein phosphatase 2, regulatory subunit B", alpha |
| 753157 | In multiple ClusterIDs | | |
| 1323328 | Hs.1019 | PTH1R | Parathyroid hormone 1 receptor |
| 1325816 | Hs.441072 | POLR2L | Polymerase (RNA) II (DNA directed) polypeptide L, 7.6kDa |
| 825333 | Hs.29679 | MED23 | Mediator complex subunit 23 |
| 978929 | Hs.729514 | | Homo sapiens, clone IMAGE:5122250, mRNA |
| 265241 | Hs.42146 | PAX3 | Paired box 3 |
| 2240486 | Hs.21907 | KAT7 | K(lysine) acetyltransferase 7 |
| 2166340 | Hs.106070 | CDKN1C | Cyclin-dependent kinase inhibitor 1C (p57, Kip2) |
| 1144472 | Hs.497353 | MED6 | Mediator complex subunit 6 |
| 146605 | Hs.99855 | FPR2 | Formyl peptide receptor 2 |
| 156473 | Hs.212088 | EPHX2 | Epoxide hydrolase 2, cytoplasmic |
| 812126 | Hs.654368 | GYPB | Glycophorin B (MNS blood group) |
| 197888 | Hs.5662 | GNB2L1 | Guanine nucleotide binding protein (G protein), beta polypeptide 2-like 1 |
| 381931 | Hs.594454 | FLT1 | Fms-related tyrosine kinase 1 |
| 137017 | Hs.488293 | EGFR | Epidermal growth factor receptor |
| 267865 | In multiple ClusterIDs | | |
| 283034 | Hs.372616 | ARL1 | ADP-ribosylation factor-like 1 |

| | | | |
|---|---|---|---|
| 1469292 | Hs.727148 | PIM2 | Pim-2 oncogene |
| 1456900 | Hs.109 | DPEP1 | Dipeptidase 1 (renal) |
| 626822 | Hs.130031 | TRIO | Trio Rho guanine nucleotide exchange factor |
| 897158 | Hs.247077 | RHOA | Ras homolog family member A |
| 1475595 | Hs.75431 | ALPL | Alkaline phosphatase, liver/bone/kidney |
| 1473274* | Hs.504687 | MYL9 | Myosin, light chain 9, regulatory |
| 321389 | Hs.534453 | NDUFA13 | NADH dehydrogenase (ubiquinone) 1 alpha subcomplex, 13 |
| 882522 | Hs.160786 | ASS1 | Argininosuccinate synthase 1 |
| 1470048 | Hs.521903 | LY6E | Lymphocyte antigen 6 complex, locus E |
| 1325751 | Hs.571258 | CYP3A5 | Cytochrome P450, family 3, subfamily A, polypeptide 5 |
| 296998 | Hs.655792 | ART4 | ADP-ribosyltransferase 4 (Dombrock blood group) |
| 768246 | In multiple ClusterIDs | | |
| 221092 | Hs.654350 | GABPB1 | GA binding protein transcription factor, beta subunit 1 |
| 241788 | Hs.300774 | FGB | Fibrinogen beta chain |
| 563444 | Hs.125898 | GNAS | GNAS complex locus |
| 741474 | Hs.466471 | GPI | Glucose-6-phosphate isomerase |
| 183200 | Hs.73875 | FAH | Fumarylacetoacetate hydrolase (fumarylacetoacetase) |
| 135449 | Hs.374477 | EWSR1 | Ewing sarcoma breakpoint region 1 |
| 178779 | Hs.136295 | GNAL | Guanine nucleotide binding protein (G protein), alpha activating activity polypeptide, olfactory type |
| 127509 | Hs.436062 | GBE1 | Glucan (1,4-alpha-), branching enzyme 1 |
| 133213 | Hs.390420 | | FUT4 |
| 209296 | In multiple ClusterIDs | | |
| 195340 | Hs.418497 | GC | Group-specific component (vitamin D binding protein) |
| 681906 | Hs.1422 | FGR | Gardner-Rasheed feline sarcoma viral (v-fgr) oncogene homolog |
| 669419 | Hs.20685 | FXN | Frataxin |
| 49509 | Hs.631624 | EPOR | Erythropoietin receptor |
| 308041 | In multiple ClusterIDs | | |
| 26162 | Hs.116250 | GABRA2 | Gamma-aminobutyric acid (GABA) A receptor, alpha 2 |
| 591683 | Hs.80409 | | GADD45A |
| 839736 | Hs.53454 | CRYAB | Crystallin, alpha B |
| 825335 | Hs.185597 | SPG7 | Spastic paraplegia 7 (pure and complicated autosomal recessive) |

| | | | |
|---|---|---|---|
| 77805 | Hs.681616 | COPB2 | Coatomer protein complex, subunit beta 2 (beta prime) |
| 767817 | Hs.436578 | POLR2F | Polymerase (RNA) II (DNA directed) polypeptide F |
| 701231 | Hs.115617 | CRHBP | Corticotropin releasing hormone binding protein |
| 898286 | Hs.732435 | CDK1 | Cyclin-dependent kinase 1 |
| 310519 | Hs.361463 | F10 | Coagulation factor X |
| 630013 | Hs.597656 | MSH2 | MutS homolog 2, colon cancer, nonpolyposis type 1 (E. coli) |
| 418422 | Hs.2164 | PPBP | Pro-platelet basic protein (chemokine (C-X-C motif) ligand 7) |
| 687875 | Hs.181301 | CTSS | Cathepsin S |
| 897655 | Hs.196983 | SSFA2 | Sperm specific antigen 2 |
| 197657* | Hs.436219 | ALDH1B1 | Aldehyde dehydrogenase 1 family, member B1 |
| 248463 | Hs.585357 | HBZ | Hemoglobin, zeta |
| 295986 | Hs.30619 | EBP | Emopamil binding protein (sterol isomerase) |
| 745339 | Hs.279912 | CCP110 | Centriolar coiled coil protein 110kDa |
| 743701 | Hs.106311 | DGCR9 | DiGeorge syndrome critical region gene 9 |
| 253009 | Hs.642877 | MALAT1 | Metastasis associated lung adenocarcinoma transcript 1 (non-protein coding) |
| 741958 | In multiple ClusterIDs | | |
| 784777 | Hs.183850 | DCTD | DCMP deaminase |
| 782760 | Hs.644076 | | Transcribed locus |
| 796984 | In multiple ClusterIDs | | |
| 838568 | Hs.351875 | COX6C | Cytochrome c oxidase subunit VIc |
| 825060 | Hs.527980 | DUT | Deoxyuridine triphosphatase |
| 795296 | Hs.292524 | CCNH | Cyclin H |
| 756211 | Hs.443417 | MINK1 | Misshapen-like kinase 1 |
| 51814 | Hs.695 | CSTB | Cystatin B (stefin B) |
| 824031 | Hs.445203 | DNAJA1 | DnaJ (Hsp40) homolog, subfamily A, member 1 |
| 950690 | Hs.58974 | CCNA2 | Cyclin A2 |
| 795830 | Hs.479867 | CENPC1 | Centromere protein C 1 |
| 505059 | Hs.195040 | HSD11B1 | Hydroxysteroid (11-beta) dehydrogenase 1 |
| 297895 | Hs.436066 | LST1 | Leukocyte specific transcript 1 |
| 856427 | Hs.436187 | TRIP13 | Thyroid hormone receptor interactor 13 |
| 343700 | In multiple ClusterIDs | | |
| 295857 | Hs.50282 | RRAGB | Ras-related GTP binding B |
| 447786 | Hs.21631 | AUTS2 | Autism susceptibility candidate 2 |

| | | | |
|---|---|---|---|
| 131268 | Hs.411881 | GRB14 | Growth factor receptor-bound protein 14 |
| 745116 | Hs.284279 | HMOX2 | Heme oxygenase (decycling) 2 |
| 293964 | Hs.191510 | BTN3A1 | Butyrophilin, subfamily 3, member A1 |
| 742949 | In multiple ClusterIDs | | |
| 502565 | Hs.501735 | STIM1 | Stromal interaction molecule 1 |
| 361698 | Hs.656980 | LIPE | Lipase, hormone-sensitive |
| 854899 | Hs.298654 | DUSP6 | Dual specificity phosphatase 6 |
| 281881 | Hs.269211 | ZMYM4 | Zinc finger, MYM-type 4 |
| 175536 | Hs.113882 | GABRD | Gamma-aminobutyric acid (GABA) A receptor, delta |
| 1031076 | Hs.98428 | HOXB6 | Homeobox B6 |
| 26249 | Hs.75149 | SH3GL2 | SH3-domain GRB2-like 2 |
| 590591 | Hs.525401 | ADCY6 | Adenylate cyclase 6 |
| 624429 | In multiple ClusterIDs | | |
| 303035 | Hs.497626 | PLXNA2 | Plexin A2 |
| 415769 | Hs.369675 | | ANGPT1 |
| 21908 | In multiple ClusterIDs | | |
| 182177 | Hs.404914 | ADAM17 | ADAM metallopeptidase domain 17 |
| 309864 | Hs.25292 | JUNB | Jun B proto-oncogene |
| 416567 | Hs.741309 | | Transcribed locus, strongly similar to NP_000615.3 SERPINA5 gene product [Homo sapiens] |
| 25621 | Hs.160958 | CDC37 | Cell division cycle 37 |
| 201268 | Hs.288741 | EFNA5 | Ephrin-A5 |
| 322525 | Hs.4817 | OPCML | Opioid binding protein/cell adhesion molecule-like |
| 428223 | Hs.472437 | MAPRE1 | Microtubule-associated protein, RP/EB family, member 1 |
| 38059 | Hs.530871 | PDE1B | Phosphodiesterase 1B, calmodulin-dependent |
| 222107 | Hs.479756 | KDR | Kinase insert domain receptor (a type III receptor tyrosine kinase) |
| 324383 | Hs.284244 | FGF2 | Fibroblast growth factor 2 (basic) |
| 433528 | Hs.631624 | EPOR | Erythropoietin receptor |
| 47021 | Hs.731450 | DVL1 | Dishevelled, dsh homolog 1 (Drosophila) |
| 245761 | Hs.517601 | RAC2 | Ras-related C3 botulinum toxin substrate 2 (rho family, small GTP binding protein Rac2) |
| 325487 | Hs.505654 | ITGA5 | Integrin, alpha 5 (fibronectin receptor, alpha polypeptide) |
| 436121 | Hs.533717 | DLK1 | Delta-like 1 homolog (Drosophila) |
| 884438 | Hs.744006 | NFE2L2 | Nuclear factor (erythroid-derived 2)-like 2 |

| | | | |
|---|---|---|---|
| 770454 | Hs.108969 | WDR83OS | WD repeat domain 83 opposite strand |
| 884644 | Hs.449909 | RPSA | Ribosomal protein SA |
| 491486 | In multiple ClusterIDs | | |
| 447509 | Hs.631991 | HLA-DOA | Major histocompatibility complex, class II, DO alpha |
| 138369 | Hs.654395 | FCGR2B | Fc fragment of IgG, low affinity IIb, receptor (CD32) |
| 346696 | Hs.94865 | TEAD4 | TEA domain family member 4 |
| 83444 | Hs.952 | SLC10A1 | Solute carrier family 10 (sodium/bile acid cotransporter family), member 1 |
| 72395 | Hs.655432 | MST1L | Macrophage stimulating 1-like |
| 586796 | Hs.84905 | KRT20 | Keratin 20 |
| 755299 | Hs.501629 | IER2 | Immediate early response 2 |
| 241847 | Hs.513522 | | Transcribed locus |
| 160664 | Hs.350321 | RET | Ret proto-oncogene |
| 292779 | Hs.388034 | RXRB | Retinoid X receptor, beta |
| 377671 | Hs.524484 | ITGA7 | Integrin, alpha 7 |
| 365348 | Hs.46894 | FUS | Fused in sarcoma |
| 173392 | Hs.647388 | ARHGDIG | Rho GDP dissociation inhibitor (GDI) gamma |
| 810974 | Hs.3068 | HLTF | Helicase-like transcription factor |
| 135527 | Hs.458917 | SCAMP2 | Secretory carrier membrane protein 2 |
| 159455 | Hs.257008 | PLD3 | Phospholipase D family, member 3 |
| 771206 | Hs.306791 | POLD2 | Polymerase (DNA directed), delta 2, accessory subunit |
| 35077 | Hs.21639 | | SPEG |
| 810761 | Hs.534377 | CLDN10 | Claudin 10 |
| 731648 | Hs.10441 | NFYA | Nuclear transcription factor Y, alpha |
| 759163 | Hs.296049 | MFAP4 | Microfibrillar-associated protein 4 |
| 855910 | Hs.531081 | LGALS3 | Lectin, galactoside-binding, soluble, 3 |
| 361899 | Hs.157883 | ZSCAN26 | Zinc finger and SCAN domain containing 26 |
| 878835 | Hs.522615 | NDP | Norrie disease (pseudoglioma) |
| 45600 | Hs.130607 | MVK | Mevalonate kinase |
| 324618 | Hs.632704 | TSFM | Ts translation elongation factor, mitochondrial |
| 590692 | Hs.517033 | TGM2 | Transglutaminase 2 (C polypeptide, protein-glutamine-gamma-glutamyltransferase) |
| 868484 | Hs.715623 | NPC1 | Niemann-Pick disease, type C1 |
| 784589 | Hs.80343 | MMP15 | Matrix metallopeptidase 15 (membrane-inserted) |
| 857264 | Hs.600913 | | Transcribed locus |
| 502669 | Hs.3352 | HDAC2 | Histone deacetylase 2 |

| | | | |
|---|---|---|---|
| 194005 | Hs.658489 | | CLCC1 |
| 247281 | Hs.46468 | CCR6 | Chemokine (C-C motif) receptor 6 |
| 548957 | In multiple ClusterIDs | | |
| 202535 | In multiple ClusterIDs | | |
| 138917 | Hs.437922 | MYCL1 | V-myc myelocytomatosis viral oncogene homolog 1, lung carcinoma derived (avian) |
| 123400 | Hs.727344 | KHSRP | KH-type splicing regulatory protein |
| 213136 | Hs.519162 | BTG2 | BTG family, member 2 |
| 812105 | Hs.75823 | MLLT11 | Myeloid/lymphoid or mixed-lineage leukemia (trithorax homolog, Drosophila); translocated to, 11 |
| 307553 | Hs.505033 | KRAS | V-Ki-ras2 Kirsten rat sarcoma viral oncogene homolog |
| 813827 | In multiple ClusterIDs | | |
| 175123 | Hs.150136 | MAPK7 | Mitogen-activated protein kinase 7 |
| 135773 | Hs.442707 | TERF1 | Telomeric repeat binding factor (NIMA-interacting) 1 |
| 34852 | In multiple ClusterIDs | | |
| 361097 | Hs.518773 | UBE2D3 | Ubiquitin-conjugating enzyme E2D 3 |
| 813184 | Hs.76090 | TNFAIP1 | Tumor necrosis factor, alpha-induced protein 1 (endothelial) |
| 193067 | In multiple ClusterIDs | | |
| 128783 | Hs.161377 | PEX13 | Peroxisomal biogenesis factor 13 |
| 327150 | Hs.159195 | DOCK1 | Dedicator of cytokinesis 1 |
| 38763 | Hs.296169 | | RAB4A |
| 241874 | Hs.441378 | ACAD11 | Acyl-CoA dehydrogenase family, member 11 |
| 293539 | Data not found | | |
| 202213 | Hs.348350 | DHRS1 | Dehydrogenase/reductase (SDR family) member 1 |
| 66534 | Hs.89771 | GCKR | Glucokinase (hexokinase 4) regulator |
| 140354 | Hs.144980 | SCAMP4 | Secretory carrier membrane protein 4 |
| 137638 | Hs.462693 | | ZNF22 |
| 248649 | Hs.75277 | RMND5A | Required for meiotic nuclear division 5 homolog A (S. cerevisiae) |
| 280882 | Hs.504281 | FLI1 | Friend leukemia virus integration 1 |
| 126792 | Hs.731768 | | Transcribed locus |
| 165921 | Hs.443976 | CEP250 | Centrosomal protein 250kDa |
| 292920 | Hs.432706 | ANKRD44 | Ankyrin repeat domain 44 |
| 22770* | Hs.7195 | GABRG2 | Gamma-aminobutyric acid (GABA) A receptor, gamma 2 |

| | | | |
|---|---|---|---|
| 1674185* | Hs.351665 | TRDMT1 | TRNA aspartic acid methyltransferase 1 |
| 5222483* | Hs.463412 | SGCA | Sarcoglycan, alpha (50kDa dystrophin-associated glycoprotein) |
| 432651 | Hs.44402 | CSTF3 | Cleavage stimulation factor, 3' pre-RNA, subunit 3, 77kDa |
| 744417 | Hs.12068 | CRAT | Carnitine O-acetyltransferase |
| 26568 | Hs.534313 | EGR3 | Early growth response 3 |
| 757144 | In multiple ClusterIDs | | |
| 264640 | Hs.484241 | CLTB | Clathrin, light chain B |
| 26295 | Hs.75110 | CNR1 | Cannabinoid receptor 1 (brain) |
| 83083 | Hs.76716 | ITIH3 | Inter-alpha-trypsin inhibitor heavy chain 3 |
| 201168 | Hs.412836 | LRRC8C | Leucine rich repeat containing 8 family, member C |
| 133114 | Hs.25338 | PRSS23 | Protease, serine, 23 |
| 344672 | Hs.15536 | ZCCHC9 | Zinc finger, CCHC domain containing 9 |
| 196387 | Hs.368243 | ABCC2 | ATP-binding cassette, sub-family C (CFTR/MRP), member 2 |
| 140100 | Hs.486410 | ECHDC1 | Enoyl CoA hydratase domain containing 1 |
| 122822 | Hs.462323 | NCOR1 | Nuclear receptor corepressor 1 |
| 230562 | Hs.124490 | ACIN1 | Apoptotic chromatin condensation inducer 1 |
| 127925 | Hs.744922 | DDX18 | DEAD (Asp-Glu-Ala-Asp) box polypeptide 18 |
| 246035 | In multiple ClusterIDs | | |
| 824044 | Hs.159069 | LARP4B | La ribonucleoprotein domain family, member 4B |
| 293403 | Hs.127411 | CDC14A | Cell division cycle 14A |
| 251591 | Hs.283565 | FOSL1 | FOS-like antigen 1 |
| 759200 | Hs.79064 | DHPS | Deoxyhypusine synthase |
| 868652 | Hs.534847 | C4A | Complement component 4A (Rodgers blood group) |
| 858292 | Hs.191346 | Sep-07 | Septin 7 |
| 39722 | Hs.487294 | ERCC2 | Excision repair cross-complementing rodent repair deficiency, complementation group 2 |
| 769579 | Hs.465627 | MAP2K2 | Mitogen-activated protein kinase kinase 2 |
| 756556 | Hs.384598 | SERPING1 | Serpin peptidase inhibitor, clade G (C1 inhibitor), member 1 |
| 345538 | Hs.731507 | CTSL1 | Cathepsin L1 |
| 884894 | Hs.151777 | EIF2S1 | Eukaryotic translation initiation factor 2, subunit 1 alpha, 35kDa |
| 469369 | Hs.24301 | POLR2E | Polymerase (RNA) II (DNA directed) polypeptide E, 25kDa |
| 878280 | Hs.135270 | CRMP1 | Collapsin response mediator protein 1 |
| 897164 | Hs.445981 | CTNNA1 | Catenin (cadherin-associated protein), alpha 1, 102kDa |
| 47359 | Hs.511899 | EDN1 | Endothelin 1 |

| | | | |
|---|---|---|---|
| 128493 | Hs.195364 | MLH1 | MutL homolog 1, colon cancer, nonpolyposis type 2 (E. coli) |
| 491692 | Hs.17441 | COL4A1 | Collagen, type IV, alpha 1 |
| 854138 | Hs.474833 | CSNK1E | Casein kinase 1, epsilon |
| 781017 | Hs.1395 | EGR2 | Early growth response 2 |
| 345063 | Hs.635 | CACNB1 | Calcium channel, voltage-dependent, beta 1 subunit |
| 1926453 | Hs.270833 | AREG | Amphiregulin |
| 2119600 | Hs.460996 | TRADD | TNFRSF1A-associated via death domain |
| 1650500 | Hs.603111 | RND2 | Rho family GTPase 2 |
| 1741102 | Hs.279594 | TNFRSF1A | Tumor necrosis factor receptor superfamily, member 1A |
| 1927412 | Hs.631709 | RAD51 | RAD51 homolog (S. cerevisiae) |
| 2139081 | Hs.478150 | PDCD10 | Programmed cell death 10 |
| 1656179 | Hs.113157 | ITGA9 | Integrin, alpha 9 |
| 1743833 | Hs.516664 | EFNA1 | Ephrin-A1 |
| 1932948 | Hs.673 | IL12A | Interleukin 12A (natural killer cell stimulatory factor 1, cytotoxic lymphocyte maturation factor 1, p35) |
| 2146122 | Hs.133397 | ITGA6 | Integrin, alpha 6 |
| 1667384 | Hs.190028 | GSTO1 | Glutathione S-transferase omega 1 |
| 1752018 | Hs.507590 | FLT3 | Fms-related tyrosine kinase 3 |
| 1935352 | Hs.2159 | ACAN | Aggrecan |
| 2149968 | Hs.647029 | FZD9 | Frizzled family receptor 9 |
| 1677204 | Hs.356624 | NID1 | Nidogen 1 |
| 1837155 | Hs.2633 | DSG1 | Desmoglein 1 |
| 2009666 | Hs.116092 | EPHB1 | EPH receptor B1 |
| 2159880 | Hs.419815 | EGF | Epidermal growth factor |
| 1032004 | Hs.415299 | ITPA | Inosine triphosphatase (nucleoside triphosphate pyrophosphatase) |
| 855620 | Hs.505735 | NACA | Nascent polypeptide-associated complex alpha subunit |
| 842973 | Hs.524498 | PA2G4 | Proliferation-associated 2G4, 38kDa |
| 511459 | Hs.658566 | PDIA3P | Protein disulfide isomerase family A, member 3 pseudogene |
| 769942 | Hs.612151 | KIF22 | Kinesin family member 22 |
| 951142 | Hs.409065 | FEN1 | Flap structure-specific endonuclease 1 |
| 594322 | Hs.368610 | PAPSS1 | 3'-phosphoadenosine 5'-phosphosulfate synthase 1 |
| 416643 | Hs.151624 | HCRTR2 | Hypocretin (orexin) receptor 2 |
| 366132 | Hs.444472 | SDHC | Succinate dehydrogenase complex, subunit C, integral membrane protein, 15kDa |
| 626531 | In multiple | | |

| | | | |
|---|---|---|---|
| | ClusterIDs | | |
| 509943 | Hs.4747 | DKC1 | Dyskeratosis congenita 1, dyskerin |
| 784253 | Hs.474938 | SLC25A17 | Solute carrier family 25 (mitochondrial carrier; peroxisomal membrane protein, 34kDa), member 17 |
| 1637226 | Hs.428 | FLT3LG | Fms-related tyrosine kinase 3 ligand |
| 1734372 | Hs.562227 | HSPG2 | Heparan sulfate proteoglycan 2 |
| 1902522 | Hs.632586 | CXCL10 | Chemokine (C-X-C motif) ligand 10 |
| 2108696 | Hs.512599 | CDKN2A | Cyclin-dependent kinase inhibitor 2A |
| 1646946 | Hs.119882 | CDK6 | Cyclin-dependent kinase 6 |
| 1735565 | Hs.187898 | NF2 | Neurofibromin 2 (merlin) |
| 469686 | Hs.491234 | RIT1 | Ras-like without CAAX 1 |
| 166236 | Hs.289108 | FAM3A | Family with sequence similarity 3, member A |
| 66317 | Hs.7644 | HIST1H1C | Histone cluster 1, H1c |
| 27548 | Hs.601591 | NUP153 | Nucleoporin 153kDa |
| 177621 | In multiple ClusterIDs | | |
| 210317 | Hs.626404 | RAB11B | RAB11B, member RAS oncogene family |
| 838149 | Hs.435771 | API5 | Apoptosis inhibitor 5 |
| 530035 | Hs.516505 | S100A13 | S100 calcium binding protein A13 |
| 950568 | Hs.533855 | UPF3A | UPF3 regulator of nonsense transcripts homolog A (yeast) |
| 897865 | Hs.27695 | MID1 | Midline 1 (Opitz/BBB syndrome) |
| 884822 | Hs.50425 | PTGES3 | Prostaglandin E synthase 3 (cytosolic) |
| 530282 | Hs.534168 | NDUFA1 | NADH dehydrogenase (ubiquinone) 1 alpha subcomplex, 1, 7.5kDa |
| 780977 | Hs.523131 | TRAPPC3 | Trafficking protein particle complex 3 |
| 773511 | Hs.30792 | HOOK2 | Hook homolog 2 (Drosophila) |
| 82903 | Hs.370937 | TAPBP | TAP binding protein (tapasin) |
| 324122 | Hs.129944 | ESM1 | Endothelial cell-specific molecule 1 |
| 796946 | In multiple ClusterIDs | | |
| 270136 | In multiple ClusterIDs | | |
| 811827 | Hs.222055 | NDST1 | N-deacetylase/N-sulfotransferase (heparan glucosaminyl) 1 |
| 668442 | Hs.593833 | DDR2 | Discoidin domain receptor tyrosine kinase 2 |
| 154465 | Hs.10136 | BPHL | Biphenyl hydrolase-like (serine hydrolase) |
| 471266 | Hs.410965 | DGCR6L | DiGeorge syndrome critical region gene 6-like |
| 233457 | Hs.302145 | | HBG2 |
| 45272 | Hs.193043 | CLCN6 | Chloride channel, voltage-sensitive 6 |

| | | | |
|---|---|---|---|
| 137794 | Hs.174273 | ACVR2B | Activin A receptor, type IIB |
| 341246 | Hs.515092 | CLPP | ClpP caseinolytic peptidase, ATP-dependent, proteolytic subunit homolog (E. coli) |
| 770066 | Hs.438550 | NCAPD3 | Non-SMC condensin II complex, subunit D3 |
| 179283 | Hs.270055 | SH3GL3 | SH3-domain GRB2-like 3 |
| 194214 | Hs.373550 | | TGIF1 |
| 254321 | In multiple ClusterIDs | | |
| 309316 | Hs.75636 | MYL7 | Myosin, light chain 7, regulatory |
| 234011 | Hs.164384 | PKP2 | Plakophilin 2 |
| 366558 | In multiple ClusterIDs | | |
| 70349 | Hs.584654 | FOXO4 | Forkhead box O4 |
| 153411 | Hs.520048 | HLA-DRA | Major histocompatibility complex, class II, DR alpha |
| 589433 | Hs.484222 | RTCA | RNA 3'-terminal phosphate cyclase |
| 753862 | In multiple ClusterIDs | | |
| 142788 | Hs.596449 | SERPINH1 | Serpin peptidase inhibitor, clade H (heat shock protein 47), member 1, (collagen binding protein 1) |
| 841059 | Hs.516155 | CAPG | Capping protein (actin filament), gelsolin-like |
| 739183 | Hs.647419 | CD68 | CD68 molecule |
| 813830 | Hs.289271 | CYC1 | Cytochrome c-1 |
| 284882 | Hs.408182 | COL2A1 | Collagen, type II, alpha 1 |
| 277015 | Data not found | | |
| 504226 | Hs.443057 | CD53 | CD53 molecule |
| 83231 | Hs.1360 | CYP2B6 | Cytochrome P450, family 2, subfamily B, polypeptide 6 |
| 758148 | Hs.654450 | F8 | Coagulation factor VIII, procoagulant component |
| 69672 | Hs.76206 | CDH5 | Cadherin 5, type 2 (vascular endothelium) |
| 306013 | Hs.438040 | | Transcribed locus |
| 757248 | Hs.733336 | | Transcribed locus |
| 435855 | Hs.729074 | HCG8 | HLA complex group 8 |
| 223128 | Hs.531754 | MAP2K7 | Mitogen-activated protein kinase kinase 7 |
| 757489 | In multiple ClusterIDs | | |
| 869450 | Hs.719951 | RPL11 | Ribosomal protein L11 |
| 487425 | Hs.591767 | CETN3 | Centrin, EF-hand protein, 3 |
| 365641 | Hs.534339 | PRIM1 | Primase, DNA, polypeptide 1 (49kDa) |
| 83210 | In multiple ClusterIDs | | |

| | | | |
|---|---|---|---|
| 898035 | Hs.520898 | CTSB | Cathepsin B |
| 42880 | Hs.382306 | | CDK8 |
| 841478 | Hs.352677 | FBXO11 | F-box protein 11 |
| 85624 | Hs.1012 | C4BPA | Complement component 4 binding protein, alpha |
| 839101 | Hs.74471 | GJA1 | Gap junction protein, alpha 1, 43kDa |
| 843049 | Hs.460184 | MCM4 | Minichromosome maintenance complex component 4 |
| 66564 | Hs.274539 | BDH1 | 3-hydroxybutyrate dehydrogenase, type 1 |
| 85128 | Hs.8986 | C1QB | Complement component 1, q subcomponent, B chain |
| 711552 | Hs.88778 | CBR1 | Carbonyl reductase 1 |
| 795827 | Hs.708096 | TESK1 | Testis-specific kinase 1 |
| 434826 | Hs.558440 | AP5Z1 | Adaptor-related protein complex 5, zeta 1 subunit |
| 433111 | Hs.3459 | UBFD1 | Ubiquitin family domain containing 1 |
| 739109 | Hs.119591 | AP2S1 | Adaptor-related protein complex 2, sigma 1 subunit |
| 611586 | Hs.588655 | NEB | Nebulin |
| 51743 | In multiple ClusterIDs | | |
| 293792 | Hs.408241 | NUPL2 | Nucleoporin like 2 |
| 586854 | Hs.193557 | PTPN14 | Protein tyrosine phosphatase, non-receptor type 14 |
| 1031185 | Hs.111850 | SMCP | Sperm mitochondria-associated cysteine-rich protein |
| 32241 | Hs.594723 | | Transcribed locus |
| 770000 | Hs.723478 | EP400 | E1A binding protein p400 |
| 770337 | Hs.132868 | USP32 | Ubiquitin specific peptidase 32 |
| 460403 | Hs.702528 | | Transcribed locus |
| 298128 | Hs.555902 | ASAP2 | ArfGAP with SH3 domain, ankyrin repeat and PH domain 2 |
| 203130 | Hs.323342 | | ARPC4 |
| 433666 | Hs.474949 | RBX1 | Ring-box 1, E3 ubiquitin protein ligase |
| 60565 | In multiple ClusterIDs | | |
| 248957 | Hs.592737 | | Transcribed locus |
| 49311 | Hs.731384 | MLL2 | Myeloid/lymphoid or mixed-lineage leukemia 2 |
| 345158 | Hs.517352 | | PRODH |
| 484874 | Hs.218040 | ITGB3 | Integrin, beta 3 (platelet glycoprotein IIIa, antigen CD61) |
| 139167 | Hs.510324 | QKI | QKI, KH domain containing, RNA binding |
| 273938 | In multiple ClusterIDs | | |
| 358433 | Hs.26550 | RXRG | Retinoid X receptor, gamma |
| 487777 | Hs.408528 | RB1 | Retinoblastoma 1 |
| 145383 | Hs.401929 | DNASE1L1 | Deoxyribonuclease I-like 1 |

| | | | |
|---|---|---|---|
| 284701 | Hs.654487 | FOLH1 | Folate hydrolase (prostate-specific membrane antigen) 1 |
| 361456 | Hs.505777 | | DDIT3 |
| 491113 | Hs.116471 | CDH11 | Cadherin 11, type 2, OB-cadherin (osteoblast) |
| 146858 | Hs.249441 | WEE1 | WEE1 homolog (S. pombe) |
| 285780 | Hs.2936 | MMP13 | Matrix metallopeptidase 13 (collagenase 3) |
| 364843 | Hs.80828 | KRT1 | Keratin 1 |
| 510856 | In multiple ClusterIDs | | |
| 154790 | Hs.415768 | NGFR | Nerve growth factor receptor |
| 289606 | Hs.168132 | IL15 | Interleukin 15 |
| 366481 | Hs.708950 | KRT6B | Keratin 6B |
| 586803 | Hs.252820 | PGF | Placental growth factor |
| 462953 | Hs.654473 | MAOB | Monoamine oxidase B |
| 853906 | Hs.712829 | | M4-50 mRNA for HLA class I antigen |
| 588915 | Hs.532634 | IFI27 | Interferon, alpha-inducible protein 27 |
| 51022 | Hs.302341 | ST8SIA2 | ST8 alpha-N-acetyl-neuraminide alpha-2,8-sialyltransferase 2 |
| 433553 | Hs.592035 | | Transcribed locus, strongly similar to NP_001730.1 CA5A gene product [Homo sapiens] |
| 345232 | Hs.36 | | LTA |
| 415899 | Hs.500546 | IDE | Insulin-degrading enzyme |
| 770837 | Hs.397891 | PASK | PAS domain containing serine/threonine kinase |
| 586839 | Hs.517949 | MAP4 | Microtubule-associated protein 4 |
| 868169 | Hs.180878 | LPL | Lipoprotein lipase |
| 51582 | Hs.207631 | LMO7 | LIM domain 7 |
| 769948 | Hs.104879 | SERPINB9 | Serpin peptidase inhibitor, clade B (ovalbumin), member 9 |
| 51817 | Hs.517603 | MFNG | MFNG O-fucosylpeptide 3-beta-N-acetylglucosaminyltransferase |
| 257369 | Hs.151413 | GMFB | Glia maturation factor, beta |
| 341763 | Hs.213327 | CASP5 | Caspase 5, apoptosis-related cysteine peptidase |
| 446814 | Hs.522891 | CXCL12 | Chemokine (C-X-C motif) ligand 12 |
| 68049 | Hs.607212 | IGFBP5 | Insulin-like growth factor binding protein 5 |
| 270710 | Hs.370771 | CDKN1A | Cyclin-dependent kinase inhibitor 1A (p21, Cip1) |
| 248454 | Hs.93659 | PDIA4 | Protein disulfide isomerase family A, member 4 |
| 137254 | Hs.732003 | | Transcribed locus |
| 196115 | Hs.34526 | CXCR6 | Chemokine (C-X-C motif) receptor 6 |
| 145503 | Hs.435051 | CDKN2D | Cyclin-dependent kinase inhibitor 2D (p19, inhibits CDK4) |

| | | | |
|---|---|---|---|
| 564846 | Hs.712543 | THOC1 | THO complex 1 |
| 123474 | Hs.558396 | SCD | Stearoyl-CoA desaturase (delta-9-desaturase) |
| 756502 | Hs.534331 | NUDT1 | Nudix (nucleoside diphosphate linked moiety X)-type motif 1 |
| 145383 | Hs.401929 | DNASE1L1 | Deoxyribonuclease I-like 1 |
| 491763 | Hs.126256 | IL1B | Interleukin 1, beta |
| 868368 | Hs.437277 | TMSB4X | Thymosin beta 4, X-linked |
| 743188 | Hs.654530 | NUP214 | Nucleoporin 214kDa |
| 435434 | Hs.163867 | CD14 | CD14 molecule |
| 755599 | Hs.458414 | IFITM1 | Interferon induced transmembrane protein 1 |
| 236059 | Hs.86859 | GRB7 | Growth factor receptor-bound protein 7 |
| 1031203 | Hs.651453 | MYCNOS | MYCN opposite strand/antisense RNA |
| 788256 | Hs.270845 | KIF23 | Kinesin family member 23 |
| 742132 | Hs.458485 | ISG15 | ISG15 ubiquitin-like modifier |
| 432564 | Hs.516160 | SF3B4 | Splicing factor 3b, subunit 4, 49kDa |
| 752557 | Hs.520046 | GPSM3 | G-protein signaling modulator 3 |
| 841357 | Hs.484782 | DFFA | DNA fragmentation factor, 45kDa, alpha polypeptide |
| 813707 | Hs.413297 | RGS16 | Regulator of G-protein signaling 16 |
| 243321 | In multiple ClusterIDs | | |
| 142395 | Hs.83634 | HCFC1 | Host cell factor C1 (VP16-accessory protein) |
| 813675 | Hs.37616 | STRA13 | Stimulated by retinoic acid 13 |
| 531028 | Hs.530862 | PRKAG1 | Protein kinase, AMP-activated, gamma 1 non-catalytic subunit |
| 48182 | Hs.696424 | | Transcribed locus |
| 292272 | Hs.80720 | GAB1 | GRB2-associated binding protein 1 |
| 51737 | Hs.546282 | RBBP8 | Retinoblastoma binding protein 8 |
| 127519 | In multiple ClusterIDs | | |
| 26578 | Hs.517543 | PES1 | Pescadillo ribosomal biogenesis factor 1 |
| 293820 | In multiple ClusterIDs | | |
| 48530 | Hs.169182 | KIF21B | Kinesin family member 21B |
| 810734 | Hs.523829 | POLD4 | Polymerase (DNA-directed), delta 4, accessory subunit |
| 124753 | Hs.94395 | ABCD4 | ATP-binding cassette, sub-family D (ALD), member 4 |
| 209137 | Hs.22785 | GABRE | Gamma-aminobutyric acid (GABA) A receptor, epsilon |
| 135692 | Hs.319438 | PLA2G5 | Phospholipase A2, group V |
| 123614 | Hs.574822 | FAM110A | Family with sequence similarity 110, member A |

| | | | |
|---|---|---|---|
| 292362 | Hs.659592 | | Transcribed locus |
| 251875 | Hs.659011 | | Transcribed locus |
| 136449 | Hs.35125 | ARHGEF40 | Rho guanine nucleotide exchange factor (GEF) 40 |
| 137236 | Hs.508010 | FNDC3A | Fibronectin type III domain containing 3A |
| 471196 | Hs.111577 | ITM2C | Integral membrane protein 2C |
| 110503 | In multiple ClusterIDs | | |
| 843426 | Hs.642842 | SAV1 | Salvador homolog 1 (Drosophila) |
| 214916 | Hs.591863 | FZD6 | Frizzled family receptor 6 |
| 212325 | Hs.443169 | HEPACAM2 | HEPACAM family member 2 |
| 140515 | Data not found | | |
| 123627 | Hs.580464 | ATP6V1C2 | ATPase, H+ transporting, lysosomal 42kDa, V1 subunit C2 |
| 110811* | Hs.484099 | KCNMB1 | Potassium large conductance calcium-activated channel, subfamily M, beta member 1 |
| 729912* | Hs.351665 | TRDMT1 | TRNA aspartic acid methyltransferase 1 |
| 726779* | Hs.465929 | CNN1 | Calponin 1, basic, smooth muscle |
| 81289* | Hs.516105 | ACTG2 | Actin, gamma 2, smooth muscle, enteric |
| 809720 | Hs.516855 | CENPB | Centromere protein B, 80kDa |
| 530185 | Hs.595133 | CD83 | CD83 molecule |
| 291985 | Hs.302738 | SLC26A2 | Solute carrier family 26 (sulfate transporter), member 2 |
| 436062 | Hs.476982 | CPOX | Coproporphyrinogen oxidase |
| 859586 | Hs.467637 | CDC42 | Cell division cycle 42 |
| 431655 | Hs.166556 | CD37 | CD37 molecule |
| 77577 | Hs.220971 | FOSL2 | FOS-like antigen 2 |
| 194006 | Hs.586984 | | Transcribed locus |
| 139641 | Hs.539253 | | Transcribed locus |
| 127843 | Data not found | | |
| 788285 | Hs.183713 | EDNRA | Endothelin receptor type A |
| 136802 | Data not found | | |
| 126338 | Hs.374067 | UBE3B | Ubiquitin protein ligase E3B |
| 209518 | Hs.436787 | | CDNA FLJ25860 fis, clone CBR01771 |
| 199251 | Data not found | | |
| 199628 | Hs.572686 | | |
| 208741 | Hs.658618 | | Transcribed locus |
| 124922 | Hs.172602 | | ZNF589 |
| 796197 | Hs.495912 | DMD | Dystrophin |

| | | | |
|---|---|---|---|
| 128753 | Hs.728936 | CYP17A1-AS1 | CYP17A1 antisense RNA 1 |
| 756629 | Hs.150540 | LMF2 | Lipase maturation factor 2 |
| 730002 | Hs.436186 | CAST | Calpastatin |
| 487797 | Hs.348418 | DR1 | Down-regulator of transcription 1, TBP-binding (negative cofactor 2) |
| 46166 | Hs.152096 | CYP2J2 | Cytochrome P450, family 2, subfamily J, polypeptide 2 |
| 322148 | Hs.516874 | CHGB | Chromogranin B (secretogranin 1) |
| 325182 | Hs.464829 | CDH2 | Cadherin 2, type 1, N-cadherin (neuronal) |
| 742115 | Hs.403436 | ECI1 | Enoyl-CoA delta isomerase 1 |
| 769857 | Hs.533013 | CBS | Cystathionine-beta-synthase |
| 854338 | Hs.514840 | CHI3L2 | Chitinase 3-like 2 |
| 345957 | Hs.518198 | CSTA | Cystatin A (stefin A) |
| 343987 | Hs.368912 | DPP4 | Dipeptidyl-peptidase 4 |
| 757873 | Hs.662611 | | Transcribed locus, strongly similar to NP_034001.1 Cdk5r1 gene product [Mus musculus] |
| 223350 | Hs.558314 | CP | Ceruloplasmin (ferroxidase) |
| 745503 | Hs.518249 | CNBP | CCHC-type zinc finger, nucleic acid binding protein |
| 271006 | In multiple ClusterIDs | | |
| 68950 | Hs.244723 | | CCNE1 |
| 2027965 | Hs.534307 | CCND3 | Cyclin D3 |
| 2166340 | Hs.106070 | CDKN1C | Cyclin-dependent kinase inhibitor 1C (p57, Kip2) |
| 1709032 | Hs.643120 | IGF1R | Insulin-like growth factor 1 receptor |
| 1850392 | Hs.523930 | TRAF5 | TNF receptor-associated factor 5 |
| 2062620 | Hs.156346 | TOP2A | Topoisomerase (DNA) II alpha 170kDa |
| 1881408 | Hs.656646 | GTF3C4 | General transcription factor IIIC, polypeptide 4, 90kDa |
| 1722714 | Hs.76272 | KDM5A | Lysine (K)-specific demethylase 5A |
| 1855159 | Hs.76206 | CDH5 | Cadherin 5, type 2 (vascular endothelium) |
| 2067403 | Hs.388116 | DVL3 | Dishevelled, dsh homolog 3 (Drosophila) |
| 738506 | Hs.196054 | HDAC9 | Histone deacetylase 9 |
| 1722884 | Hs.143751 | MMP11 | Matrix metallopeptidase 11 (stromelysin 3) |
| 1859519 | Hs.371249 | PTN | Pleiotrophin |
| 2095412 | Hs.645344 | RPL13AP5 | Ribosomal protein L13a pseudogene 5 |
| 2485436 | Hs.3352 | HDAC2 | Histone deacetylase 2 |
| 1731368 | Hs.439726 | LAMB2 | Laminin, beta 2 (laminin S) |

| | | | |
|---|---|---|---|
| 1891918 | Hs.90073 | CSE1L | CSE1 chromosome segregation 1-like (yeast) |
| 2104626 | Hs.73133 | MT3 | Metallothionein 3 |
| 843158 | Hs.592142 | NCOA3 | Nuclear receptor coactivator 3 |
| 509800 | In multiple ClusterIDs | | |
| 625683 | Hs.173162 | EMC8 | ER membrane protein complex subunit 8 |
| 951022 | Hs.34780 | DCX | Doublecortin |
| 773579 | Hs.303808 | GCFC2 | GC-rich sequence DNA-binding factor 2 |
| 328207 | Hs.55481 | | ZNF165 |
| 839888 | Hs.7768 | FIBP | Fibroblast growth factor (acidic) intracellular binding protein |
| 593251 | In multiple ClusterIDs | | |
| 772938 | Hs.55967 | SHOX2 | Short stature homeobox 2 |
| 342349 | Hs.404183 | MAP3K14 | Mitogen-activated protein kinase kinase kinase 14 |
| 505225 | Hs.655143 | SORBS2 | Sorbin and SH3 domain containing 2 |
| 271670 | Hs.54673 | TNFSF13 | Tumor necrosis factor (ligand) superfamily, member 13 |
| 771295 | Hs.529420 | UBE2G2 | Ubiquitin-conjugating enzyme E2G 2 |
| 1696513 | Hs.77274 | PLAU | Plasminogen activator, urokinase |
| 1839392 | Hs.170009 | TGFA | Transforming growth factor, alpha |
| 2018080 | Hs.159161 | ARHGDIA | Rho GDP dissociation inhibitor (GDI) alpha |
| 2161635 | Hs.118681 | ERBB3 | V-erb-b2 erythroblastic leukemia viral oncogene homolog 3 (avian) |
| 1705470 | Hs.149239 | EFNB2 | Ephrin-B2 |
| 1844780 | Hs.166015 | FGF6 | Fibroblast growth factor 6 |
| 813158 | Hs.78582 | DRG2 | Developmentally regulated GTP binding protein 2 |
| 275871 | Hs.524599 | NAP1L1 | Nucleosome assembly protein 1-like 1 |
| 301849 | Hs.467084 | EIF4G3 | Eukaryotic translation initiation factor 4 gamma, 3 |
| 199663 | In multiple ClusterIDs | | |
| 770216 | Hs.709218 | EHMT2 | Euchromatic histone-lysine N-methyltransferase 2 |
| 41591 | Hs.268515 | MN1 | Meningioma (disrupted in balanced translocation) 1 |
| 950709 | Hs.182255 | NHP2L1 | NHP2 non-histone chromosome protein 2-like 1 (S. cerevisiae) |
| 593023 | Hs.307720 | DTNB | Dystrobrevin, beta |
| 897575 | Hs.517421 | MED15 | Mediator complex subunit 15 |
| 742977 | In multiple ClusterIDs | | |
| 897823 | Hs.707189 | | Transcribed locus |

| | | | |
|---|---|---|---|
| 731051 | Hs.706903 | | Transcribed locus |
| 610113 | Hs.713554 | SNX2 | Sorting nexin 2 |
| 266085 | Hs.727344 | KHSRP | KH-type splicing regulatory protein |
| 52076 | Hs.522484 | OLFM1 | Olfactomedin 1 |
| 810801 | Hs.273330 | AGRN | Agrin |
| 509570 | Hs.523045 | CAMK2G | Calcium/calmodulin-dependent protein kinase II gamma |
| 784065 | In multiple ClusterIDs | | |
| 767183 | Hs.14601 | HCLS1 | Hematopoietic cell-specific Lyn substrate 1 |
| 134476 | Hs.24167 | VAMP7 | Vesicle-associated membrane protein 7 |
| 812251 | Hs.643566 | MAPKAPK2 | Mitogen-activated protein kinase-activated protein kinase 2 |
| 549146 | Hs.501778 | TRIM22 | Tripartite motif containing 22 |
| 155287 | Hs.702139 | HSPA1A | Heat shock 70kDa protein 1A |
| 810891 | Hs.473256 | LAMA5 | Laminin, alpha 5 |
| 21738 | Hs.438289 | HADH | Hydroxyacyl-CoA dehydrogenase |
| 810813 | Hs.516484 | S100A2 | S100 calcium binding protein A2 |
| 212640 | Hs.701324 | ARHGAP4 | Rho GTPase activating protein 4 |
| 212078 | Hs.644352 | *ITGA1* | integrin, alpha 1 |
| 754046 | Hs.444619 | LAGE3 | L antigen family, member 3 |
| 742101 | Hs.469728 | PAX8 | Paired box 8 |
| 823901 | Hs.202470 | UVRAG | UV radiation resistance associated |
| 138116 | Hs.334868 | PPP2R5E | Protein phosphatase 2, regulatory subunit B', epsilon isoform |
| 154749 | Hs.524081 | DPAGT1 | Dolichyl-phosphate (UDP-N-acetylglucosamine) N-acetylglucosaminephosphotransferase 1 (GlcNAc-1-P transferase) |
| 511066 | Hs.253726 | PAPOLA | Poly(A) polymerase alpha |
| 295729 | Hs.18426 | HRSP12 | Heat-responsive protein 12 |
| 810791 | Hs.509523 | MNAT1 | Menage a trois homolog 1, cyclin H assembly factor (Xenopus laevis) |
| 267634 | Hs.159130 | RAF1 | V-raf-1 murine leukemia viral oncogene homolog 1 |
| 110788 | Hs.56729 | LSP1 | Lymphocyte-specific protein 1 |
| 758365 | Hs.524530 | CTDSP2 | CTD (carboxy-terminal domain, RNA polymerase II, polypeptide A) small phosphatase 2 |
| 787938 | Hs.5462 | SLC4A4 | Solute carrier family 4, sodium bicarbonate cotransporter, member 4 |
| 760148 | Hs.78601 | UROD | Uroporphyrinogen decarboxylase |
| 115281 | Hs.631567 | CD79A | CD79a molecule, immunoglobulin-associated alpha |

| | | | |
|---|---|---|---|
| 788421 | Hs.75862 | SMAD4 | SMAD family member 4 |
| 199180 | Hs.440967 | PON3 | Paraoxonase 3 |
| 191664 | Hs.371147 | THBS2 | Thrombospondin 2 |
| 242578 | Hs.524399 | TROAP | Trophinin associated protein |
| 842939 | Hs.474018 | ADARB1 | Adenosine deaminase, RNA-specific, B1 |
| 841282 | Hs.658169 | SFRP4 | Secreted frizzled-related protein 4 |
| 530139 | Hs.710481 | | Transcribed locus |
| 759173 | Hs.296049 | *MFAP4* | Microfibrillar-associated protein 4 |
| 743804 | Hs.369373 | SEC23B | Sec23 homolog B (S. cerevisiae) |
| 283436 | Hs.132858 | RAP1GDS1 | RAP1, GTP-GDP dissociation stimulator 1 |
| 428412 | Hs.277937 | GZMK | Granzyme K (granzyme 3; tryptase II) |
| 878406 | Hs.490874 | MTX1 | Metaxin 1 |
| 783836 | Hs.196482 | JDP2 | Jun dimerization protein 2 |
| 85805 | Hs.50223 | RBP4 | Retinol binding protein 4, plasma |
| 788486 | Hs.444389 | ENTPD4 | Ectonucleoside triphosphate diphosphohydrolase 4 |
| 83120 | Hs.401509 | RBM10 | RNA binding motif protein 10 |
| 193913 | In multiple ClusterIDs | | |
| 824340 | Hs.648940 | NCF1C | Neutrophil cytosolic factor 1C pseudogene |
| 240505 | Hs.643552 | | SDR39U1 |
| 632137 | Hs.112058 | SIVA1 | SIVA1, apoptosis-inducing factor |
| 258589 | Hs.631886 | REL | V-rel reticuloendotheliosis viral oncogene homolog (avian) |
| 80109 | In multiple ClusterIDs | | |
| 510760 | Hs.478868 | KIAA0226 | KIAA0226 |
| 841695 | Hs.732118 | N4BP2L1 | NEDD4 binding protein 2-like 1 |
| 731273 | Hs.115460 | CCIN | Calicin |
| 277186 | Hs.372295 | PITPNM1 | Phosphatidylinositol transfer protein, membrane-associated 1 |
| 882511 | Hs.277721 | NBR1 | Neighbor of BRCA1 gene 1 |
| 362409 | Hs.420036 | GAD1 | Glutamate decarboxylase 1 (brain, 67kDa) |
| 1031747 | Hs.94542 | ALKBH1 | AlkB, alkylation repair homolog 1 (E. coli) |
| 264556 | Hs.471119 | BMPR2 | Bone morphogenetic protein receptor, type II (serine/threonine kinase) |
| 858153 | Hs.79334 | NFIL3 | Nuclear factor, interleukin 3 regulated |
| 586888 | Hs.77100 | GTF2E2 | General transcription factor IIE, polypeptide 2, beta 34kDa |
| 856174 | Hs.149032 | PIK3R4 | Phosphoinositide-3-kinase, regulatory subunit 4 |

| | | | |
|---|---|---|---|
| 460106 | Hs.486357 | SMPDL3A | Sphingomyelin phosphodiesterase, acid-like 3A |
| 877832 | Hs.522817 | BCAP31 | B-cell receptor-associated protein 31 |
| 288663 | Hs.333303 | GJB1 | Gap junction protein, beta 1, 32kDa |
| 148225 | Hs.170986 | GALNT3 | UDP-N-acetyl-alpha-D-galactosamine:polypeptide N-acetylgalactosaminyltransferase 3 (GalNAc-T3) |
| 271045 | Hs.162233 | CHD4 | Chromodomain helicase DNA binding protein 4 |
| 40773 | Hs.584760 | GNAZ | Guanine nucleotide binding protein (G protein), alpha z polypeptide |
| 34140 | Hs.377894 | GCA | Grancalcin, EF-hand calcium binding protein |
| 586895 | In multiple ClusterIDs | | |
| 1049330 | Hs.183428 | SSPN | Sarcospan |
| 811678 | In multiple ClusterIDs | | |
| 1071516 | Hs.1407 | EDN2 | Endothelin 2 |
| 592551 | Hs.435714 | PAK1 | P21 protein (Cdc42/Rac)-activated kinase 1 |
| 756565 | Hs.17631 | FZD5 | Frizzled family receptor 5 |
| 826722 | Hs.530595 | STAT2 | Signal transducer and activator of transcription 2, 113kDa |
| 1168508 | Hs.1048 | KITLG | KIT ligand |
| 612577 | Hs.329502 | CASP9 | Caspase 9, apoptosis-related cysteine peptidase |
| 766876 | Hs.375129 | MMP3 | Matrix metallopeptidase 3 (stromelysin 1, progelatinase) |
| 842971 | Hs.412597 | DSG2 | Desmoglein 2 |
| 1467351 | Hs.567319 | POLA1 | Polymerase (DNA directed), alpha 1, catalytic subunit |
| 645774 | In multiple ClusterIDs | | |
| 767295 | Hs.187898 | NF2 | Neurofibromin 2 (merlin) |
| 843225 | In multiple ClusterIDs | | |
| 1473758 | Hs.1722 | IL1A | Interleukin 1, alpha |
| 646846 | Hs.385870 | TDGF1 | Teratocarcinoma-derived growth factor 1 |
| 770862 | Hs.732095 | VEGFB | Vascular endothelial growth factor B |
| 855521 | Hs.406013 | KRT18 | Keratin 18 |
| 1523103 | Hs.633509 | RAD50 | RAD50 homolog (S. cerevisiae) |
| 877782 | Hs.733575 | | Transcribed locus, strongly similar to NP_599193.1 Rac1 gene product [Rattus norvegicus] |
| 624634 | Hs.744 | FDX1 | Ferredoxin 1 |
| 246430 | Hs.285887 | UGT2B4 | UDP glucuronosyltransferase 2 family, polypeptide B4 |
| 844725 | Hs.368214 | TTC3 | Tetratricopeptide repeat domain 3 |
| 590640 | Hs.284491 | PDXK | Pyridoxal (pyridoxine, vitamin B6) kinase |
| 52996 | Hs.516370 | CHST10 | Carbohydrate sulfotransferase 10 |

| | | | |
|---|---|---|---|
| 742576 | Hs.559506 | | SPAG11A |
| 627251 | Hs.532315 | SLC31A1 | Solute carrier family 31 (copper transporters), member 1 |
| 358643 | Hs.23111 | FARSA | Phenylalanyl-tRNA synthetase, alpha subunit |
| 50491 | Hs.13261 | BAI3 | Brain-specific angiogenesis inhibitor 3 |
| 627273 | Hs.239818 | PIK3CB | Phosphatidylinositol-4,5-bisphosphate 3-kinase, catalytic subunit beta |
| 151595 | Data not found | | |
| 590369 | Hs.414795 | SERPINE1 | Serpin peptidase inhibitor, clade E (nexin, plasminogen activator inhibitor type 1), member 1 |
| 754500 | Hs.135087 | IL6R | Interleukin 6 receptor |
| 810142 | In multiple ClusterIDs | | |
| 1035639 | Hs.679430 | CDKL1 | Cyclin-dependent kinase-like 1 (CDC2-related kinase) |
| 592111 | Hs.700779 | | KRT6A |
| 755750 | Hs.463456 | NME2 | NME/NM23 nucleoside diphosphate kinase 2 |
| 42118 | Hs.321709 | P2RX4 | Purinergic receptor P2X, ligand-gated ion channel, 4 |
| 202904 | Hs.309090 | SRSF7 | Serine/arginine-rich splicing factor 7 |
| 306771 | In multiple ClusterIDs | | |
| 380394 | Hs.461178 | EIF1AY | Eukaryotic translation initiation factor 1A, Y-linked |
| 377152 | Hs.528222 | NDUFS4 | NADH dehydrogenase (ubiquinone) Fe-S protein 4, 18kDa (NADH-coenzyme Q reductase) |
| 358850 | Hs.74137 | TMED10 | Transmembrane emp24-like trafficking protein 10 (yeast) |
| 856135 | Hs.443861 | SRPK1 | SRSF protein kinase 1 |
| 45284 | Hs.656905 | LNPEP | Leucyl/cystinyl aminopeptidase |
| 70245 | Hs.459211 | AKAP13 | A kinase (PRKA) anchor protein 13 |
| 950507 | Hs.503886 | ZW10 | Zw10 kinetochore protein |
| 271899 | Hs.35433 | CDC42BPA | CDC42 binding protein kinase alpha (DMPK-like) |
| 491565 | Hs.82071 | CITED2 | Cbp/p300-interacting transactivator, with Glu/Asp-rich carboxy-terminal domain, 2 |
| 67769 | Hs.645288 | KCNK3 | Potassium channel, subfamily K, member 3 |
| 276237 | Hs.592313 | ARHGAP5 | Rho GTPase activating protein 5 |
| 358433 | Hs.26550 | RXRG | Retinoid X receptor, gamma |
| 756931 | Hs.515715 | S100A1 | S100 calcium binding protein A1 |
| 79782 | Hs.463569 | VEZF1 | Vascular endothelial zinc finger 1 |
| 266146 | In multiple | | |

| | | | |
|---|---|---|---|
| | ClusterIDs | | |
| 124781 | Hs.71465 | SQLE | Squalene epoxidase |
| 165878 | Hs.171311 | ITGA8 | Integrin, alpha 8 |
| 470930 | In multiple ClusterIDs | | |
| 30502 | Hs.379912 | DLL1 | Delta-like 1 (Drosophila) |
| 131653 | Hs.411125 | MRPS12 | Mitochondrial ribosomal protein S12 |
| 165857 | Hs.531403 | INPP4B | Inositol polyphosphate-4-phosphatase, type II, 105kDa |
| 128530 | Hs.46701 | SNTB1 | Syntrophin, beta 1 (dystrophin-associated protein A1, 59kDa, basic component 1) |
| 811842 | Hs.237825 | SRP72 | Signal recognition particle 72kDa |
| 752652 | Hs.593995 | TCF7L2 | Transcription factor 7-like 2 (T-cell specific, HMG-box) |
| 810391 | Hs.75619 | HYAL1 | Hyaluronoglucosaminidase 1 |
| 209841 | Hs.432862 | Mar-06 | Membrane-associated ring finger (C3HC4) 6, E3 ubiquitin protein ligase |
| 262996 | Hs.718669 | | Transcribed locus |
| 232933 | Hs.443161 | DPYS | Dihydropyrimidinase |
| 359395 | Hs.464622 | NAPG | N-ethylmaleimide-sensitive factor attachment protein, gamma |
| 281978 | Hs.45002 | RAC3 | Ras-related C3 botulinum toxin substrate 3 (rho family, small GTP binding protein Rac3) |
| 203351 | In multiple ClusterIDs | | |
| 123730 | Hs.316931 | SKAP1 | Src kinase associated phosphoprotein 1 |
| 129644 | Hs.508148 | ABI1 | Abl-interactor 1 |
| 713129 | Hs.90708 | GZMA | Granzyme A (granzyme 1, cytotoxic T-lymphocyte-associated serine esterase 3) |
| 971367 | Hs.512675 | RPS8 | Ribosomal protein S8 |
| 809910 | Hs.374650 | IFITM3 | Interferon induced transmembrane protein 3 |
| 1893775 | Hs.654541 | BGLAP | Bone gamma-carboxyglutamate (gla) protein |
| 825287 | Hs.333791 | TNFSF11 | Tumor necrosis factor (ligand) superfamily, member 11 |
| 293924 | Hs.154495 | ACHE | Acetylcholinesterase |
| 754649 | Hs.204041 | AHSA1 | AHA1, activator of heat shock 90kDa protein ATPase homolog 1 (yeast) |
| 754406 | Hs.172631 | ITGAM | Integrin, alpha M (complement component 3 receptor 3 subunit) |
| 280371 | Hs.149037 | HTR2C | 5-hydroxytryptamine (serotonin) receptor 2C, G protein-coupled |

| | | | |
|---|---|---|---|
| 1404396 | Data not found | | |
| 1468820 | Hs.647092 | NOS3 | Nitric oxide synthase 3 (endothelial cell) |
| 843387 | Hs.329502 | CASP9 | Caspase 9, apoptosis-related cysteine peptidase |
| 346604 | Hs.534342 | AGER | Advanced glycosylation end product-specific receptor |
| 52741 | Hs.26630 | ABCA3 | ATP-binding cassette, sub-family A (ABC1), member 3 |
| 252663 | Hs.65425 | CALB1 | Calbindin 1, 28kDa |
| 841221 | Hs.632015 | ASL | Argininosuccinate lyase |
| 562813 | Hs.89570 | AMPD1 | Adenosine monophosphate deaminase 1 |
| 825312 | Hs.246310 | ATP5J | ATP synthase, H+ transporting, mitochondrial Fo complex, subunit F6 |
| 741880 | Hs.557097 | PBX1 | Pre-B-cell leukemia homeobox 1 |
| 884743 | Hs.653202 | ARF5 | ADP-ribosylation factor 5 |
| 489485 | Hs.277704 | HYOU1 | Hypoxia up-regulated 1 |
| 141768 | Hs.446352 | ERBB2 | V-erb-b2 erythroblastic leukemia viral oncogene homolog 2, neuro/glioblastoma derived oncogene homolog (avian) |
| 855391 | Hs.516807 | STK25 | Serine/threonine kinase 25 |
| 744940 | Hs.98243 | SPINK2 | Serine peptidase inhibitor, Kazal type 2 (acrosin-trypsin inhibitor) |
| 504682 | Hs.279837 | GSTM2 | Glutathione S-transferase mu 2 (muscle) |
| 701412 | In multiple ClusterIDs | | |
| 684879 | In multiple ClusterIDs | | |
| 868308 | Hs.527193 | RPS23 | Ribosomal protein S23 |
| 32493 | Hs.133397 | ITGA6 | Integrin, alpha 6 |
| 663931 | Hs.523852 | CCND1 | Cyclin D1 |
| 428103 | Hs.132448 | CD1C | CD1c molecule |
| 40692 | Hs.22584 | PDYN | Prodynorphin |
| 491727 | Hs.284255 | | ALPP |
| 756549 | Hs.1437 | GAA | Glucosidase, alpha; acid |
| 455121 | Hs.429666 | CEBPG | CCAAT/enhancer binding protein (C/EBP), gamma |
| 298268 | Hs.255935 | BTG1 | B-cell translocation gene 1, anti-proliferative |
| 814798 | In multiple ClusterIDs | | |
| 49318 | Hs.590970 | AXL | AXL receptor tyrosine kinase |
| 586706 | Hs.709196 | CEACAM5 | Carcinoembryonic antigen-related cell adhesion molecule 5 |
| 724831 | Hs.647051 | BCL7B | B-cell CLL/lymphoma 7B |

| | | | |
|---|---|---|---|
| 855624 | Hs.76392 | ALDH1A1 | Aldehyde dehydrogenase 1 family, member A1 |
| 897950 | Hs.291196 | ATP1B1 | ATPase, Na+/K+ transporting, beta 1 polypeptide |
| 854760 | Hs.280342 | PRKAR1A | Protein kinase, cAMP-dependent, regulatory, type I, alpha |
| 360778 | Hs.367437 | | ATM |
| 854879 | Hs.528006 | SPHK2 | Sphingosine kinase 2 |
| 384078 | Hs.106876 | ATP6V0D1 | ATPase, H+ transporting, lysosomal 38kDa, V0 subunit d1 |
| 49560 | Hs.502842 | CAPN1 | Calpain 1, (mu/I) large subunit |
| 183440 | Hs.88251 | ARSA | Arylsulfatase A |
| 993505 | Hs.368325 | SUPT3H | Suppressor of Ty 3 homolog (S. cerevisiae) |
| 1473929 | Hs.105636 | ETV3 | Ets variant 3 |
| 796966 | Hs.462998 | IGFBP4 | Insulin-like growth factor binding protein 4 |
| 2161427 | Hs.32405 | PGR | Progesterone receptor |
| 781959 | Hs.744902 | PHC1 | Polyhomeotic homolog 1 (Drosophila) |
| 2187900 | Hs.66196 | NTHL1 | Nth endonuclease III-like 1 (E. coli) |
| 2534993 | Hs.376208 | LTB | Lymphotoxin beta (TNF superfamily, member 3) |
| 2387733 | Hs.744273 | FAIM3 | Fas apoptotic inhibitory molecule 3 |
| 2522844 | Hs.502872 | MAP3K11 | Mitogen-activated protein kinase kinase kinase 11 |
| 2244482 | Hs.102914 | CUL4B | Cullin 4B |
| 811810 | Hs.270621 | | TAF5L |
| 326252 | Hs.112444 | TAF11 | TAF11 RNA polymerase II, TATA box binding protein (TBP)-associated factor, 28kDa |
| 754650 | Hs.178023 | MYF5 | Myogenic factor 5 |
| 2531717 | Hs.567499 | WT1-AS | WT1 antisense RNA |
| 1939252 | Hs.134623 | RNF7 | Ring finger protein 7 |
| 796105 | Hs.158237 | ITGA10 | Integrin, alpha 10 |
| 758645 | Hs.144309 | PCGF3 | Polycomb group ring finger 3 |
| 2310644 | Hs.339735 | CUL4A | Cullin 4A |
| 531862 | Hs.251531 | PSMA4 | Proteasome (prosome, macropain) subunit, alpha type, 4 |
| 626967 | Hs.37009 | ALPI | Alkaline phosphatase, intestinal |
| 823718 | Hs.100322 | CA6 | Carbonic anhydrase VI |
| 1456424 | Hs.103502 | GPT | Glutamic-pyruvate transaminase (alanine aminotransferase) |
| 265645 | Data not found | | |
| 796613 | Hs.705959 | | Transcribed locus |
| 1456419 | Hs.435615 | CASR | Calcium-sensing receptor |
| 73609 | Hs.507348 | HS3ST1 | Heparan sulfate (glucosamine) 3-O-sulfotransferase 1 |
| 811013 | Hs.82927 | AMPD2 | Adenosine monophosphate deaminase 2 |

| | | | |
|---|---|---|---|
| 1411726 | Hs.153177 | RPS28 | Ribosomal protein S28 |
| 1456120 | Hs.524625 | GRK5 | G protein-coupled receptor kinase 5 |
| 683811 | Hs.367437 | ATM | Ataxia telangiectasia mutated |
| 1544051 | Hs.281902 | SUGT1 | SGT1, suppressor of G2 allele of SKP1 (S. cerevisiae) |
| 2534994 | Hs.130031 | TRIO | Trio Rho guanine nucleotide exchange factor |
| 2149016 | Hs.524871 | POLE | Polymerase (DNA directed), epsilon, catalytic subunit |
| 813810 | In multiple ClusterIDs | | |
| 2516859 | Hs.500066 | TADA2A | Transcriptional adaptor 2A |
| 214990 | Hs.522373 | GSN | Gelsolin |
| 66728 | Hs.365365 | FECH | Ferrochelatase |
| 144834 | In multiple ClusterIDs | | |
| 381812 | Hs.128848 | GRIK3 | Glutamate receptor, ionotropic, kainate 3 |
| 562927 | Hs.471779 | LRRFIP1 | Leucine rich repeat (in FLII) interacting protein 1 |
| 813757 | Hs.433159 | FOLR2 | Folate receptor 2 (fetal) |
| 758343 | Hs.381072 | PPIF | Peptidylprolyl isomerase F |
| 28774 | Hs.446083 | PTPRD | Protein tyrosine phosphatase, receptor type, D |
| 768064 | Hs.72912 | CYP1A1 | Cytochrome P450, family 1, subfamily A, polypeptide 1 |
| 1323432 | Hs.460960 | IDS | Iduronate 2-sulfatase |
| 795254 | In multiple ClusterIDs | | |
| 307873 | Hs.343522 | ATP2B4 | ATPase, Ca++ transporting, plasma membrane 4 |
| 738900 | Hs.144941 | LRRC41 | Leucine rich repeat containing 41 |
| 1323203 | Hs.295917 | ATP6V1B2 | ATPase, H+ transporting, lysosomal 56/58kDa, V1 subunit B2 |
| 244194 | Data not found | | |
| 549139 | Hs.528631 | ARSD | Arylsulfatase D |
| 813637 | Hs.567259 | DNAH9 | Dynein, axonemal, heavy chain 9 |
| 1323448 | Hs.70327 | CRIP1 | Cysteine-rich protein 1 (intestinal) |
| 770868 | Hs.159223 | NAB2 | NGFI-A binding protein 2 (EGR1 binding protein 2) |
| 296757 | Hs.434973 | GYPA | Glycophorin A (MNS blood group) |
| 153340 | Hs.75765 | CXCL2 | Chemokine (C-X-C motif) ligand 2 |
| 197525 | Hs.642706 | FMO5 | Flavin containing monooxygenase 5 |
| 77897 | Hs.567297 | KHK | Ketohexokinase (fructokinase) |
| 45632 | Hs.386225 | GYS1 | Glycogen synthase 1 (muscle) |
| 134748 | Hs.546256 | GCSH | Glycine cleavage system protein H (aminomethyl carrier) |
| 811162 | Hs.519168 | FMOD | Fibromodulin |
| 112629 | Hs.102788 | MAN1A1 | Mannosidase, alpha, class 1A, member 1 |

| | | | |
|---|---|---|---|
| 136235 | Hs.523836 | GSTP1 | Glutathione S-transferase pi 1 |
| 203721 | Hs.654465 | GCLC | Glutamate-cysteine ligase, catalytic subunit |
| 809464 | Hs.533683 | FGFR2 | Fibroblast growth factor receptor 2 |
| 810986 | Hs.82887 | PPP1R11 | Protein phosphatase 1, regulatory (inhibitor) subunit 11 |
| 823928 | In multiple ClusterIDs | | |
| 753467 | Hs.419240 | SLC2A3 | Solute carrier family 2 (facilitated glucose transporter), member 3 |
| 121722 | Hs.519294 | FBN2 | Fibrillin 2 |
| 50506 | Hs.411847 | MAPK6 | Mitogen-activated protein kinase 6 |
| 137940 | Hs.2006 | GSTM3 | Glutathione S-transferase mu 3 (brain) |
| 172440 | Hs.503222 | RAB6A | RAB6A, member RAS oncogene family |
| 951117 | Hs.741179 | SHMT2 | Serine hydroxymethyltransferase 2 (mitochondrial) |
| 785744 | Hs.494538 | PTCH | Patched homolog (Drosophila) |
| 589751 | Hs.90753 | HTATIP2 | HIV-1 Tat interactive protein 2, 30kDa |
| 950710 | Hs.80741 | PCCA | Propionyl CoA carboxylase, alpha polypeptide |
| 797059 | Hs.732006 | | Transcribed locus |
| 594743 | Hs.491148 | PCM1 | Pericentriolar material 1 |
| 840158 | Hs.716502 | | Transcribed locus |
| 730410 | Hs.470627 | LCK | Lymphocyte-specific protein tyrosine kinase |
| 814119 | Hs.463105 | DHX8 | DEAH (Asp-Glu-Ala-His) box polypeptide 8 |
| 146577 | Hs.435001 | KLF10 | Kruppel-like factor 10 |
| 814526 | Hs.236361 | RBM38 | RNA binding motif protein 38 |
| 878838 | Hs.590575 | GRM3 | Glutamate receptor, metabotropic 3 |
| 868380 | Hs.352642 | FCGR2A | Fc fragment of IgG, low affinity IIa, receptor (CD32) |
| 289570 | Hs.372000 | NSMAF | Neutral sphingomyelinase (N-SMase) activation associated factor |
| 430427 | Hs.471991 | MTF1 | Metal-regulatory transcription factor 1 |
| 49987 | Hs.32763 | GRIA2 | Glutamate receptor, ionotropic, AMPA 2 |
| 68557 | Hs.380135 | FABP1 | Fatty acid binding protein 1, liver |
| 305606 | Hs.89839 | EPHA1 | EPH receptor A1 |
| 897880 | Hs.421509 | CCT4 | Chaperonin containing TCP1, subunit 4 (delta) |
| 130843 | Hs.558180 | | MAPK8IP2 |
| 788185 | Hs.521456 | TNFRSF10B | Tumor necrosis factor receptor superfamily, member 10b |
| 109179 | Hs.514276 | SP2 | Sp2 transcription factor |
| 66560 | Hs.625768 | CKAP2 | Cytoskeleton associated protein 2 |
| 85394 | Hs.405156 | PPAP2B | Phosphatidic acid phosphatase type 2B |

| | | | |
|---|---|---|---|
| 745347 | Hs.459940 | LITAF | Lipopolysaccharide-induced TNF factor |
| 711826 | Hs.498661 | USP6NL | USP6 N-terminal like |
| 843121 | Hs.414565 | CLIC1 | Chloride intracellular channel 1 |
| 632074 | In multiple ClusterIDs | | |
| 48285 | Hs.554791 | TP53I11 | Tumor protein p53 inducible protein 11 |
| 24918 | Hs.534371 | PIP5K1B | Phosphatidylinositol-4-phosphate 5-kinase, type I, beta |
| 884539 | Hs.224137 | C9orf114 | Chromosome 9 open reading frame 114 |
| 884655 | Hs.404321 | GARS | Glycyl-tRNA synthetase |
| 842825 | Hs.528780 | GSPT1 | G1 to S phase transition 1 |
| 362910 | Hs.515860 | MAPRE3 | Microtubule-associated protein, RP/EB family, member 3 |
| 470261 | In multiple ClusterIDs | | |
| 810999 | Hs.76686 | GPX1 | Glutathione peroxidase 1 |
| 255333 | Hs.32959 | GRK4 | G protein-coupled receptor kinase 4 |
| 131091 | Hs.478481 | ALG3 | ALG3, alpha-1,3- mannosyltransferase |
| 192271 | Hs.631566 | SYT5 | Synaptotagmin V |
| 277507 | Hs.75652 | GSTM5 | Glutathione S-transferase mu 5 |
| 279790 | In multiple ClusterIDs | | |
| 259842 | Hs.184211 | | PMPCB |
| 361239 | Hs.188553 | RBBP6 | Retinoblastoma binding protein 6 |
| 41195 | Hs.532699 | GCDH | Glutaryl-CoA dehydrogenase |
| 365515 | Hs.567268 | FGF7 | Fibroblast growth factor 7 |
| 41648 | Hs.336046 | IL13RA2 | Interleukin 13 receptor, alpha 2 |
| 418129 | Hs.325978 | | NUMA1 |
| 884644 | Hs.449909 | RPSA | Ribosomal protein SA |
| 1574734 | Hs.142912 | FZD2 | Frizzled family receptor 2 |
| 701492 | Hs.445758 | E2F5 | E2F transcription factor 5, p130-binding |
| 784866 | Hs.99171 | NTF3 | Neurotrophin 3 |
| 937020 | Hs.591016 | CRADD | CASP2 and RIPK1 domain containing adaptor with death domain |
| 1588791 | Hs.501522 | MGMT | O-6-methylguanine-DNA methyltransferase |
| 727246 | Hs.412707 | HPRT1 | Hypoxanthine phosphoribosyltransferase 1 |
| 795325 | In multiple ClusterIDs | | |
| 978834 | Hs.490415 | ZYX | Zyxin |
| 1602843 | Hs.247077 | RHOA | Ras homolog family member A |

| | | | |
|---|---|---|---|
| 742536 | Hs.390250 | FGF12 | Fibroblast growth factor 12 |
| 796724 | Hs.37055 | FGF5 | Fibroblast growth factor 5 |
| 1016755 | Hs.164060 | GRB10 | Growth factor receptor-bound protein 10 |
| 1625688 | Hs.336916 | DAXX | Death-domain associated protein |
| 744917 | Hs.494457 | NINJ1 | Ninjurin 1 |
| 809393 | Hs.497492 | MDM4 | Mdm4 p53 binding protein homolog (mouse) |
| 1032431 | Hs.472497 | BMP8A | Bone morphogenetic protein 8a |
| 1631492 | Hs.506415 | CDK17 | Cyclin-dependent kinase 17 |
| 146868 | Hs.502872 | MAP3K11 | Mitogen-activated protein kinase kinase kinase 11 |
| 51083 | Hs.314543 | CTNND2 | Catenin (cadherin-associated protein), delta 2 |
| 841070 | Hs.213264 | YARS | Tyrosyl-tRNA synthetase |
| 838612 | Hs.118354 | PRR3 | Proline rich 3 |
| 238821 | Hs.584823 | PLA2G7 | Phospholipase A2, group VII (platelet-activating factor acetylhydrolase, plasma) |
| 84713 | Hs.300774 | | FGB |
| 80338 | Hs.632460 | SELENBP1 | Selenium binding protein 1 |
| 856961 | Hs.405590 | EIF3E | Eukaryotic translation initiation factor 3, subunit E |
| 270626 | Hs.696032 | | PPARD |
| 41345 | Hs.205627 | RLF | Rearranged L-myc fusion |
| 837904 | In multiple ClusterIDs | | |
| 884500 | Hs.183958 | PPID | Peptidylprolyl isomerase D |
| 666084 | In multiple ClusterIDs | | |
| 773845 | Hs.266902 | NTF4 | Neurotrophin 4 |
| 877782 | Hs.733575 | | Transcribed locus, strongly similar to NP_599193.1 Rac1 gene product [Rattus norvegicus] |
| 1540499 | Hs.162025 | DCC | Deleted in colorectal carcinoma |
| 685783 | Hs.475055 | BIK | BCL2-interacting killer (apoptosis-inducing) |
| 782835 | Hs.370666 | FOXO1 | Forkhead box O1 |
| 813714 | Hs.390736 | CFLAR | CASP8 and FADD-like apoptosis regulator |
| 23185 | Hs.143250 | TNC | Tenascin C |
| 754479 | In multiple ClusterIDs | | |
| 366154 | In multiple ClusterIDs | | |
| 321708 | Hs.79353 | TFDP1 | Transcription factor Dp-1 |
| 327350 | In multiple | | |

| | | | |
|---|---|---|---|
| | ClusterIDs | | |
| 809946 | Hs.743323 | IFRD2 | Interferon-related developmental regulator 2 |
| 80162 | Hs.412587 | RAD51C | RAD51 homolog C (S. cerevisiae) |
| 845363 | Hs.463456 | NME2 | NME/NM23 nucleoside diphosphate kinase 2 |
| 416316 | Hs.409965 | PNN | Pinin, desmosome associated protein |
| 460673 | Hs.523687 | CLP1 | Cleavage and polyadenylation factor I subunit 1 |
| 47037 | Hs.532316 | DBC1 | Deleted in bladder cancer 1 |
| 629896 | Hs.335079 | MAP1B | Microtubule-associated protein 1B |
| 131626 | Hs.530539 | NFRKB | Nuclear factor related to kappaB binding protein |
| 250069 | Hs.41688 | DUSP8 | Dual specificity phosphatase 8 |
| 796806 | In multiple ClusterIDs | | |
| 25922 | Hs.250712 | CACNB3 | Calcium channel, voltage-dependent, beta 3 subunit |
| 811000 | Hs.514535 | LGALS3BP | Lectin, galactoside-binding, soluble, 3 binding protein |
| 109221 | Hs.591040 | TMEM194A | Transmembrane protein 194A |
| 43743 | In multiple ClusterIDs | | |
| 243675 | Hs.371078 | FAM179B | Family with sequence similarity 179, member B |
| 150702 | Hs.654456 | HOXB5 | Homeobox B5 |
| 123980 | In multiple ClusterIDs | | |
| 248371 | Hs.656374 | | Transcribed locus, weakly similar to XP_002801923.1 PREDICTED: coatomer subunit alpha-like [Macaca mulatta] |
| 360213 | Hs.654519 | KCNAB1 | Potassium voltage-gated channel, shaker-related subfamily, beta member 1 |
| 812246 | Hs.371350 | HLCS | Holocarboxylase synthetase (biotin-(proprionyl-CoA-carboxylase (ATP-hydrolysing)) ligase) |
| 37491 | Hs.515094 | TRIP10 | Thyroid hormone receptor interactor 10 |
| 150314 | Hs.435850 | | Transcribed locus |
| 127928 | Hs.162032 | HBP1 | HMG-box transcription factor 1 |
| 171936 | Hs.632391 | HPCA | Hippocalcin |
| 809494 | Hs.654379 | CD151 | CD151 molecule (Raph blood group) |
| 823679 | Hs.731853 | LDLRAD4 | Low density lipoprotein receptor class A domain containing 4 |
| 198917 | Hs.632262 | FAM134C | Family with sequence similarity 134, member C |
| 129506 | In multiple ClusterIDs | | |
| 300590 | Hs.536122 | | TXNDC9 |
| 243343 | Hs.189772 | CCT2 | Chaperonin containing TCP1, subunit 2 (beta) |

| | | | |
|---|---|---|---|
| 810550 | Hs.78466 | PSMD8 | Proteasome (prosome, macropain) 26S subunit, non-ATPase, 8 |
| 878681 | Hs.400295 | RPL30 | Ribosomal protein L30 |
| 2013956 | Hs.591665 | TIMP4 | TIMP metallopeptidase inhibitor 4 |
| 1756035 | Hs.709179 | TNNI3 | Troponin I type 3 (cardiac) |
| 774078* | Hs.519075 | LMOD1 | Leiomodin 1 (smooth muscle) |
| 814316 | Hs.410817 | | RPL13 |
| 2408099 | Hs.714400 | TAF6L | TAF6-like RNA polymerase II, p300/CBP-associated factor (PCAF)-associated factor, 65kDa |
| 1405136 | Hs.417050 | CCNA1 | Cyclin A1 |
| 878815 | Hs.119177 | ARF3 | ADP-ribosylation factor 3 |
| 488964 | Hs.530461 | HIST2H2AA3 | Histone cluster 2, H2aa3 |
| 2146142 | Hs.49774 | PTPRM | Protein tyrosine phosphatase, receptor type, M |
| 712162 | Hs.146806 | CUL1 | Cullin 1 |
| 768292 | Hs.304192 | *DSTN* | Destrin (actin depolymerizing factor) |
| 740907 | Hs.73722 | APEX1 | APEX nuclease (multifunctional DNA repair enzyme) 1 |
| 154472 | Hs.264887 | FGFR1 | Fibroblast growth factor receptor 1 |
| 82195 | Hs.159509 | SERPINF2 | Serpin peptidase inhibitor, clade F (alpha-2 antiplasmin, pigment epithelium derived factor), member 2 |
| 322914 | Hs.558296 | ACP1 | Acid phosphatase 1, soluble |
| 378813 | Hs.517070 | SLPI | Secretory leukocyte peptidase inhibitor |
| 809421 | Hs.3192 | PCBD1 | Pterin-4 alpha-carbinolamine dehydratase/dimerization cofactor of hepatocyte nuclear factor 1 alpha |
| 1160558 | Hs.503860 | PTS | 6-pyruvoyltetrahydropterin synthase |
| 712460 | In multiple ClusterIDs | | |
| 2240793 | Hs.516646 | CREB1 | CAMP responsive element binding protein 1 |
| 85171 | Hs.652183 | ARF4 | ADP-ribosylation factor 4 |
| 452780 | Hs.502836 | ARL2 | ADP-ribosylation factor-like 2 |
| 23353 | Hs.90791 | GABRA6 | Gamma-aminobutyric acid (GABA) A receptor, alpha 6 |
| 341681 | Hs.438533 | POLI | Polymerase (DNA directed) iota |
| 282977 | Hs.481545 | ADCY2 | Adenylate cyclase 2 (brain) |
| 878545 | Hs.515517 | RPL18 | Ribosomal protein L18 |
| 151104 | Hs.76206 | CDH5 | Cadherin 5, type 2 (vascular endothelium) |
| 2089419 | Hs.649191 | SP1 | Sp1 transcription factor |

| | | | |
|---|---|---|---|
| 884355 | Hs.618732 | WASF3 | WAS protein family, member 3 |
| 50480 | Hs.226007 | ARG2 | Arginase, type II |
| 772304 | Hs.632282 | SLC25A5 | Solute carrier family 25 (mitochondrial carrier; adenine nucleotide translocator), member 5 |
| 487373 | Hs.80986 | ATP5G1 | ATP synthase, H+ transporting, mitochondrial Fo complex, subunit C1 (subunit 9) |
| 770835 | Hs.654441 | BCKDHB | Branched chain keto acid dehydrogenase E1, beta polypeptide |
| 279172 | Hs.315369 | AQP4 | Aquaporin 4 |
| 363055 | Hs.188528 | ADD2 | Adducin 2 (beta) |
| 814353 | Hs.96 | PMAIP1 | Phorbol-12-myristate-13-acetate-induced protein 1 |
| 344430 | Hs.473163 | BMP7 | Bone morphogenetic protein 7 |
| 212188 | Hs.445358 | APOH | Apolipoprotein H (beta-2-glycoprotein I) |
| 81604 | Hs.488007 | AOAH | Acyloxyacyl hydrolase (neutrophil) |
| 66599 | Hs.591847 | NAT1 | N-acetyltransferase 1 (arylamine N-acetyltransferase) |
| 41208 | Hs.1274 | BMP1 | Bone morphogenetic protein 1 |
| 786680 | Hs.480653 | ANXA5 | Annexin A5 |
| 450464 | Hs.470174 | ACVR2A | Activin A receptor, type IIA |
| 200263 | Hs.93194 | | APOA1 |
| 788764 | Hs.57690 | CRYBA4 | Crystallin, beta A4 |
| 853687 | Hs.904 | AGL | Amylo-alpha-1, 6-glucosidase, 4-alpha-glucanotransferase |
| 683662 | Hs.19156 | DAAM1 | Dishevelled associated activator of morphogenesis 1 |
| 2289993 | Data not found | | |
| 2168295 | Hs.431048 | ABL1 | C-abl oncogene 1, non-receptor tyrosine kinase |
| 2222800 | Hs.177766 | PARP1 | Poly (ADP-ribose) polymerase 1 |
| 155 | Hs.525600 | | HSP90AA1 |
| 839552 | Hs.596314 | NCOA1 | Nuclear receptor coactivator 1 |
| 2578588 | Hs.597216 | HIF1A | Hypoxia inducible factor 1, alpha subunit (basic helix-loop-helix transcription factor) |
| 1461158 | Hs.216639 | MSH4 | MutS homolog 4 (E. coli) |
| 157 | Data not found | | |
| 1403166 | Hs.444172 | TRAF6 | TNF receptor-associated factor 6, E3 ubiquitin protein ligase |
| 1309010 | Hs.407604 | MED14 | Mediator complex subunit 14 |
| 1881069 | Hs.386390 | TADA3 | Transcriptional adaptor 3 |
| 255094 | Data not found | | |
| 954356 | Hs.76704 | AR | Androgen receptor |
| 182429 | Hs.194236 | LEP | Leptin |

| | | | |
|---|---|---|---|
| 2168381 | Hs.507475 | RFC1 | Replication factor C (activator 1) 1, 145kDa |
| 2498145 | Hs.660607 | ESR2 | Estrogen receptor 2 (ER beta) |
| 782275 | In multiple ClusterIDs | | |
| 843398 | Hs.73853 | BMP2 | Bone morphogenetic protein 2 |
| 1475659 | Hs.75799 | PRSS8 | Protease, serine, 8 |
| 1455566 | Data not found | | |
| 767419 | In multiple ClusterIDs | | |
| 741988 | Hs.334707 | | ACY1 |
| 1475797 | Hs.476179 | SMARCC1 | SWI/SNF related, matrix associated, actin dependent regulator of chromatin, subfamily c, member 1 |
| 1472150 | Hs.409140 | ATP5O | ATP synthase, H+ transporting, mitochondrial F1 complex, O subunit |
| 768377 | In multiple ClusterIDs | | |
| 665674 | Hs.654542 | BDKRB2 | Bradykinin receptor B2 |
| 1476053 | Hs.631709 | RAD51 | RAD51 homolog (S. cerevisiae) |
| 1325605 | Hs.518595 | NSG1 | Neuron specific gene family member 1 |
| 1712192 | Hs.158560 | TAF1 | TAF1 RNA polymerase II, TATA box binding protein (TBP)-associated factor, 250kDa |
| 1844923 | Hs.409226 | MED12 | Mediator complex subunit 12 |
| 1686512 | Hs.510409 | CCNK | Cyclin K |
| 2312437 | Hs.497487 | PIK3C2B | Phosphatidylinositol-4-phosphate 3-kinase, catalytic subunit type 2 beta |
| 2519221 | Hs.293753 | BOK | BCL2-related ovarian killer |
| 823717 | Data not found | | |
| 681948 | Hs.655995 | FHIT | Fragile histidine triad |
| 126788 | Hs.119825 | SPTA1 | Spectrin, alpha, erythrocytic 1 (elliptocytosis 2) |
| 243399 | Hs.728753 | | GYPE |
| 35185 | Hs.27283 | GABRB1 | Gamma-aminobutyric acid (GABA) A receptor, beta 1 |
| 131839 | Hs.73769 | FOLR1 | Folate receptor 1 (adult) |
| 823943 | Hs.83722 | EPS15 | Epidermal growth factor receptor pathway substrate 15 |
| 60605 | In multiple ClusterIDs | | |
| 774446 | Hs.441047 | ADM | Adrenomedullin |
| 1469234 | Hs.74124 | GPR143 | G protein-coupled receptor 143 |
| 1493390 | Hs.75117 | ILF2 | Interleukin enhancer binding factor 2 |
| 277339 | Hs.523968 | TP53BP2 | Tumor protein p53 binding protein, 2 |

| | | | |
|---|---|---|---|
| 773367 | Hs.370408 | COMT | Catechol-O-methyltransferase |
| 1475633 | Hs.412117 | ANXA6 | Annexin A6 |
| 1456937 | Hs.1154 | OVGP1 | Oviductal glycoprotein 1, 120kDa |
| 897641 | In multiple ClusterIDs | | |
| 853570 | Hs.350927 | SLC25A6 | Solute carrier family 25 (mitochondrial carrier; adenine nucleotide translocator), member 6 |
| 1470333 | Hs.479602 | APBB2 | Amyloid beta (A4) precursor protein-binding, family B, member 2 |
| 1473131 | Hs.332173 | TLE2 | Transducin-like enhancer of split 2 (E(sp1) homolog, Drosophila) |
| 242955 | Hs.533831 | USPL1 | Ubiquitin specific peptidase like 1 |
| 753775 | Hs.484741 | GMPR | Guanosine monophosphate reductase |
| 126368 | Hs.765 | GATA1 | GATA binding protein 1 (globin transcription factor 1) |
| 143519 | Hs.227729 | FKBP2 | FK506 binding protein 2, 13kDa |
| 323506 | Hs.431850 | MAPK1 | Mitogen-activated protein kinase 1 |
| 122636 | Data not found | | |
| 260325 | Hs.473470 | GABPA | GA binding protein transcription factor, alpha subunit 60kDa |
| 302292 | Hs.368404 | EXT2 | Exostosin glycosyltransferase 2 |
| 753215 | Hs.134587 | GNAI1 | Guanine nucleotide binding protein (G protein), alpha inhibiting activity polypeptide 1 |
| 471498 | Hs.334534 | GNS | Glucosamine (N-acetyl)-6-sulfatase |
| 469412 | Hs.592490 | FH | Fumarate hydratase |
| 665774 | Hs.249718 | | EIF4E |
| 44563 | Hs.134974 | GAP43 | Growth associated protein 43 |
| 51362 | Hs.481852 | GTF2B | General transcription factor IIB |
| 308437 | Hs.370858 | FUCA1 | Fucosidase, alpha-L- 1, tissue |
| 34849 | Hs.515070 | EEF2 | Eukaryotic translation elongation factor 2 |
| 71606 | Hs.10306 | NKG7 | Natural killer cell group 7 sequence |
| 823859 | In multiple ClusterIDs | | |
| 810787 | In multiple ClusterIDs | | |
| 701751 | Hs.191482 | CUX1 | Cut-like homeobox 1 |
| 897770 | Hs.405662 | CRABP2 | Cellular retinoic acid binding protein 2 |
| 950096 | Hs.33642 | ARCN1 | Archain 1 |
| 760224 | Hs.98493 | XRCC1 | X-ray repair complementing defective repair in Chinese |

| | | | |
|---|---|---|---|
| | | | hamster cells 1 |
| 795965 | Hs.80691 | CKMT2 | Creatine kinase, mitochondrial 2 (sarcomeric) |
| 204301 | Hs.437705 | CDC25A | Cell division cycle 25A |
| 785975 | Hs.335513 | F13A1 | Coagulation factor XIII, A1 polypeptide |
| 666425 | Hs.472737 | TOP1 | Topoisomerase (DNA) I |
| 898092 | Hs.410037 | CTGF | Connective tissue growth factor |
| 841664 | Hs.74034 | CAV1 | Caveolin 1, caveolae protein, 22kDa |
| 357046 | Hs.1323 | CNGA1 | Cyclic nucleotide gated channel alpha 1 |
| 868332 | Hs.347270 | HLA-DPA1 | Major histocompatibility complex, class II, DP alpha 1 |
| 724893 | Hs.516119 | MOGS | Mannosyl-oligosaccharide glucosidase |
| 183103 | Hs.512856 | SEC14L5 | SEC14-like 5 (S. cerevisiae) |
| 755526 | Hs.122575 | LPAR2 | Lysophosphatidic acid receptor 2 |
| 52881 | Hs.46296 | NHLH2 | Nescient helix loop helix 2 |
| 203003 | Hs.9235 | NME4 | NME/NM23 nucleoside diphosphate kinase 4 |
| 826173 | Hs.494691 | PFN1 | Profilin 1 |
| 739901 | Hs.417077 | CYP51A1 | Cytochrome P450, family 51, subfamily A, polypeptide 1 |
| 714426 | Hs.132370 | CSTF2 | Cleavage stimulation factor, 3' pre-RNA, subunit 2, 64kDa |
| 773301 | Hs.191842 | CDH3 | Cadherin 3, type 1, P-cadherin (placental) |
| 950680 | Hs.290758 | DDB1 | Damage-specific DNA binding protein 1, 127kDa |
| 949938 | Hs.712867 | | Transcribed locus, strongly similar to NP_000090.1 CST3 gene product [Homo sapiens] |
| 724615 | Hs.469723 | RCC1 | Regulator of chromosome condensation 1 |
| 840894 | Hs.369624 | | COX6A1 |
| 45641 | In multiple ClusterIDs | | |
| 841641 | Hs.523852 | CCND1 | Cyclin D1 |
| 898258 | Hs.380138 | CHN1 | Chimerin 1 |
| 789049 | In multiple ClusterIDs | | |
| 796297 | Hs.443134 | GBA2 | Glucosidase, beta (bile acid) 2 |
| 212115 | Hs.154036 | PHLDA2 | Pleckstrin homology-like domain, family A, member 2 |
| 153541 | Hs.116092 | EPHB1 | EPH receptor B1 |
| 884283 | Data not found | | |
| 436741 | Hs.729265 | GPRASP1 | G protein-coupled receptor associated sorting protein 1 |
| 869233 | Hs.134296 | DLC1 | Deleted in liver cancer 1 |
| 970591 | Hs.434102 | HMGB1 | High mobility group box 1 |
| 278483 | In multiple | | |

| | | | |
|---|---|---|---|
| | ClusterIDs | | |
| 455123 | Hs.336994 | MTSS1 | Metastasis suppressor 1 |
| 34102 | Hs.133469 | GOLGA1 | Golgin A1 |
| 971212 | Hs.510172 | HIVEP2 | Human immunodeficiency virus type I enhancer binding protein 2 |
| 151449 | Hs.437040 | PTPN21 | Protein tyrosine phosphatase, non-receptor type 21 |
| 41430 | Hs.145230 | CTIF | CBP80/20-dependent translation initiation factor |
| 22883 | Hs.480085 | TNNI3K | TNNI3 interacting kinase |
| 773568 | Hs.654522 | POU4F1 | POU class 4 homeobox 1 |
| 30175 | Hs.160871 | PTPRO | Protein tyrosine phosphatase, receptor type, O |
| 588609 | Hs.54697 | ARHGEF9 | Cdc42 guanine nucleotide exchange factor (GEF) 9 |
| 796689 | Hs.733076 | NPFF | Neuropeptide FF-amide peptide precursor |
| 300250 | Hs.17987 | TXLNA | Taxilin alpha |
| 413733 | Hs.522632 | TIMP1 | TIMP metallopeptidase inhibitor 1 |
| 1019798 | Data not found | | |
| 175727 | Hs.462529 | TNFRSF25 | Tumor necrosis factor receptor superfamily, member 25 |
| 307342 | In multiple ClusterIDs | | |
| 415851 | Hs.6838 | RND3 | Rho family GTPase 3 |
| 25050 | Hs.124940 | | RND1 |
| 190593 | Hs.166109 | ELAVL2 | ELAV (embryonic lethal, abnormal vision, Drosophila)-like 2 (Hu antigen B) |
| 310406 | Hs.654458 | IL6 | Interleukin 6 (interferon, beta 2) |
| 417226 | Hs.202453 | MYC | V-myc myelocytomatosis viral oncogene homolog (avian) |
| 35356 | Hs.410969 | NTRK3 | Neurotrophic tyrosine kinase, receptor, type 3 |
| 204257 | Hs.591852 | ADAM9 | ADAM metallopeptidase domain 9 |
| 324183 | Hs.467304 | IL11 | Interleukin 11 |
| 429926 | Hs.158932 | APC | Adenomatous polyposis coli |
| 40887 | Hs.159142 | LFNG | LFNG O-fucosylpeptide 3-beta-N-acetylglucosaminyltransferase |
| 240961 | Hs.519873 | DSP | Desmoplakin |
| 324655 | Hs.126256 | IL1B | Interleukin 1, beta |
| 435470 | Hs.714993 | | Transcribed locus |
| 811015 | Data not found | | |
| 590264 | Hs.365706 | MGP | Matrix Gla protein |
| 471642 | Hs.200841 | LAMA2 | Laminin, alpha 2 |

| | | | |
|---|---|---|---|
| 489664 | Hs.229641 | SUB1 | SUB1 homolog (S. cerevisiae) |
| 773330 | Hs.190495 | GPNMB | Glycoprotein (transmembrane) nmb |
| 854444 | Hs.409934 | HLA-DQB1 | Major histocompatibility complex, class II, DQ beta 1 |
| 502355 | Hs.567229 | ACO1 | Aconitase 1, soluble |
| 382564 | Hs.519385 | FOXD1 | Forkhead box D1 |
| 154790 | Hs.415768 | NGFR | Nerve growth factor receptor |
| 810142 | In multiple ClusterIDs | | |
| 853988 | Hs.108104 | UBE2L3 | Ubiquitin-conjugating enzyme E2L 3 |
| 85541 | Hs.524518 | | STAT6 |
| 123755 | Hs.473819 | ERG | V-ets erythroblastosis virus E26 oncogene homolog (avian) |
| 156748 | Hs.145586 | COL4A6 | Collagen, type IV, alpha 6 |
| 290230 | Hs.137427 | IRF8 | Interferon regulatory factor 8 |
| 375833 | Hs.396530 | HGF | Hepatocyte growth factor (hepapoietin A; scatter factor) |
| 291290 | Hs.558402 | SSX4B | Synovial sarcoma, X breakpoint 4B |
| 163561 | Hs.432642 | MAPK12 | Mitogen-activated protein kinase 12 |
| 37234 | Hs.534341 | MAP4K2 | Mitogen-activated protein kinase kinase kinase kinase 2 |
| 156045 | Hs.200600 | SCAMP3 | Secretory carrier membrane protein 3 |
| 811911 | In multiple ClusterIDs | | |
| 727210 | Hs.715837 | | CDNA clone IMAGE:5266573 |
| 193990 | Hs.17614 | ABCB10 | ATP-binding cassette, sub-family B (MDR/TAP), member 10 |
| 49860 | Hs.256667 | PDK2 | Pyruvate dehydrogenase kinase, isozyme 2 |
| 884301 | Hs.557550 | NPM1 | Nucleophosmin (nucleolar phosphoprotein B23, numatrin) |
| 731054 | Hs.195799 | MTTP | Microsomal triglyceride transfer protein |
| 854701 | Hs.643030 | LIPA | Lipase A, lysosomal acid, cholesterol esterase |
| 263836 | Hs.514802 | ZNF24 | Zinc finger protein 24 |
| 845415 | Hs.319334 | NASP | Nuclear autoantigenic sperm protein (histone-binding) |
| 291880 | Hs.389137 | MFAP2 | Microfibrillar-associated protein 2 |
| 878744 | Hs.523512 | TSG101 | Tumor susceptibility gene 101 |
| 755750 | Hs.463456 | NME2 | NME/NM23 nucleoside diphosphate kinase 2 |
| 969748 | Hs.485527 | MUT | Methylmalonyl CoA mutase |
| 878413 | Hs.720597 | | Transcribed locus |
| 755751 | Hs.287362 | TLE3 | Transducin-like enhancer of split 3 (E(sp1) homolog, Drosophila) |
| 292388 | In multiple ClusterIDs | | |

| | | | |
|---|---|---|---|
| 45231 | Hs.657862 | GPR19 | G protein-coupled receptor 19 |
| 109265 | Hs.592591 | ZNF148 | Zinc finger protein 148 |
| 296679 | In multiple ClusterIDs | | |
| 282310 | Hs.530749 | PPFIA1 | Protein tyrosine phosphatase, receptor type, f polypeptide (PTPRF), interacting protein (liprin), alpha 1 |
| 300482 | Hs.98751 | FUBP3 | Far upstream element (FUSE) binding protein 3 |
| 212198 | Hs.523968 | TP53BP2 | Tumor protein p53 binding protein, 2 |
| 327304 | Hs.632447 | DCAF8 | DDB1 and CUL4 associated factor 8 |
| 245330 | Hs.272259 | IGF2 | Insulin-like growth factor 2 (somatomedin A) |
| 308588 | Hs.173464 | FKBP8 | FK506 binding protein 8, 38kDa |
| 46284 | Hs.507669 | FRY | Furry homolog (Drosophila) |
| 33941 | Hs.89655 | PTPRN | Protein tyrosine phosphatase, receptor type, N |
| 201890 | Hs.127799 | BIRC3 | Baculoviral IAP repeat containing 3 |
| 155583 | Hs.531251 | TRAF1 | TNF receptor-associated factor 1 |
| 139540 | Hs.520259 | OSBPL3 | Oxysterol binding protein-like 3 |
| 756847 | Hs.243994 | DEAF1 | DEAF1 transcription factor |
| 345208 | Hs.184492 | ELAVL1 | ELAV (embryonic lethal, abnormal vision, Drosophila)-like 1 (Hu antigen R) |
| 754538 | Hs.356742 | DRAP1 | DR1-associated protein 1 (negative cofactor 2 alpha) |
| 782339 | In multiple ClusterIDs | | |
| 207087 | Hs.465337 | PHLPP1 | PH domain and leucine rich repeat protein phosphatase 1 |
| 121530 | Hs.180535 | | FERMT3 |
| 141627 | Hs.10784 | FAM46A | Family with sequence similarity 46, member A |
| 33051 | In multiple ClusterIDs | | |
| 366341 | In multiple ClusterIDs | | |
| 202607 | Data not found | | |
| 292719 | Hs.16157 | VIPAS39 | VPS33B interacting protein, apical-basolateral polarity regulator, spe-39 homolog |
| 838359 | Hs.77890 | GUCY1B3 | Guanylate cyclase 1, soluble, beta 3 |
| 26566 | In multiple ClusterIDs | | |
| 240945 | Hs.332119 | | SLC22A25 |
| 137581 | Hs.534560 | SLC44A2 | Solute carrier family 44, member 2 |
| 433155 | Hs.30054 | F5 | Coagulation factor V (proaccelerin, labile factor) |
| 745402 | Hs.529862 | CSNK1A1 | Casein kinase 1, alpha 1 |

| | | | |
|---|---|---|---|
| 745214 | Hs.654552 | KDELR2 | KDEL (Lys-Asp-Glu-Leu) endoplasmic reticulum protein retention receptor 2 |
| 293689 | Hs.95612 | DSC2 | Desmocollin 2 |
| 344759 | Hs.172865 | CSTF1 | Cleavage stimulation factor, 3' pre-RNA, subunit 1, 50kDa |
| 80221 | Hs.646 | CPA3 | Carboxypeptidase A3 (mast cell) |
| 214162 | Hs.438462 | MT1H | Metallothionein 1H |
| 121316 | Hs.224012 | *JAG1* | Jagged 1 (Alagille syndrome) |
| 841278 | Hs.484738 | MYLIP | Myosin regulatory light chain interacting protein |
| 66774 | In multiple ClusterIDs | | |
| 609332 | Hs.409662 | COL14A1 | Collagen, type XIV, alpha 1 |
| 135608 | Hs.150122 | OSBPL10 | Oxysterol binding protein-like 10 |
| 66535 | Hs.744924 | OAZ2 | Ornithine decarboxylase antizyme 2 |
| 233365 | Hs.142442 | HP1BP3 | Heterochromatin protein 1, binding protein 3 |
| 42076 | Hs.518123 | TFG | TRK-fused gene |
| 382773 | Hs.621695 | MALAT1 | Metastasis associated lung adenocarcinoma transcript 1 (non-protein coding) |
| 112865 | Hs.26613 | LARP4 | La ribonucleoprotein domain family, member 4 |
| 201986 | Hs.379548 | UBR3 | Ubiquitin protein ligase E3 component n-recognin 3 (putative) |
| 845419 | Hs.744083 | FANCA | Fanconi anemia, complementation group A |
| 153505 | Hs.80552 | DPT | Dermatopontin |
| 83549 | Hs.524224 | C1R | Complement component 1, r subcomponent |
| 415102 | Hs.656 | CDC25C | Cell division cycle 25C |
| 866882 | Hs.593928 | FDFT1 | Farnesyl-diphosphate farnesyltransferase 1 |
| 755228 | Hs.522413 | DNM1 | Dynamin 1 |
| 460470 | Hs.99886 | C4BPB | Complement component 4 binding protein, beta |
| 377461 | In multiple ClusterIDs | | |
| 884867 | Hs.433702 | EIF5 | Eukaryotic translation initiation factor 5 |
| 878468 | Hs.513856 | DPH1 | DPH1 homolog (S. cerevisiae) |
| 713974 | Hs.586219 | CSF1R | Colony stimulating factor 1 receptor |
| 845355 | Hs.128065 | CTSC | Cathepsin C |
| 725321 | Hs.208124 | ESR1 | Estrogen receptor 1 |
| 770880 | Hs.654580 | PRIM2 | Primase, DNA, polypeptide 2 (58kDa) |
| 769959 | Hs.508716 | COL4A2 | Collagen, type IV, alpha 2 |
| 377314 | Hs.82201 | CSNK2A2 | Casein kinase 2, alpha prime polypeptide |

| | | | |
|---|---|---|---|
| 743041 | Hs.380233 | BUD31 | BUD31 homolog (S. cerevisiae) |
| 433162 | Hs.524871 | POLE | Polymerase (DNA directed), epsilon, catalytic subunit |
| 1916687 | Hs.501497 | CD70 | CD70 molecule |
| 2114863 | Hs.514527 | | CDNA clone IMAGE:3354269 |
| 1647954 | Hs.150423 | CDK9 | Cyclin-dependent kinase 9 |
| 1736044 | Hs.647094 | FASTK | Fas-activated serine/threonine kinase |
| 1927303 | Hs.433445 | JAG2 | Jagged 2 |
| 2137170 | Hs.234569 | ZAP70 | Zeta-chain (TCR) associated protein kinase 70kDa |
| 1652077 | Hs.296648 | BMP5 | Bone morphogenetic protein 5 |
| 1742260 | Hs.721234 | Sep-02 | Septin 2 |
| 1932646 | Hs.213424 | SFRP1 | Secreted frizzled-related protein 1 |
| 2145077 | Hs.83722 | EPS15 | Epidermal growth factor receptor pathway substrate 15 |
| 1662329 | Hs.437846 | SMO | Smoothened, frizzled family receptor |
| 1751068 | Hs.158460 | CDK5R2 | Cyclin-dependent kinase 5, regulatory subunit 2 (p39) |
| 1934348 | Hs.161000 | ARID4A | AT rich interactive domain 4A (RBP1-like) |
| 2146427 | Hs.148090 | CDH15 | Cadherin 15, type 1, M-cadherin (myotubule) |
| 1674404 | Hs.120949 | CD36 | CD36 molecule (thrombospondin receptor) |
| 1752379 | Hs.100299 | LIG3 | Ligase III, DNA, ATP-dependent |
| 1949445 | Hs.494312 | NTRK2 | Neurotrophic tyrosine kinase, receptor, type 2 |
| 2157762 | Hs.138211 | MAPK8 | Mitogen-activated protein kinase 8 |
| 592802 | Hs.527061 | RGS12 | Regulator of G-protein signaling 12 |
| 280740 | Hs.654567 | DENND4A | DENN/MADD domain containing 4A |
| 595109 | Hs.503368 | ALG8 | ALG8, alpha-1,3-glucosyltransferase |
| 278101 | In multiple ClusterIDs | | |
| 743278 | Hs.129055 | ODF2 | Outer dense fiber of sperm tails 2 |
| 324715 | In multiple ClusterIDs | | |
| 252382 | Data not found | | |
| 950473 | Hs.600503 | | Transcribed locus, strongly similar to NP_003070.3 SMARCE1 gene product [Homo sapiens] |
| 840803 | In multiple ClusterIDs | | |
| 593183 | Hs.5509 | EVI2B | Ecotropic viral integration site 2B |
| 897595 | Hs.153934 | CBFA2T2 | Core-binding factor, runt domain, alpha subunit 2; translocated to, 2 |

| | | | |
|---|---|---|---|
| 626716 | Hs.192221 | ELL2 | Elongation factor, RNA polymerase II, 2 |
| 1632285 | Hs.654570 | KRT15 | Keratin 15 |
| 1733423 | Hs.435981 | ERCC1 | Excision repair cross-complementing rodent repair deficiency, complementation group 1 (includes overlapping antisense sequence) |
| 1893144 | Hs.356076 | XIAP | X-linked inhibitor of apoptosis |
| 2107532 | Hs.654470 | ITGB7 | Integrin, beta 7 |
| 1643268 | Hs.156316 | DCN | Decorin |
| 1734498 | Hs.533683 | FGFR2 | Fibroblast growth factor receptor 2 |
| 365060 | Hs.321541 | RAB11A | RAB11A, member RAS oncogene family |
| 809992 | In multiple ClusterIDs | | |
| 256664 | Hs.477879 | H2AFX | H2A histone family, member X |
| 210862 | Hs.464137 | ACOX1 | Acyl-CoA oxidase 1, palmitoyl |
| 234237 | Hs.495728 | PIR | Pirin (iron-binding nuclear protein) |
| 184365 | Hs.150423 | CDK9 | Cyclin-dependent kinase 9 |
| 526945 | In multiple ClusterIDs | | |
| 843234 | Hs.90458 | SPTLC1 | Serine palmitoyltransferase, long chain base subunit 1 |
| 837923 | In multiple ClusterIDs | | |
| 839980 | Hs.719203 | SLC37A4 | Solute carrier family 37 (glucose-6-phosphate transporter), member 4 |
| 509887 | Hs.533282 | NONO | Non-POU domain containing, octamer-binding |
| 855872 | Hs.584782 | NRD1 | Nardilysin (N-arginine dibasic convertase) |
| 377048 | Hs.439620 | MYO1B | Myosin IB |
| 595604 | Hs.524599 | NAP1L1 | Nucleosome assembly protein 1-like 1 |
| 135338 | Hs.584833 | TAF1B | TATA box binding protein (TBP)-associated factor, RNA polymerase I, B, 63kDa |
| 511909 | Hs.591853 | CDH17 | Cadherin 17, LI cadherin (liver-intestine) |
| 626555 | Hs.631863 | PNPLA6 | Patatin-like phospholipase domain containing 6 |
| 838744 | Hs.467084 | EIF4G3 | Eukaryotic translation initiation factor 4 gamma, 3 |
| 296587 | Hs.196952 | HLF | Hepatic leukemia factor |
| 108667 | Hs.406277 | SF3A1 | Splicing factor 3a, subunit 1, 120kDa |
| 752732 | In multiple ClusterIDs | | |
| 251019 | Hs.461086 | CDH1 | Cadherin 1, type 1, E-cadherin (epithelial) |
| 246079 | In multiple ClusterIDs | | |
| 295798 | Hs.90061 | PGRMC1 | Progesterone receptor membrane component 1 |

| | | | |
|---|---|---|---|
| 132012 | Hs.443837 | NPEPPS | Aminopeptidase puromycin sensitive |
| 755239 | In multiple ClusterIDs | | |
| 124824 | Hs.546269 | RPL10A | Ribosomal protein L10a |
| 813854 | Hs.443121 | PURA | Purine-rich element binding protein A |
| 49518 | Hs.491988 | TRAM1 | Translocation associated membrane protein 1 |
| 26711 | Hs.591671 | NCBP2 | Nuclear cap binding protein subunit 2, 20kDa |
| 120572 | Hs.370140 | HELZ | Helicase with zinc finger |
| 564621 | Hs.478153 | SERPINI1 | Serpin peptidase inhibitor, clade I (neuroserpin), member 1 |
| 823930 | Hs.124126 | ARPC1A | Actin related protein 2/3 complex, subunit 1A, 41kDa |
| 363144 | Hs.33102 | TFAP2B | Transcription factor AP-2 beta (activating enhancer binding protein 2 beta) |
| 724112 | Hs.98661 | HOXD-AS1 | HOXD cluster antisense RNA 1 |
| 813536 | Hs.464971 | PIK3C3 | Phosphatidylinositol 3-kinase, catalytic subunit type 3 |
| 666128 | Hs.155597 | CFD | Complement factor D (adipsin) |
| 704020 | Hs.592192 | CSF2RB | Colony stimulating factor 2 receptor, beta, low-affinity (granulocyte-macrophage) |
| 83605 | Hs.149252 | CPS1 | Carbamoyl-phosphate synthase 1, mitochondrial |
| 704459 | In multiple ClusterIDs | | |
| 842860 | Hs.654593 | IL10RB | Interleukin 10 receptor, beta |
| 810632 | Hs.420269 | COL6A2 | Collagen, type VI, alpha 2 |
| 511521 | Hs.567968 | CANX | Calnexin |
| 490368 | Hs.34341 | CD58 | CD58 molecule |
| 40017 | Hs.437060 | | CYCS |
| 839991 | Hs.489142 | COL1A2 | Collagen, type I, alpha 2 |
| 40751 | Hs.368322 | CDH8 | Cadherin 8, type 2 |
| 713145 | Hs.502328 | CD44 | CD44 molecule (Indian blood group) |
| 505491 | Hs.474010 | PTTG1IP | Pituitary tumor-transforming 1 interacting protein |
| 51406 | Hs.655498 | SLC13A3 | Solute carrier family 13 (sodium-dependent dicarboxylate transporter), member 3 |
| 324749 | Hs.380133 | ZHX3 | Zinc fingers and homeoboxes 3 |
| 741852 | Hs.531111 | YLPM1 | YLP motif containing 1 |
| 135085 | Hs.632161 | AP3S2 | Adaptor-related protein complex 3, sigma 2 subunit |
| 203732 | Hs.520989 | FGL2 | Fibrinogen-like 2 |
| 51666 | Hs.469872 | ERCC3 | Excision repair cross-complementing rodent repair deficiency, complementation group 3 |

| | | | |
|---|---|---|---|
| 85497 | Hs.408903 | C2 | Complement component 2 |
| 841470 | Hs.148641 | CTSH | Cathepsin H |
| 897531 | In multiple ClusterIDs | | |
| 842849 | Hs.201897 | POLA2 | Polymerase (DNA directed), alpha 2, accessory subunit |
| 898122 | Hs.78065 | C7 | Complement component 7 |
| 774754 | Hs.476018 | CTNNB1 | Catenin (cadherin-associated protein), beta 1, 88kDa |
| 725454 | Hs.83758 | CKS2 | CDC28 protein kinase regulatory subunit 2 |
| 838373 | Hs.191518 | DHX9 | DEAH (Asp-Glu-Ala-His) box polypeptide 9 |
| 85634 | Hs.458355 | C1S | Complement component 1, s subcomponent |
| 795840 | Hs.719698 | | Transcribed locus |
| 840978 | In multiple ClusterIDs | | |
| 397495 | Hs.656653 | CTNNA1 | Catenin (cadherin-associated protein), alpha 1, 102kDa |
| 249603 | Hs.276271 | STK39 | Serine threonine kinase 39 |
| 884719 | Hs.180414 | HSPA8 | Heat shock 70kDa protein 8 |
| 882483 | Hs.7879 | IFRD1 | Interferon-related developmental regulator 1 |
| 448098 | Hs.355581 | ZBTB43 | Zinc finger and BTB domain containing 43 |
| 489823 | Hs.534383 | COX17 | Cytochrome c oxidase assembly homolog 17 (yeast) |
| 772890 | Hs.592121 | RABEP1 | Rabaptin, RAB GTPase binding effector protein 1 |
| 840474 | Hs.286226 | MYO1C | Myosin IC |
| 52926 | Hs.654743 | SRGAP3 | SLIT-ROBO Rho GTPase activating protein 3 |
| 39843 | Hs.26047 | CELF3 | CUGBP, Elav-like family member 3 |
| 178569 | Hs.440896 | SLC9A3R2 | Solute carrier family 9, subfamily A (NHE3, cation proton antiporter 3), member 3 regulator 2 |
| 270505 | Hs.2399 | MMP14 | Matrix metallopeptidase 14 (membrane-inserted) |
| 122298 | Hs.516107 | TET3 | Tet methylcytosine dioxygenase 3 |
| 588559 | Hs.103315 | ZNF384 | Zinc finger protein 384 |
| 306444 | Hs.112444 | TAF11 | TAF11 RNA polymerase II, TATA box binding protein (TBP)-associated factor, 28kDa |
| 471664 | Hs.128702 | FBXO46 | F-box protein 46 |
| 133118 | Hs.277624 | ZZEF1 | Zinc finger, ZZ-type with EF-hand domain 1 |
| 197520 | Hs.592142 | NCOA3 | Nuclear receptor coactivator 3 |
| 342349 | Hs.404183 | MAP3K14 | Mitogen-activated protein kinase kinase kinase 14 |
| 470672 | Hs.132257 | TANK | TRAF family member-associated NFKB activator |
| 79829 | Hs.390729 | ERBB4 | V-erb-a erythroblastic leukemia viral oncogene homolog 4 (avian) |

| | | | |
|---|---|---|---|
| 271662 | Hs.233552 | CDK13 | Cyclin-dependent kinase 13 |
| 346177 | Hs.81134 | IL1RN | Interleukin 1 receptor antagonist |
| 486591 | Hs.269109 | SEMA3C | Sema domain, immunoglobulin domain (Ig), short basic domain, secreted, (semaphorin) 3C |
| 139637 | Hs.2164 | PPBP | Pro-platelet basic protein (chemokine (C-X-C motif) ligand 7) |
| 277305 | Hs.502876 | RHOB | Ras homolog family member B |
| 359976 | Hs.708195 | IFNAR2 | Interferon (alpha, beta and omega) receptor 2 |
| 489519 | Hs.644633 | TIMP3 | TIMP metallopeptidase inhibitor 3 |
| 145844 | Hs.1976 | PDGFB | Platelet-derived growth factor beta polypeptide |
| 285460 | Hs.390171 | COL11A2 | Collagen, type XI, alpha 2 |
| 362834 | Hs.445402 | CDK18 | Cyclin-dependent kinase 18 |
| 491403 | In multiple ClusterIDs | | |
| 151501 | Hs.89640 | TEK | TEK tyrosine kinase, endothelial |
| 287687 | Hs.529400 | IFNAR1 | Interferon (alpha, beta and omega) receptor 1 |
| 365826 | Hs.65029 | GAS1 | Growth arrest-specific 1 |
| 563602 | Hs.408182 | COL2A1 | Collagen, type II, alpha 1 |
| 154720 | Hs.433291 | NAA10 | N(alpha)-acetyltransferase 10, NatA catalytic subunit |
| 855547 | Hs.696211 | HLA-DRB1 | Major histocompatibility complex, class II, DR beta 1 |
| 287687 | Hs.529400 | IFNAR1 | Interferon (alpha, beta and omega) receptor 1 |
| 290091 | Hs.256747 | SPAG8 | Sperm associated antigen 8 |
| 359661 | Hs.183109 | MAOA | Monoamine oxidase A |
| 293925 | In multiple ClusterIDs | | |
| 755578 | Hs.513797 | SLC7A5 | Solute carrier family 7 (amino acid transporter light chain, L system), member 5 |
| 854696 | Hs.521924 | PUF60 | Poly-U binding splicing factor 60KDa |
| 784296 | Hs.163924 | NR3C2 | Nuclear receptor subfamily 3, group C, member 2 |
| 344589 | In multiple ClusterIDs | | |
| 344134 | Hs.348935 | IGLL1 | Immunoglobulin lambda-like polypeptide 1 |
| 244951 | Hs.654604 | PPP5C | Protein phosphatase 5, catalytic subunit |
| 48283 | Hs.654492 | EPHA5 | EPH receptor A5 |
| 246722 | Hs.38449 | SERPINE2 | Serpin peptidase inhibitor, clade E (nexin, plasminogen activator inhibitor type 1), member 2 |
| 341609 | In multiple ClusterIDs | | |

| | | | |
|---|---|---|---|
| 446360 | Hs.472860 | CD40 | CD40 molecule, TNF receptor superfamily member 5 |
| 52096 | Hs.74615 | PDGFRA | Platelet-derived growth factor receptor, alpha polypeptide |
| 267256 | Hs.410037 | CTGF | Connective tissue growth factor |
| 289337 | Hs.510635 | IGHG1 | Immunoglobulin heavy constant gamma 1 (G1m marker) |
| 347434 | Hs.446459 | LEPREL4 | Leprecan-like 4 |
| 823614 | Hs.584809 | TDG | Thymine-DNA glycosylase |
| 364329 | In multiple ClusterIDs | | |
| 768272 | In multiple ClusterIDs | | |
| 27787 | Hs.148909 | CHL1 | Cell adhesion molecule with homology to L1CAM (close homolog of L1) |
| 436554 | Hs.458272 | MPO | Myeloperoxidase |
| 611443 | Hs.517586 | MB | Myoglobin |
| 757440 | In multiple ClusterIDs | | |
| 471859 | Hs.314327 | MEF2D | Myocyte enhancer factor 2D |
| 611255 | Hs.35937 | MYF6 | Myogenic factor 6 (herculin) |
| 273546 | Hs.518774 | PAICS | Phosphoribosylaminoimidazole carboxylase, phosphoribosylaminoimidazole succinocarboxamide synthetase |
| 298417 | Hs.82961 | TFF3 | Trefoil factor 3 (intestinal) |
| 435551 | Hs.443258 | SREBF2 | Sterol regulatory element binding transcription factor 2 |
| 433567 | Hs.105806 | GNLY | Granulysin |
| 447568 | Hs.532632 | ATXN3 | Ataxin 3 |
| 289496 | Hs.437609 | IFIT2 | Interferon-induced protein with tetratricopeptide repeats 2 |
| 857661 | Hs.406423 | SF3B2 | Splicing factor 3b, subunit 2, 145kDa |
| 768205 | In multiple ClusterIDs | | |
| 771000 | Hs.5086 | RBM42 | RNA binding motif protein 42 |
| 510542 | Hs.651244 | GPA33 | Glycoprotein A33 (transmembrane) |
| 26021 | Hs.463928 | DLG4 | Discs, large homolog 4 (Drosophila) |
| 741815 | Hs.644711 | | Transcribed locus |
| 564962 | Hs.522868 | DAZ1 | Deleted in azoospermia 1 |
| 241982 | Hs.57079 | PIP4K2A | Phosphatidylinositol-5-phosphate 4-kinase, type II, alpha |
| 46897 | Hs.30954 | PMVK | Phosphomevalonate kinase |
| 31093 | Hs.654386 | CDH13 | Cadherin 13, H-cadherin (heart) |

| | | | |
|---|---|---|---|
| 280236 | In multiple ClusterIDs | | |
| 727792 | Hs.191046 | PDE1A | Phosphodiesterase 1A, calmodulin-dependent |
| 41356 | Hs.744012 | PPP2R5A | Protein phosphatase 2, regulatory subunit B', alpha |
| 767765 | Hs.654463 | GEM | GTP binding protein overexpressed in skeletal muscle |
| 754355 | Hs.27621 | SEMA5A | Sema domain, seven thrombospondin repeats (type 1 and type 1-like), transmembrane domain (TM) and short cytoplasmic domain, (semaphorin) 5A |
| 361048 | Hs.122523 | SND1 | Staphylococcal nuclease and tudor domain containing 1 |
| 293104 | Hs.498732 | PHYH | Phytanoyl-CoA 2-hydroxylase |
| 563598 | Hs.26225 | GABRP | Gamma-aminobutyric acid (GABA) A receptor, pi |
| 204214 | Hs.405958 | CDC6 | Cell division cycle 6 |
| 66953 | Hs.417029 | ELP5 | Elongator acetyltransferase complex subunit 5 |
| 202722 | Data not found | | |
| 684940 | Hs.267632 | TMF1 | TATA element modulatory factor 1 |
| 230202 | Hs.467239 | ZNF347 | Zinc finger protein 347 |
| 292217 | Hs.434951 | USP15 | Ubiquitin specific peptidase 15 |
| 246543 | Data not found | | |
| 161950 | Hs.483486 | KDM3B | Lysine (K)-specific demethylase 3B |
| 705265 | Hs.729053 | ZMYM6 | Zinc finger, MYM-type 6 |
| 246749 | Hs.744927 | DOCK7 | Dedicator of cytokinesis 7 |
| 565493 | In multiple ClusterIDs | | |
| 67033 | Hs.170205 | LOC729041 | Fatty acid amide hydrolase pseudogene |
| 161484 | Multiple genes | | |
| 366067 | Hs.513430 | CDR2 | Cerebellar degeneration-related protein 2, 62kDa |
| 307471 | Hs.441664 | TSPAN7 | Tetraspanin 7 |
| 123971 | Hs.648635 | DHFR | Dihydrofolate reductase |
| 624754 | Hs.460988 | CBFB | Core-binding factor, beta subunit |
| 739193 | Hs.346950 | CRABP1 | Cellular retinoic acid binding protein 1 |
| 123264 | Hs.479214 | CD38 | CD38 molecule |
| 214816 | In multiple ClusterIDs | | |
| 242820 | In multiple ClusterIDs | | |
| 124052 | Hs.226284 | | Transcribed locus |
| 310356 | Hs.9613 | ANGPTL4 | Angiopoietin-like 4 |
| 119882 | In multiple ClusterIDs | | |
| 195875 | Data not found | | |

| | | | |
|---|---|---|---|
| 120343 | Data not found | | |
| 245444 | Hs.458973 | ZFHX4 | Zinc finger homeobox 4 |
| 129392 | Hs.438673 | | ANKRD13D |
| 195200 | Hs.278064 | ZNF37BP | Zinc finger protein 37B, pseudogene |
| 130008 | Data not found | | |
| 127408 | Hs.515696 | ZNF737 | Zinc finger protein 737 |
| 809828 | Hs.445758 | | E2F5 |
| 295843 | Hs.516700 | CYP27A1 | Cytochrome P450, family 27, subfamily A, polypeptide 1 |
| 366541 | In multiple ClusterIDs | | |
| 486787 | Hs.483454 | CNN3 | Calponin 3, acidic |
| 897006 | Hs.368240 | DYRK1A | Dual-specificity tyrosine-(Y)-phosphorylation regulated kinase 1A |
| 415817 | In multiple ClusterIDs | | |
| 110744 | Hs.730607 | NCAPH2 | Non-SMC condensin II complex, subunit H2 |
| 882548 | Hs.515371 | CAPNS1 | Calpain, small subunit 1 |
| 384015 | Hs.359698 | DDC | Dopa decarboxylase (aromatic L-amino acid decarboxylase) |
| 344997 | Hs.139389 | CST6 | Cystatin E/M |
| 46367 | Hs.77221 | CHKA | Choline kinase alpha |
| 724888 | Hs.436317 | CYP4B1 | Cytochrome P450, family 4, subfamily B, polypeptide 1 |
| 361688 | Hs.490684 | DPP6 | Dipeptidyl-peptidase 6 |
| 130242 | Hs.184298 | CDK7 | Cyclin-dependent kinase 7 |
| 472008 | Hs.301921 | CCR1 | Chemokine (C-C motif) receptor 1 |
| 854401 | Hs.407535 | PRKG1 | Protein kinase, cGMP-dependent, type I |
| 279665 | Hs.502315 | PDHX | Pyruvate dehydrogenase complex, component X |
| 455128 | Hs.1973 | CCNF | Cyclin F |
| 2021468 | Hs.518530 | PAK2 | P21 protein (Cdc42/Rac)-activated kinase 2 |
| 2163351 | Hs.368982 | CASP2 | Caspase 2, apoptosis-related cysteine peptidase |
| 1708905 | Hs.91985 | WNT10B | Wingless-type MMTV integration site family, member 10B |
| 1847162 | Hs.437460 | TP53 | Tumor protein p53 |
| 2062345 | Hs.523329 | EPHB2 | EPH receptor B2 |
| 2168944 | Hs.646917 | FLT4 | Fms-related tyrosine kinase 4 |
| 1711438 | Hs.407995 | MIF | Macrophage migration inhibitory factor (glycosylation-inhibiting factor) |
| 1854033 | Hs.104 | HGFAC | HGF activator |
| 2064198 | Hs.32981 | SEMA3F | Sema domain, immunoglobulin domain (Ig), short basic domain, secreted, (semaphorin) 3F |

| | | | |
|---|---|---|---|
| 825260 | In multiple ClusterIDs | | |
| 1722882 | Hs.467284 | RPS9 | Ribosomal protein S9 |
| 1857026 | Hs.2258 | MMP10 | Matrix metallopeptidase 10 (stromelysin 2) |
| 2090264 | Hs.591873 | IL7 | Interleukin 7 |
| 1283333 | Hs.35758 | KAT6B | K(lysine) acetyltransferase 6B |
| 1723720 | Hs.502659 | RHOC | Ras homolog family member C |
| 1875987 | Hs.643085 | WNT5A | Wingless-type MMTV integration site family, member 5A |
| 2104117 | Hs.473231 | CDH4 | Cadherin 4, type 1, R-cadherin (retinal) |
| 2506249 | Hs.743372 | BAZ1B | Bromodomain adjacent to zinc finger domain, 1B |
| 839374 | Hs.357637 | EXTL2 | Exostosin-like glycosyltransferase 2 |
| 510856 | In multiple ClusterIDs | | |
| 773203 | In multiple ClusterIDs | | |
| 610097 | Hs.10136 | BPHL | Biphenyl hydrolase-like (serine hydrolase) |
| 884272 | Hs.706888 | | Transcribed locus |
| 773383 | Hs.591229 | DLEU1 | Deleted in lymphocytic leukemia 1 (non-protein coding) |
| 611150 | Hs.429 | ATP5G3 | ATP synthase, H+ transporting, mitochondrial Fo complex, subunit C3 (subunit 9) |
| 753794 | Hs.100431 | CXCL13 | Chemokine (C-X-C motif) ligand 13 |
| 770675 | Hs.733403 | TMED2 | Transmembrane emp24 domain trafficking protein 2 |
| 545403 | Hs.114412 | TXNL1 | Thioredoxin-like 1 |
| 839882 | In multiple ClusterIDs | | |
| 627039 | Hs.594351 | ADAM12 | ADAM metallopeptidase domain 12 |
| 1682258 | Hs.367900 | PDCD2 | Programmed cell death 2 |
| 1837472 | Hs.845 | IL13 | Interleukin 13 |
| 2014424 | Hs.159494 | BTK | Bruton agammaglobulinemia tyrosine kinase |
| 2161567 | Hs.93574 | HOXD3 | Homeobox D3 |
| 1704980 | Hs.567260 | DSC1 | Desmocollin 1 |
| 1839737 | Hs.594952 | DES | Desmin |
| 813751 | Hs.591947 | ST3GAL4 | ST3 beta-galactoside alpha-2,3-sialyltransferase 4 |
| 194182 | In multiple ClusterIDs | | |
| 740027 | Hs.13318 | DISC1 | Disrupted in schizophrenia 1 |
| 547058 | Hs.79101 | CCNG1 | Cyclin G1 |

| | | | |
|---|---|---|---|
| 299154 | In multiple ClusterIDs | | |
| 470175 | Hs.6917 | CMC4 | C-x(9)-C motif containing 4 homolog (S. cerevisiae) |
| 1049033 | Hs.86368 | CLGN | Calmegin |
| 784109 | Hs.371199 | SGCE | Sarcoglycan, epsilon |
| 594633 | Hs.210995 | CA12 | Carbonic anhydrase XII |
| 840766 | Hs.495656 | TBL1X | Transducin (beta)-like 1X-linked |
| 593114 | Hs.530477 | SIPA1 | Signal-induced proliferation-associated 1 |
| 842861 | Hs.373763 | HNRNPR | Heterogeneous nuclear ribonucleoprotein R |
| 30473 | Hs.524899 | SAP18 | Sin3A-associated protein, 18kDa |
| 843098 | Hs.201641 | BASP1 | Brain abundant, membrane attached signal protein 1 |
| 725630 | Hs.517216 | PEA15 | Phosphoprotein enriched in astrocytes 15 |
| 842918 | Hs.403917 | FARP1 | FERM, RhoGEF (ARHGEF) and pleckstrin domain protein 1 (chondrocyte-derived) |
| 877664 | In multiple ClusterIDs | | |
| 299559 | Hs.524788 | RAB35 | RAB35, member RAS oncogene family |
| 121621 | Hs.106212 | HNRNPU | Heterogeneous nuclear ribonucleoprotein U (scaffold attachment factor A) |
| 234562 | Hs.210367 | SCAF11 | SR-related CTD-associated factor 11 |
| 511428 | Hs.301350 | FXYD3 | FXYD domain containing ion transport regulator 3 |
| 302591 | Hs.654594 | RHOH | Ras homolog family member H |
| 669443 | Hs.158195 | HSF2 | Heat shock transcription factor 2 |
| 40056 | Hs.513044 | CSPG4 | Chondroitin sulfate proteoglycan 4 |
| 727292 | Hs.504641 | CD163 | CD163 molecule |
| 241880 | Hs.510328 | DDX24 | DEAD (Asp-Glu-Ala-Asp) box polypeptide 24 |
| 193383 | Hs.529609 | ATP13A3 | ATPase type 13A3 |
| 809353 | Hs.75254 | IRF3 | Interferon regulatory factor 3 |
| 72050 | Hs.430733 | CLNS1A | Chloride channel, nucleotide-sensitive, 1A |
| 177737 | Hs.443121 | PURA | Purine-rich element binding protein A |
| 823756 | Hs.509343 | FERMT2 | Fermitin family member 2 |
| 525518 | Hs.386939 | USP7 | Ubiquitin specific peptidase 7 (herpes virus-associated) |
| 810040 | Hs.23119 | TMEM187 | Transmembrane protein 187 |
| 810724 | Hs.76095 | IER3 | Immediate early response 3 |
| 301018 | Hs.659851 | CDKL5 | Cyclin-dependent kinase-like 5 |
| 243741 | Hs.44532 | UBD | Ubiquitin D |

* clones that corresponded to the 'leio-subclass' genes from Nielsen *et al*. (1)
