# Supplementary material for: Gene expression profiling identifies distinct molecular subgroups of leiomyosarcoma with clinical relevance
Source: Br J Cancer. 2016 Sep 8;115(8):1000–7. doi: 10.1038/bjc.2016.280 (PMC5061910; doi:10.1038/bjc.2016.280)
Supplement: Supplementary Table 2 [file bjc2016280x4.pdf]

Supplementary Table S2 Clinical data for tumours in this study

Tumour no	Tumour type[1]	Tumour size[2]	Grade[3]	Site[4]	Location[5]	Specific location	Stage[6]	Tumour subgroup[7]
t704	LR	1	3	b	e	left shoulder	IV	II
t158	P	2	2	b	r	retroperitoneal	III	I
t33107	LR	2	3	b	r	small intestine	III	II
t6858	P	2	3	b	e	hamstring	III	II
t7008	P	2	3	b	r	abdomen/mesentery	IV	I
t4380	P	2	3	b	r	intestine	III	I
t1928	P	2	2	b	e	mid right leg	III	I
t53107	P	2	2	b	r	retroperitoneal	III	I
t9995	P	2	2	a	r	chest wall	IIB	II
t16	P	2	2	b	r	abdomen/mesentery	IV	I
t8749	P	2	3	b	e	left thigh	III	II
t2592	P	2	3	a	e	left buttock	IIB	II
t9652	P	2	3	b	e	left thigh	IV	II
t3779	MET	2	2	m	e	left buttock	IV	I
t278	P	2	nk[8]	nk	e	left calf	IIB	II?
t2868	P	1	3	nk	e	left arm	IIA	II
t9689	LR	1	3	b	e	thigh	IIA	II
t3232	nk	nk	nk	nk	nk	nk	nk	II
t1643	P	2	3	nk	r	left pelvis	III	II
t9118	P	1	1	a	e	right triceps	IA	I

[1] P, Primary tumour; LR, Local recurrence; MET, Metastasis.
[2] 1, 5 cm or less in greatest dimension; 2, more than 5 cm in greatest dimension.
[3] Grade -1, 2 or 3 as in AJCC staging system.
[4] Tumour is superficial(a) or deep (b) as defined in the AJCC staging system.
[5] e, extremity; r, non-extremity (thoracic/abdominal/retroperitoneal sites).
[6] Stage (I to IV) as in AJCC staging system.
[7] Tumour subgroup: I, group I; II, group II.
[8] nk, Not known.
